# Altered Levels of Proteins and Phosphoproteins, in the Absence of Early Causative Transcriptional Changes, Shape the Molecular Pathogenesis in the Brain of Young Presymptomatic Ki91 SCA3/MJD Mouse

**DOI:** 10.1007/s12035-019-01643-4

**Published:** 2019-06-14

**Authors:** Kalina Wiatr, Piotr Piasecki, Łukasz Marczak, Paweł Wojciechowski, Małgorzata Kurkowiak, Rafał Płoski, Małgorzata Rydzanicz, Luiza Handschuh, Johannes Jungverdorben, Oliver Brüstle, Marek Figlerowicz, Maciej Figiel

**Affiliations:** 1 Institute of Bioorganic Chemistry, Polish Academy of Sciences, Z. Noskowskiego 12/14, 61-704 Poznań, Poland; 2 Institute of Computing Science, Poznan University of Technology, Poznań, Poland; 3 Department of Medical Genetics, Medical University of Warsaw, Warsaw, Poland; 4 Institute of Reconstructive Neurobiology, LIFE & BRAIN Center, University of Bonn School of Medicine & University Hospital Bonn, 53127 Bonn, Germany

**Keywords:** Ataxin-3, Mouse, Knock-in, SCA3, MJD, Ataxia, Spinocerebellar, CAG, PolyQ, Presymptomatic, Proteome, Phosphoproteome

## Abstract

Spinocerebellar ataxia type 3 (SCA3/MJD) is a polyQ neurodegenerative disease where the presymptomatic phase of pathogenesis is unknown. Therefore, we investigated the molecular network of transcriptomic and proteomic triggers in young presymptomatic SCA3/MJD brain from Ki91 knock-in mouse. We found that transcriptional dysregulations resulting from mutant ataxin-3 are not occurring in young Ki91 mice, while old Ki91 mice and also postmitotic patient SCA3 neurons demonstrate the late transcriptomic changes. Unlike the lack of early mRNA changes, we have identified numerous early changes of total proteins and phosphoproteins in 2-month-old Ki91 mouse cortex and cerebellum. We discovered the network of processes in presymptomatic SCA3 with three main groups of disturbed processes comprising altered proteins: (I) modulation of protein levels and DNA damage (Pabpc1, Ddb1, Nedd8), (II) formation of neuronal cellular structures (Tubb3, Nefh, p-Tau), and (III) neuronal function affected by processes following perturbed cytoskeletal formation (Mt-Co3, Stx1b, p-Syn1). Phosphoproteins downregulate in the young Ki91 mouse brain and their phosphosites are associated with kinases that interact with ATXN3 such as casein kinase, Camk2, and kinases controlled by another Atxn3 interactor p21 such as Gsk3, Pka, and Cdk kinases. We conclude that the onset of SCA3 pathology occurs without altered transcript level and is characterized by changed levels of proteins responsible for termination of translation, DNA damage, spliceosome, and protein phosphorylation. This disturbs global cellular processes such as cytoskeleton and transport of vesicles and mitochondria along axons causing energy deficit and neurodegeneration also manifesting in an altered level of transcripts at later ages.

**Electronic supplementary material:**

The online version of this article (10.1007/s12035-019-01643-4) contains supplementary material, which is available to authorized users.

## Introduction

Spinocerebellar ataxia type 3 (SCA3), also called Machado–Joseph disease (MJD), is a dominantly inherited genetic disease resulting from the special type of mutation–expansion of CAG repeats in the *ATXN3* gene [1] (MJD and *ATXN3*: OMIM 109150 and 607047). The presence of mutant allele evokes motor abnormalities, such as gait ataxia, ocular symptoms, and later cognitive disturbances, all characteristics for a symptomatic phase of the disease and usually occurring in the third or fourth decade of life [2]. The causative protein to these symptoms is ataxin-3, a protease/deubiquitinase [3, 4], and its mutant version which contains a prolonged stretch of glutamines inside the protein structure [1]. Its natural protease function does not directly imply transcriptional regulation as a mechanism in SCA3 but rather suggests the influence of the mutant ataxin-3 on the level of various kinds of proteins [5]. A number of already identified SCA3 mechanisms also propose affected regulation of processes related to changes in protein level or pathway activation largely independent of transcription. SCA3 pathogenic processes are based on the toxicity of the polyQ tract, proteolytic cleavage of mutant ataxin-3 protein, and accumulation of intranuclear inclusions [6]. Such accumulation is related to dysfunction of proteasome and transport of the protein into the nucleus [7, 8]. In addition, the SCA3 mechanism involves autophagy defects, metabolism and mitochondrial impairment, defective transport along the axons, and dysregulation of intracellular calcium turnover [9–11]. The mechanisms of pathogenesis in SCA3 have been previously thoroughly discussed [12, 13]. Moreover, expanded polyQ stretches alone reside in the nucleus and may bind transcription factors and influence transcriptional activity. Several works also have reported the interaction of ataxin-3 with TBP, CREB-binding protein, p300, MMP-2, and HDAC3 (for review, see [12]).

The already described disease mechanisms and the late occurrence of symptoms in SCA3 patients [6] and models [14, 15] and other neurodegenerative disorders underline the existence of presymptomatic disease phase which consists of molecular and cellular events important to the onset and mechanism of disease. In general, the types of molecular events which probably contribute to the presymptomatic phase and lead to disease pathogenesis are transcriptional changes and alterations in the levels of proteins and their phosphorylation state which all can influence cellular processes [6]. Unfortunately, the disease early presymptomatic phase is presently unknown, and it is also unclear what is the contribution of transcriptional and proteome changes in young SCA3 carriers. To detect potential transcriptional and protein initiators of SCA3 pathology resulting from a direct influence of ataxin-3, we used adult but presymptomatic, young, 2-month-old Ki91 knock-in mouse model homozygous for mutant *Atxn3* gene. The heterozygous version of the Ki91 SCA3/MJD mouse model was already published by us [14]. The present Ki91 mouse is homozygous and contains a higher number of CAG triplet repeats in the mutant Atxn3 gene*.* Now, we have tested the cohort of the 2-month-old homozygous Ki91 animals using several behavioral tests and found no significant motor symptoms at this early stage. Using the animals, we profiled the transcriptome by RNAseq and proteome by mass spectrometry (MS) and phosphoproteome enriched in the cerebellum and cerebral cortex where we search for protein and mRNA changes defining the onset of SCA3. In addition, we performed qPCR profiling of proteomic-based markers and cellular markers in search for late, symptomatic or cell type-dependent transcriptomic dysregulations characteristic for neurodegenerative disease. For targeted late transcriptomic profile, we used 10–14-month-old homozygous Ki91 animals. Subsequently, in the bioinformatics part, we used our dysregulated proteomic hits and performed a systematic identification of processes, pathways, subcellular localization, and discovery cell origin of the dysregulated molecules by Cytoscape/ClueGO/Consensus Path DB and other tools. Our work prioritizes the proteomic changes in response to mutant ataxin-3 as the first molecular events occurring in the brain which lacks changes in the levels of mRNA resulting from the presence of mutant ataxin-3. We demonstrated that the transcriptome changes are secondary in SCA3 and appear later in the disease progression in 10–14-month-old Ki91 animals. Moreover, based on early proteomic changes, we discovered several groups of processes that define the onset of SCA3/MJD in Ki91 animals. Among the processes, we identified disturbed termination of translation, spliceosome phosphorylation, chromatin remodeling, protein phosphorylation, mitochondria organization, DNA damage, axon development, and transport of organelles along the axon.

## Results

### Two-Month-Old Ki91 SCA3/MJD Mice Show Normal Motor Performance, While 14-Month-Old Animals Show Incoordination, Decreased Body Weight, and Symptoms in Scoring Test

**Fig. 1:**
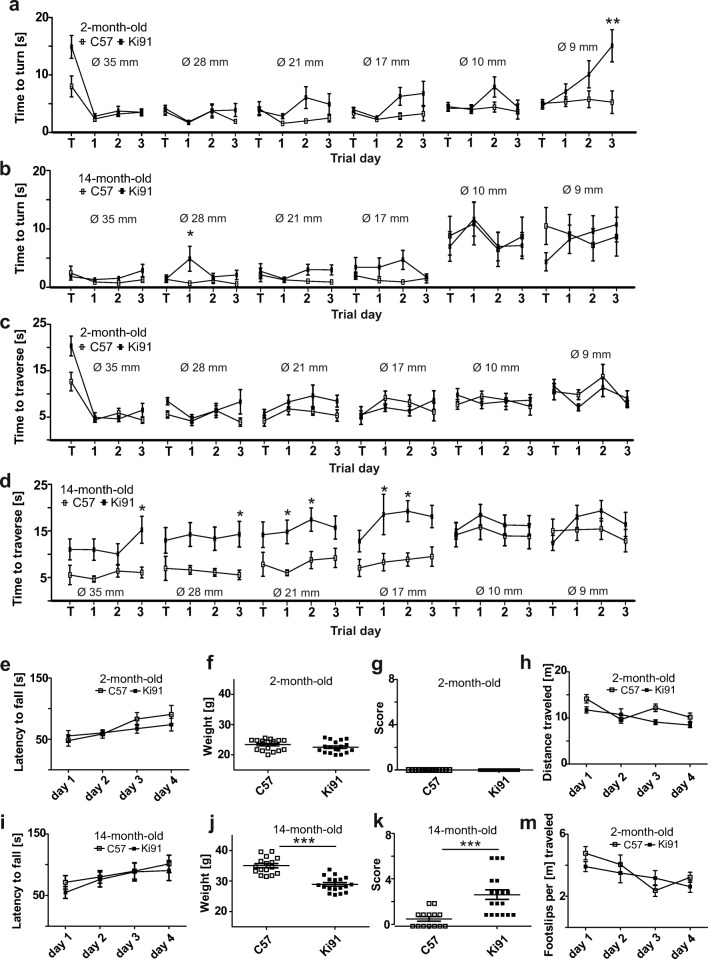
Motor performance of 2-month-old and 14-month-old Ki91 SCA3/MJD mice. Each behavioral test consisted of one training day and three consecutive days of measurement, except of scoring test. In the elevated beam walk (**a**–**d**), two parameters “time to turn” and “traverse time” were tested on each rod (diameter of rods is indicated by *Ø* in mm). Two-month-old Ki91 mice demonstrated significant differences compared to C57BL/6 (C57) in “time to turn” only on the 9-mm rod on the 3rd day of testing (**a**) and no differences in “traverse time” (**c**). Fourteen-month-old Ki91 mice needed significantly more “time to turn” on the 28-mm rod on the first day of testing (**b**) and significantly more “time to traverse” on four rods: 35, 28, 21 and 17 mm (**d**). There were no significant differences for both 2- and 14-month-old mice in rotarod setup which accelerated from 4 to 40 rpm in 9.5 min (**e**, **i**). No significant difference in body weight was observed in 2-month-old Ki91 mice, while 14-month-old Ki91 mice demonstrated significant difference (**f**, **j**). In the scoring test, 2-month-old Ki91 mice scored no parameters associated with SCA3 (**g**), while 14-month-old Ki91 mice scored for characteristic SCA3 phenotypic hallmarks such as: incoordination, gait disturbances, kyphosis, and hind limb clasping (**k**). In the parallel rod floor test, the number of footslips (**m**) and locomotor activity (**h**) was evaluated during 10 min in 2-month-old animals, which demonstrated no differences (**d**). ANOVA with Bonferroni post hoc test (*p* ≤ 0.05; total number of biological replicates: *n* = 36, *n* = 18 per genotype), error bars: SEM

In order to determine if there are any signs of motor incoordination in young 2-month-old Ki91 mice, we performed several tests measuring motor skills. We observed no motor deficits in 2-month-old Ki91 mice on the rotarod and parallel rod floor test (Fig. 1e, h). The scoring test of 2-month-old Ki91 mice demonstrated no signs of incoordination, gait disturbances, kyphosis, muscle weakness, or uncontrolled muscle contraction (Fig. 1g). In elevated beam walk, we evaluated two parameters: time to turn of 180° at the end of the rod and time to traverse the rod, each performed on six rods with decreasing diameter: 35, 28, 21, 17, 10 and 9 mm. We did not detect any statistically significant differences between 2-month-old Ki91 and control mice, except the time to turn on the last rod diameter 9 mm (*p* < 0.01; Bonferroni post hoc test) (Fig. 1a, c). We attributed this result to a novelty effect, not related to the first sign of pathology since the observation was not reproduced on this rod for 14-month-old animals. Furthermore, there were no differences in body weight in young 2-month-old animals (Fig. 1f). In contrast to young animals, 14-month-old Ki91 mice demonstrated significant motor deficits in elevated beam walk test on rod diameter 28 mm in time to turn (*p* < 0.05; Bonferroni post hoc test; Fig. 1b) and on rod diameters 35 mm and 28 mm (*p* < 0.05; Bonferroni post hoc test) and rod diameters 21 and 17 mm in time to traverse (*p* < 0.01; Bonferroni post hoc test) (Fig. 1d). In the scoring test, old animals also demonstrated disturbed gait and coordination, kyphosis, and mild muscle contraction (*p* < 0.001; two-sample *t* test; Fig. 1k). From motor tests, only the rotarod demonstrated no alterations in 14-month-old animals (Fig. 1i). Body weight was significantly lower in 14-month-old animals compared to age-matched controls (*p* < 0.001; two-sample *t* test) (Fig. 1j).

### The Cerebellum and Cerebral Cortex of Young Ki91 SCA3/MJD Mouse Demonstrate the Occurrence of Cells with Accumulation of Atxn3-Positive Signal in the Nuclei

**Fig. 2:**
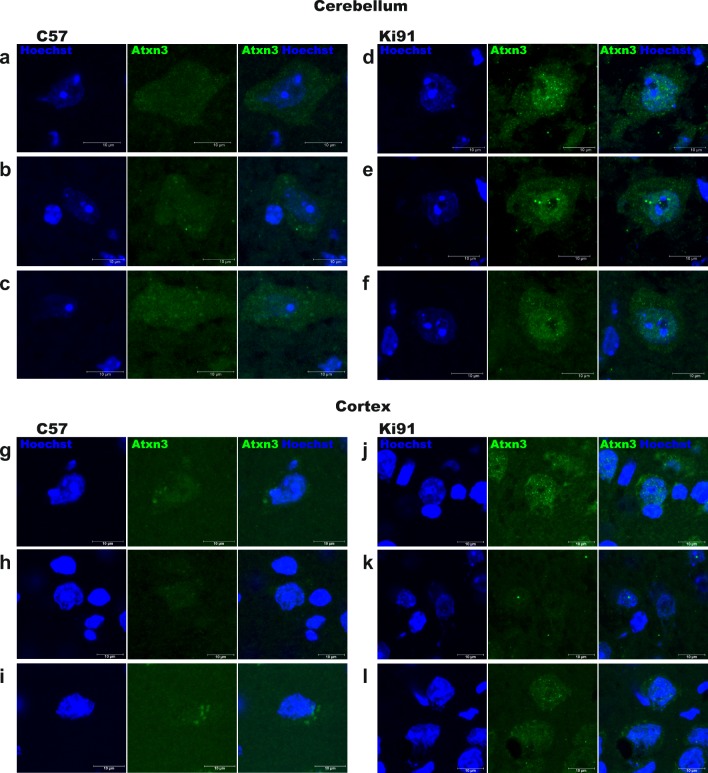
Nuclear localization of ataxin-3 in 2-month-old presymptomatic Ki91 SCA3/MJD mice. The brain sections revealed small number of cells with ataxin-3-positive staining (green; 1H9 antibody) in the white matter of the cerebellum and cerebral cortex where the ataxin-3 localizes mainly in the cell nucleus (blue; Hoechst 33342) of Ki91 mice, whereas in control samples, ataxin-3 localizes uniformly throughout the whole cell. Moreover, the micrograph demonstrates localization of microaggregates in the nucleus of cells in Ki91 mice. The figure demonstrates micrographs of three cells per genotype and brain region; cerebellum of C57BL/6 (C57) (**a**–**c**), cerebellum of Ki91 mice (**d**–**f**) cortex of C57BL/6 (**g**–**i**), and cortex of Ki91 mice (**j**–**l**). Each cell is presented as green and blue fluorescent channel in addition to micrograph with merged channels

One of the cellular hallmarks of SCA3 pathogenesis is the nuclear localization of ataxin-3. Therefore, we investigated ataxin-3 staining of Ki91 2-month-old mouse brain and found cells with nuclei positive for ataxin-3 stained with monoclonal 1H9 anti-ataxin-3 antibody in the white matter of the cerebellum (Fig. 2d–f) and cerebral cortex (Fig. 2j–l). Of note, ataxin-3 accumulation co-localizes with the staining of the nucleus and was restricted to a subset of cells in both regions. Occasionally, the nuclei of young Ki91 mouse cells reveal ataxin-3-positive intranuclear aggregates (Fig. 2e, k). In turn, the localization of ataxin-3 in the cerebellum and cortex of WT animals is uniformly distributed throughout the cytoplasm and cell nucleus (Fig. 2a–c, g–i).

### Transcriptional Changes Related to Mutant Ataxin-3 Do Not Occur in the Cerebellum and Cortex of Young Homozygous Ki91 SCA3/MJD Mouse

**Fig. 3:**
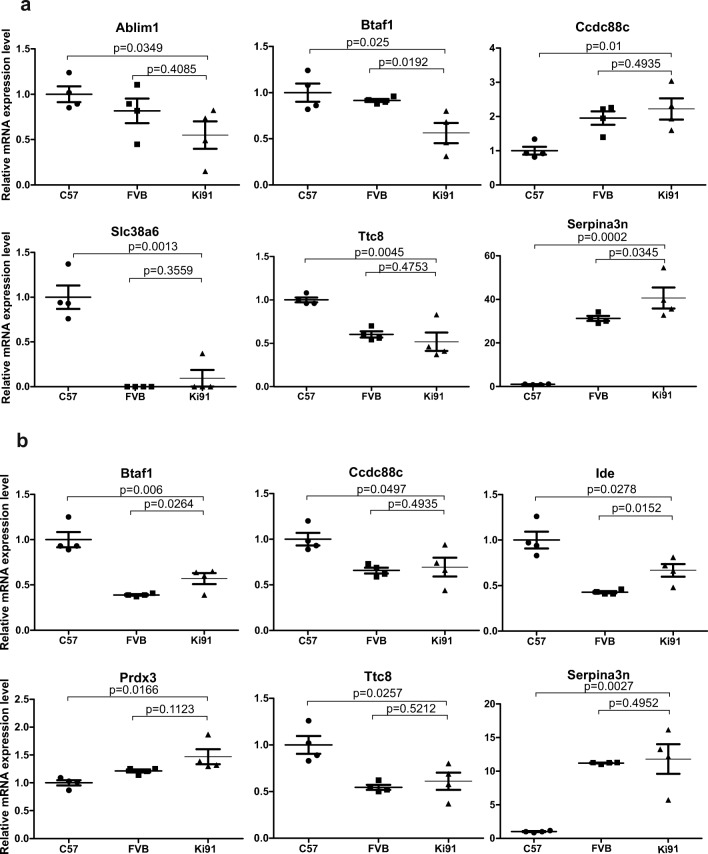
qPCR analysis of RNAseq results reveals lack of transcriptional changes related to mutant ataxin-3 in 2-month-old presymptomatic mice. Genes identified by RNAseq were analyzed by qPCR using control brain tissue collected from C57BL/6 (C57) and FVB mouse strains to exclude the influence of the genetic background (BG) on the level of gene expression. The differences in expression levels of genes would be considered statistically significant if the tested gene demonstrated *p* ≤ 0.01 for each of the brain tissue controls (unpaired Student’s *t* test; error bars: SEM; total number of samples *n* = 12, *n* = 4 per experimental group). However, none of the tested genes from the **a** cerebellum and **b** cerebral cortex consistently reached such significance value across controls and brain tissues. The qPCR results indicate that the differences in expression level measured by RNAseq are the result of genetic background and are not the result of the influence of the mutant ataxin-3. Hence, the presymptomatic cerebellum and cortex from Ki91 mouse do not demonstrate SCA3 causative changes in mRNA levels

**Fig. 4:**
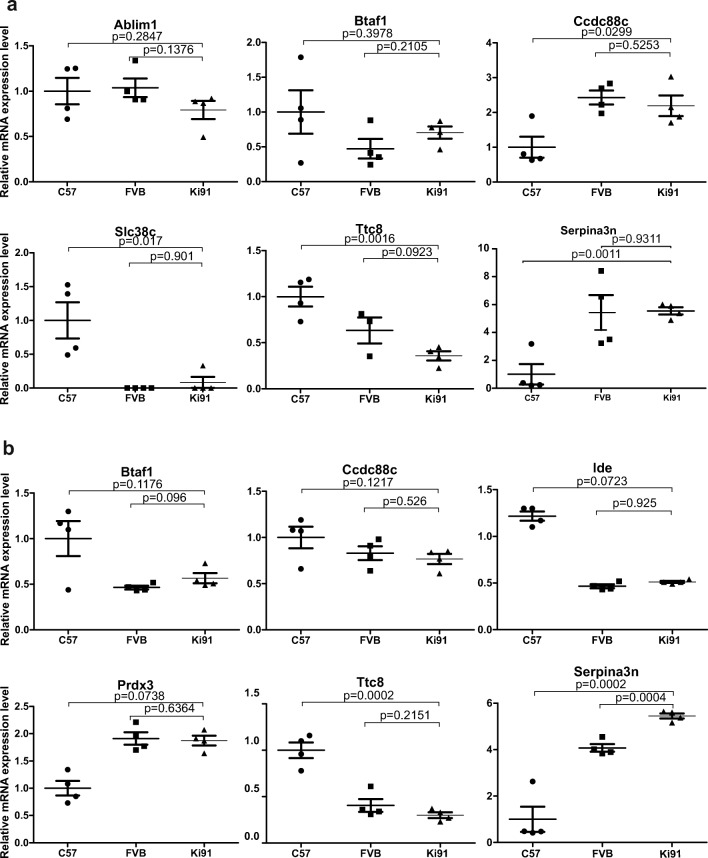
qPCR analysis of RNAseq results reveals lack of transcriptional changes related to mutant ataxin-3 in 4-month-old Ki91 mouse. The presymptomatic changes in mRNA levels of genes identified by RNAseq were examined by qPCR, however using brain tissue from 4-month-old Ki91 mice. The differences in expression levels of genes for the **a** cerebellum and **b** cerebral cortex did not reach consistent statistical significance across controls and tissues (*p* ≤ 0.01 for each of the brain tissue controls in unpaired Student’s *t* test; total number of samples *n* = 12, *n* = 4 per tissue; C57BL6 (C57) or FVB mouse tissue was the control for Ki91 mouse tissues; error bars: SEM)

RNA sequencing was performed using the cerebellum and cortex of four Ki91 mice and four control mice at the age of 2 months. Tables 1 and 2 summarize differentially expressed genes in the cerebellum and cortex, fold changes, *p* values, and adjusted *p* values. The genes significantly dysregulated versus control C57/BL/6 mice were all located within two relatively small “hot spots” on two mouse chromosomes. In the first hot spot located on chromosome 12, we identified dysregulated expression of *Serpina3n*, *Slc38a6*, *Ccdc88c*, *Fbln5*, and *Ttc8*, where all the dysregulated genes are located very close to the targeted *Atxn3* locus. In the second hot spot on chromosome 19, we found dysregulated expression of *Btaf1*, *Ide*, and *Ablim1* which are all located in close proximity in the genome. Such clustering of dysregulated genes in the hot spot loci also closely surrounding the targeted locus may indicate that the loci have different genetic origin than the rest of the Ki91 mouse genome (congenic; C57BL/6) and may stem from a 129sv background which was the source of stem cells where the Ki91 construct was introduced. We have performed a comparison of SNP signatures of *Serpina3n* gene using the RNAseq data on Ki91 mouse and using the MGI database on 129sv, FVB, and C57BL/6 strains. It turned out that the SNP signatures of the *Serpina3n* gene were the same on 129sv, FVB, and Ki91 mice and different from C57BL/6 mouse. This suggested that the region on chromosome 12 near the transgenic *Atxn3* locus was segregating together with the transgene and was selected by genotyping of transgenic *Atxn3* Ki91 animals. As an additional conclusion to our analysis, we would like to state that we no longer consider *Serpina3n* as a SCA3 transcriptional marker as stated previously in [14]. The next step was the investigation of identified transcriptomic changes using real-time qPCR. Therefore, to assess the validity of transcriptional dysregulation in Ki91 mouse and the influence of foreign 129sv mouse background on the expression of dysregulated genes, we examined the cerebellum and cortex from homozygous Ki91 mouse versus a double set of controls including the cerebellum and cortex from FVB mouse strain (same SNP signatures as 129sv) and from C57BL/6 mouse strain (Figs. 3 and 4). The transcriptional dysregulations in Ki91 mouse tissues investigated versus mRNA isolated from C57BL/6 mouse tissues again demonstrated the dysregulation of these genes. A similar analysis did not reveal any change in the level of transcripts when tissues of Ki91 mouse were investigated versus mRNA isolated from tissues of FVB mouse strain. Particularly, a pronounced difference between C57BL/6 and FVB mouse strains was detected for *Serpina3n* where the cerebellum and cortex from C57BL/6 mice demonstrate almost no expression of *Serpina3n* and the cerebellum and cortex from FVB mice demonstrated high levels of *Serpina3n* similar to the one found in Ki91 mice. Other dysregulations of genes in Ki91 mouse revealed by RNAseq also demonstrated dependency on FVB levels in the qPCR cross-control experiment. Detailed fold changes and *p* values are summarized in Table 3. The analysis confirmed that the transcriptomic dysregulation of genes in presymptomatic cerebellum and cortex of Ki91 mouse is the result of genetic background occurring in proximity of transgenic locus and in the locus spot on chromosome 19. In addition, the slightly dysregulated levels of Fgfbp3, Btaf1, and Ide may be the result of known CNV characteristic for C57BL/6 (but not FVB) and covering this region of chromosome 19 [16]. Moreover, the Btaf1 and Ablim1 expression was not altered in symptomatic 14-month-old Ki91 animals (Supplementary Fig. 1). To summarize, we have systematically demonstrated that transcriptional changes related to the direct influence of mutant ataxin-3 protein do not occur in our young presymptomatic Ki91 animals in the cortex and cerebellum. This also may indicate that there are no transcriptomic changes in SCA3 cortex and cerebellum that occur very early in life as a direct result of expansion mutation in *Atxn3*. Our results may also indicate that transcriptomic changes, in general, are not direct triggers for the onset of SCA3.

**Table 1:** List of dysregulated transcripts identified by RNAseq in the cerebellum of 2-month-old Ki91 SCA3/MJD mice

Ensemble ID	Gene ID	Chromosome	Log2 fold change	*p* value	*p* adjusted
ENSMUSG00000021091	Serpina3n	12	4.17	8.4E-202	2E-197
ENSMUSG00000047632	Fgfbp3	19	1.12	1.53E-15	7.33E-12
ENSMUSG00000021182	Ccdc88c	12	1	5.23E-23	4.17E-19
ENSMUSG00000056999	Ide	19	0.95	4.81E-34	5.75E-30
ENSMUSG00000021186	Fbln5	12	0.94	3.82E-13	1.3E-09
ENSMUSG00000092083	Kcnb2	1	0.71	1.83E-08	4.37E-05
ENSMUSG00000040565	Btaf1	19	0.49	1.57E-09	4.69E-06
ENSMUSG00000021062	Rab15	12	− 0.44	2.85E-06	0.004863
ENSMUSG00000044712	Slc38a6	12	− 0.61	1.37E-08	3.64E-05
Additional genes identified by JunctionSeq					
ENSMUST00000079360.11	Ablim1				
ENSMUSG00000021013	Ttc8

**Table 2:** List of dysregulated transcripts identified by RNAseq in cerebral cortex of 2-month-old Ki91 SCA3/MJD mice

Ensemble ID	Gene ID	Chromosome	Log2 fold change	*p* value	*p* adjusted
ENSMUSG00000079012	Serpina3m	12	6.22	4.19E-30	3.34E-26
ENSMUSG00000021091	Serpina3n	12	3.27	3.39E-184	8.11E-180
ENSMUSG00000056999	Ide	19	1.07	1.00E-30	1.20E-26
ENSMUSG00000047632	Fgfbp3	12	0.75	7.53E-06	1.80E-02
ENSMUSG00000040565	Btaf1	19	0.62	6.30E-12	3.77E-08
ENSMUSG00000021182	Ccdc88c	12	− 0.49	4.09E-06	1.22E-02
ENSMUSG00000047415	Gpr68	12	− 0.79	4.03E-06	1.22E-02
Additional genes identified by JunctionSeq					
ENSMUSG00000021013	Ttc8				
ENSMUST00000025961.6	Prdx3

**Table 3:** Table summarizing qPCR validation of RNAseq results using brain tissue from 2- and 4-month-old Ki91 SCA3/MJD versus two control mouse strains (C57BL/6 and FVB)

Gene	Tissue	*p* value C57 vs SCA3	Fold change C57 vs SCA3	*p* value FVB vs SCA3	Fold change FVB vs SCA3
2-month-old					
Ablim1	CERE	0.0349	0.4795	0.4085	0.756189875
Btaf1	CERE	0.025	0.5638	0.0192	0.614754098
Ccdc88c	CERE	0.01	2.225	0.4935	1.136363636
Slc38a6	CERE	0.0013	0.0925	0.3559	1.0925
Ttc8	CERE	0.0045	0.5175	0.4753	0.858921162
Serpina3n	CERE	0.0002	40.66	0.1071	1.30112
Btaf1	CTX	0.006	0.57	0.0264	1.461538
Ccdc88c	CTX	0.0497	0.695	0.7408	1.057034221
Ide	CTX	0.0278	0.6675	0.0146	1.561404
Prdx3	CTX	0.2785	1.228	0.3696	1.16952381
Ttc8	CTX	0.0257	0.61	0.5212	1.119266055
Serpina3n	CTX	0.0027	11.82	0.7911	1.055357143
4-month-old					
Ablim1	CERE	0.2847	0.7922	0.1376	0.763934426
Btaf1	CERE	0.3978	0.7049	1.492167655	1.492167655
Ccdc88c	CERE	0.0299	2.19	0.5253	0.901234568
Slc38a6	CERE	0.017	0.08285	0.901234568	1.08285
Ttc8	CERE	0.0016	0.3577	0.0923	0.565354829
Serpina3n	CERE	0.0011	554.5	0.9311	10.08775731
Btaf1	CTX	0.1176	0.5425	0.096	1.437086093
Ccdc88c	CTX	0.1217	0.7675	0.526	0.924698795
Ide	CTX	0.0723	0.5675	0.924698795	1.220430108
Prdx3	CTX	0.0738	1.416	0.6364	0.947157191
Ttc8	CTX	0.0002	0.3	0.2151	0.740740741
Serpina3n	CTX	0.0002	5.445	0.0004	1.3365243

### Transcriptional Changes in Homozygous, Old Ki91 SCA3/MJD Animals at 10 and 14 Months Old Characterize Affected Cell Types in SCA3

**Fig. 5:**
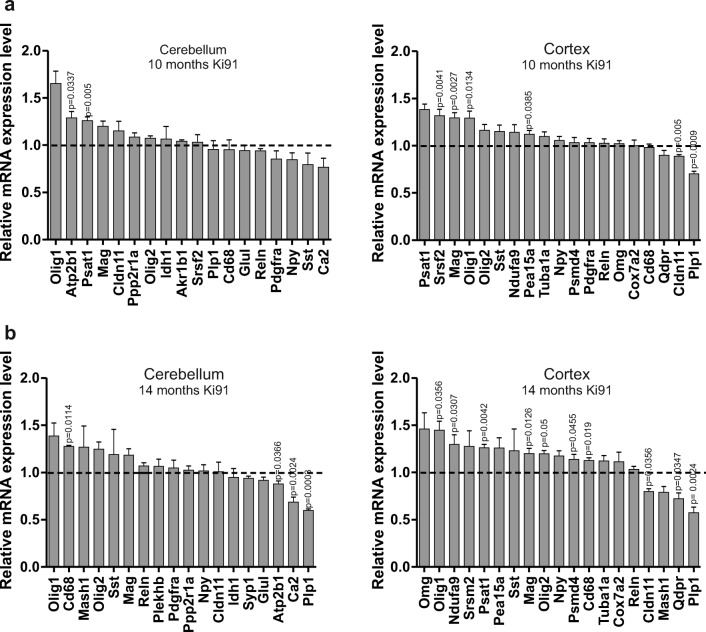
Transcriptomic changes occur in 10- and 14-month-old symptomatic homozygous Ki91 SCA3/MJD mice and are also related to changes associated with particular cell types. In 10-month-old Ki91 mice, the analysis revealed the elevated level of Psat1 and Olig1 in the cerebellum and cortex (**a**). Mag gene related to oligodendrocytes revealed upregulated level in the cortex. Plp1 demonstrated decreased level in the cortex of 10-month-old Ki91 mice suggesting loss of adult oligodendrocytes during disease progression. Fourteen-month-old Ki91 mice demonstrate more pronounced alterations in tested mRNA levels (**b**). In the cerebellum, the Cd68, a microglial marker, demonstrated upregulated expression level. The metabolism-associated genes, Apt2b1 and Ca2, are downregulated. The gene highly expressed in adult oligodendrocytes Plp1 is also downregulated in the cerebellum. In the cortex, the level of transcripts, characteristic for oligodendrocyte precursors, is upregulated (Olig1, Olig2) and, on the other hand, decreased the level of transcripts characteristic for adult oligodendrocytes (Plp1 and Cldn11) and also increased the level of Mag. We did not detect transcriptional changes characteristic for neuronal markers both in 10- and 14-month-old Ki91 mouse brains. We also observe the changed level of genes characteristic for metabolism (Psat1, Qdpr, and Psmd4). *p* ≤ 0.05, using unpaired Student’s *t* test; total number of samples *n* = 8 per age per cerebellum or cortex; *n* = 4 for the control group per individual tissue of 10 or 14 months, *n* = 4 for the SCA3 group per tissue of 10-month-old. In the case of 14-month-old Ki91 mouse, *n* = 3 or *n* = 4 depending on the gene tested: *n* = 3 in the Ki91 mouse group for the following genes in the cerebellum: Srsf2, Ppp2r1a, Idh1, Glul, Atp2b1, Ca2, Plp1; *n* = 3 in Ki91 mouse for the following genes in the cortex: Olig1, Olig2, Cd68, Cox7a2, Reln, Cldn11, Mash, Plp1; *n* = 4 in the Ki91 mouse group in the cerebellum: Olig1, Cd68, Mash1, Olig2, Sst, Mag, Reln, Plekhb1, Pdgfra, Npy, Cldn11; *n* = 4 in the Ki91 mouse group for the following genes in the cortex: Omg, Ndufa9, Srsf2, Psat1, Pea15a, Sst, Mag, Npy, Psmd4, Tuba1a, Qdpr (error bars: SEM)

Since the early transcriptional changes dependent on mutant ataxin-3 do not occur in young Ki91 mice, we sought to determine the transcriptional phenotype in later stages of SCA3. We also asked if the transcriptional changes in the symptomatic phase of SCA3/MJD are markers of any specific cell types in the affected cerebellum and cortex. Identification of such markers could indicate changes in the number of cells in population relevant for brain pathogenesis. Therefore, we made use of old homozygous Ki91 animals at the age of 10 and 14 months. According to our behavioral experiments, 14-month-old Ki91 animals are symptomatic and demonstrate altered motor performance, altered scoring test, and decreased body weight (Fig. 1). Decreased body weight observed in Ki91 animals is one of the hallmarks of SCA3 phenotype in patients [17–20]. Based on preliminary pilot proteomic experiments in old Ki91 mice, we have selected 16 most promising dysregulated protein hits (*Atp2b1*, *Psat1*, *Ppp2r1a*, *Idh1*, *Akr1b1*, *Srsf2*, *Plp1*, *Glul*, *Ca2*, *Ndufa9*, *Pea15a*, *Tuba1a*, *Psmd4*, *Omg*, *Cox7a2*, and *Qdpr*) to examine potential transcriptional events in old Ki91 animals at 10 and 14 months old. In addition to these 16 genes, we also included gene candidates selected based on the scRNAseq database (http://celltypes.brain-map.org/rnaseq/human) as markers of differentiating oligodendrocytes (*Olig1*, *Olig2*), mature oligodendrocytes (*Mag*, *Omg*, *Cldn11*, *Plp1*), microglia (*Cd68*), neurons (*Reln*, *Npy*, *Sst*), and markers of energy metabolism [21, 22]. Table 4 summarizes gene names (total number of 25 genes), fold changes, and *p* values of the qPCR investigation in 10- and 14-month-old Ki91 animals. We found that the mRNA of markers of differentiating oligodendrocytes such as *Olig1* and *Olig2* is elevated in both the cerebellum and cortex of Ki91 mouse indicating an increase of demand for new oligodendrocytes in the SCA3/MJD brain (Fig. 5). Moreover, we have detected decreased markers of myelination such as *Plp1* and *Cldn11* in both the cerebellum and cortex also related to tight junctions and all being markers of mature oligodendrocytes. In addition, we detected dysregulated markers of metabolism such as *Psat1*, *Ndufa9*, *Qdpr*, and *Pea15a* (Fig. 5). There was no change in the expression of neuronal markers such as *Npy*, *Sst*, and *Reln* (Fig. 5). In general, we observed a higher number of dysregulaed genes in animals at the age of 14 months as compared to animals at 10 months old. Together, our observation of transcriptional changes in young and old animals indicates that observed transcriptional changes are related to cell damage and not directly to ataxin-3 effects. The fold changes of all transcriptional changes in old animals did not exceed 1.5 value with the exception of Olig1 in the cerebellum. Since the transcriptional changes first appear in older mice and after changes in the proteome (see next sections), we can conclude that changes in mRNA appear in the brain probably when the compensation of cell degeneration and loss is no longer possible and as a result of previous pathogenic processes.

**Table 4:** Transcriptomic changes in 10- and 14-month-old symptomatic homozygous Ki91 SCA3/MJD mice

10-month-old				14-month-old			
Gene	Tissue	*p* value	Fold change	Gene	Tissue	*p* value	Fold change
Olig1	CERE	0.0054	1.653	Olig1	CERE	0.1147	1.388
Atp2b1	CERE	0.0337	1.29	Cd68	CERE	0.0114	1.278
Psat1	CERE	0.005	1.26	Mesh1	CERE	0.4267	1.268
Mag	CERE	0.0968	1.169	Olig2	CERE	0.0654	1.248
Cldn11	CERE	0.4082	1.153	Sst	CERE	0.5174	1.19
Ppp2r1a	CERE	0.3191	1.085	Mag	CERE	0.1139	1.185
Olig2	CERE	0.0955	1.074	Reln	CERE	0.2437	1.07
Idh1	CERE	0.6792	1.065	Plekhb1	CERE	0.4829	1.065
Akr1b1	CERE	0.3954	1.043	Pdgfra	CERE	0.6852	1.05
Srsf2	CERE	0.7069	1.033	Ppp2r1a	CERE	0.6153	1.027
Plp1	CERE	0.8646	0.955	Npy	CERE	0.8756	1.018
Cd68	CERE	0.7641	0.9541	Cldn11	CERE	0.9458	1.01
Glul	CERE	0.5006	0.9425	Idh1	CERE	0.5816	0.95
Reln	CERE	0.4208	0.9406	Syp1	CERE	0.1438	0.9375
Pdgfra	CERE	0.2705	0.8538	Glul	CERE	0.1805	0.9167
Npy	CERE	0.1851	0.8473	Atp2b1	CERE	0.0366	0.88
Sst	CERE	0.2524	0.7936	Ca2	CERE	0.0024	0.6867
Ca2	CERE	0.0844	0.7675	Plp1	CERE	0.0003	0.5967
Psat1	CTX	0.0006	1.385	Omg	CTX	0.0825	1.46
Srsf2	CTX	0.0041	1.32	Olig1	CTX	0.0356	1.447
Mag	CTX	0.0027	1.294	Ndufa9	CTX	0.0307	1.298
Olig1	CTX	0.0134	1.293	Srsf2	CTX	0.1636	1.278
Olig2	CTX	0.0604	1.164	Psat1	CTX	0.0042	1.263
Sst	CTX	0.0763	1.151	Pea15a	CTX	0.0536	1.26
Ndufa9	CTX	0.1586	1.143	Sst	CTX	0.3694	1.23
Pea15a	CTX	0.0385	1.12	Mag	CTX	0.0126	1.201
Tuba1a	CTX	0.0985	1.098	Olig2	CTX	0.05	1.198
Npy	CTX	0.2445	1.056	Npy	CTX	0.0751	1.174
Psmd4	CTX	0.6667	1.033	Psmd4	CTX	0.0455	1.14
Pdgfra	CTX	0.5033	1.033	Cd68	CTX	0.019	1.126
Reln	CTX	0.6198	1.026	Tuba1a	CTX	0.1073	1.123
Omg	CTX	0.5851	1.023	Cox7a2	CTX	0.4625	1.068
Cox7a2	CTX	1	1	Reln	CTX	0.5893	1.033
Cd68	CTX	0.8199	0.9814	Cldn11	CTX	0.0356	0.7996
Qdpr	CTX	0.2533	0.9	Mash1	CTX	0.0945	0.7891
Cldn11	CTX	0.005	0.8894	Qdpr	CTX	0.0347	0.7225
Plp1	CTX	0.0009	0.7031	Plp1	CTX	0.0024	0.5745

### Transcriptional Changes Occurring in Old Ki91 SCA3/MJD Mouse Also Occur in Neurons from SCA3/MJD Patients

**Fig. 6:**
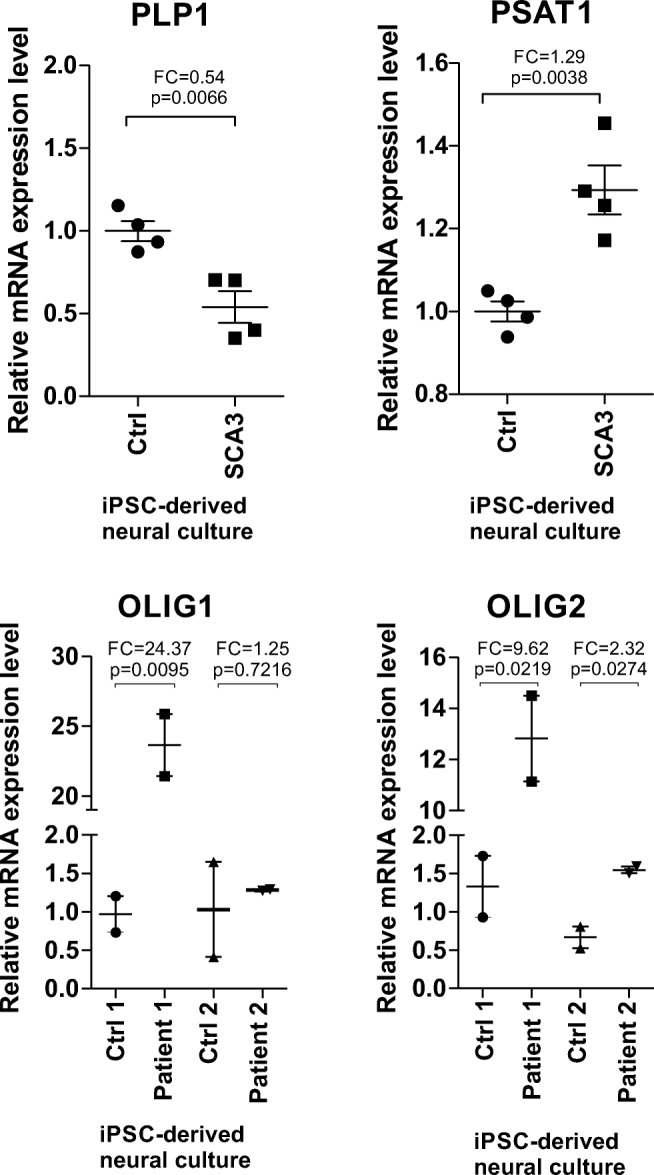
Human SCA3 neural cultures demonstrated mRNA changes similar to late changes in Ki91 SCA3/MJD mouse. iPSC-derived neural cultures from SCA3 patients were tested for dysregulated expression of PLP1, OLIG1, and OLIG2 (classically associated with oligodendrocytes and in neuronal precursors) and for dysregulation of PSAT1 which is associated with serine and glycine metabolism. The marker of precursors of oligodendrocytes and neurons, Olig1, was upregulated in one patient and Olig2 was upregulated in neural cultures from both patients. PLP1, which is highly expressed in mature oligodendrocyte, was decreased in SCA3 patients. The PSAT1 marker was slightly upregulated in both patients. Provisional *p* values (unpaired Student’s *t* test; two cell culture technical replicates per patient) were calculated for the evaluation of differences between patient and control; however, the statistical criteria for using *t* test were not met due to a small number of available patients (patients *n* = 2; unaffected patient controls *n* = 2). PLP1 FC for both patients—0.54, PSAT1 FC for both patients—1.29, OLIG1 patient 1 FC—24.37, patient 2 FC—1.25; OLIG2 patient 1 FC—9.62, patient 2 FC—2.32 (error bars: SEM)

Since the transcriptome changes in our Ki91 mouse model occurred in the older brain, we asked if such changes would also occur in terminally differentiated SCA3 neural culture from SCA3 iPSC. Therefore, we used SCA3 iPSC-derived neural cultures from two SCA3 patients and two genetically related healthy individuals [23]. Neural differentiation comprising 6 weeks therefore represented adult neural cells [24]. The mRNA isolation and qPCR revealed the changes in the genes related to neuronal precursors and oligodendrocytes (*Olig1*, *Olig2*, and *Plp1*) and energy metabolism (*Psat1*) (Fig. 6).

### Early Dysregulations of the Total Proteome in Young Ki91 SCA3/MJD Mouse Cerebellum and Cortex

In the next set of experiments, we asked if any molecular presymptomatic changes exist in the proteome of young Ki91 animals. The processing of mass spectra detected 2753 unique proteins in eight samples of both the cortex and cerebellum (FDR < 0.01, at least two different peptides per protein). After filtering and selection for proteins presenting valid values (see “Methods” section), the number of proteins was reduced to 1098 and 917 proteins in the cerebral cortex and cerebellum, respectively, and these proteins were considered for further analysis in order to detect dysregulated proteins (Supplementary Table 6).

Comparing Ki91 mouse samples to control mouse samples, significant differences (*p* < 0.05; two-sample *t* test) in protein levels were observed for 133 proteins in the cerebral cortex and 93 in the cerebellum (Supplementary Table 6). In the cerebellum, the vast majority of proteins were upregulated (77) and 16 proteins were downregulated in Ki91 mice compared to controls, whereas in the cortex, there were slightly more proteins which were upregulated (76) as compared to downregulated (57) (Supplementary Table 6). The heatmap constructed after filtering of dysregulated proteins demonstrates distinct clustering of the datasets (Supplementary Fig. 2). Interestingly, 14 dysregulated proteins were identified both in the cerebellum and cerebral cortex (Hist1h1d, Trim28, Tubb3, Tubb4a, Tubb5, Rac1, Ran, Arpc3, Uba1, Syngr3, Gnai2, Rap1gds1, Kpnb1, Gcsh).

The lists of all upregulated and downregulated proteins were subjected to separate analysis of GO terms (*p* value cutoff < 0.001) and pathways (at least 10% of dysregulated hits) in CPDB (Tables 5 and 6). In the cerebellum, downregulated proteins are associated mainly with apoptosis, whereas upregulated proteins are involved in metabolism, in particular the citric acid cycle and electron transport chain and carboxylic acids (Table 5). In addition, upregulated proteins in the cerebellum are also implicated in Parkinson disease and microtubules (Table 5). On the contrary, in the cerebral cortex, energy metabolism (oxidative phosphorylation, NADH dehydrogenase activity), Alzheimer disease, and retrograde endocannabinoid signaling involve downregulated proteins (Table 6). Meanwhile, upregulated proteins of the cerebral cortex are associated with microtubules, neuronal projections, membrane trafficking, and transport of synaptic vesicles (Table 6).

**Table 5:** Summary of six top pathways and GO terms separately analyzed for up- and downregulated proteins in the cerebellum (CPDB)

Up/downregulation	GO term/pathway	Source	Number of dysregulated proteins	*p* value	*q* value
Analysis for downregulated proteins					
Apoptosis		Reactome	5	4.47E-08	1.72E-06
Programmed cell death		Reactome	5	5.07E-08	1.72E-06
Activation of BH3-only proteins		Reactome	3	2.47E-06	5.61E-05
Intrinsic pathway for apoptosis		Reactome	3	7.48E-06	0.00012
Alpha6Beta4Integrin		NetPath	3	3.41E-05	0.00046
Apoptotic mitochondrial changes		GO:0008637	3	8.43E-05	0.00455
Analysis for upregulated proteins					
The citric acid (TCA) cycle and respiratory electron transport		Reactome	9	1.10E-07	1.21E-06
Parkinson’s disease—*Homo sapiens* (human)		KEGG	8	3.23E-07	1.78E-06
Metabolism		Reactome	25	9.80E-07	3.59E-06
Carboxylic acid metabolic process		GO:0019752	14	5.87E-06	0.00113
Microtubule		GO:0005874	9	6.90E-06	0.00015
ATP synthesis–coupled electron transport		GO:0042773	5	1.56E-05	0.00117

**Table 6:** Summary of six top pathways and GO terms separately analyzed for up- and downregulated proteins in the cerebral cortex (CPDB)

Up/downregulation	GO term/pathway	Source	Number of dysregulated proteins	*p* value	*q* value
Analysis for downregulated proteins					
Membrane trafficking		Reactome	7	0.00378733	0.02140158
Vesicle-mediated transport		Reactome	7	0.00535039	0.02140158
Microtubule		GO:0005874	9	1.31E-06	1.52E-05
Microtubule cytoskeleton		GO:0015630	14	1.52E-06	1.52E-05
Neuron projection development		GO:0031175	12	5.71E-06	0.00080027
Analysis for upregulated proteins					
Oxidative phosphorylation—*Homo sapiens* (human)		KEGG	7	2.34E-06	2.80E-05
Retrograde endocannabinoid signaling—*Homo sapiens* (human)		KEGG	7	4.76E-06	2.86E-05
Alzheimer’s disease—*Homo sapiens* (human)		KEGG	7	1.23E-05	4.92E-05
NADH dehydrogenase (quinone) activity		GO:0050136	4	2.09E-05	0.0005652
Peptide transport		GO:0015833	19	3.15E-05	0.0022926
Mitochondrial electron transport, NADH to ubiquinone		GO:0006120	4	3.41E-05	0.0022926

### Early Dysregulations of Phosphoproteome in Young Ki91 SCA3/MJD Mouse Cerebellum and Cortex

Following an analysis of total proteome, we asked whether there are any alterations regarding phosphorylation of proteins. Since there are no transcriptional changes in young 2-month-old presymptomatic Ki91 mice, we speculated that dysregulation of protein levels could be related to other cellular mechanisms of protein amount control, such as phosphorylation, which is the most abundant posttranslational modification of proteins. Analysis of mass spectra of enriched samples enabled total identification of 4034 unique proteins. Similar to processing data of total proteins, filtration of valid values and statistical tests were performed on the results obtained in the analysis of the phosphoproteome. The analysis revealed significant differences in the phosphorylation level of 82 individual proteins in the cerebellum and 335 individual proteins in the cerebral cortex (Supplementary Table 6). Part of those proteins had altered modification level in more than one site which increased the number of hits (total number of altered phosphorylation sites: cerebellum = 95, cortex = 481). In both the cerebral cortex and cerebellum, we observed that the phosphorylation levels of dysregulated proteins were decreased and phosphorylation of only three residues in three proteins was increased (cerebellum: Hist1h1d; cerebral cortex: Prkcg and Pclo). Notably, the majority of dysregulated phosphoproteins in the cerebellum overlap with the list of dysregulated phosphoproteins in the cerebral cortex (68% of individual cerebellar phosphoproteins overlap with the cortex; *N* = 56 and 73% cerebellar phosphosites overlap with the cortex; *N* = 70) (Supplementary Table 6).

**Fig. 7:**
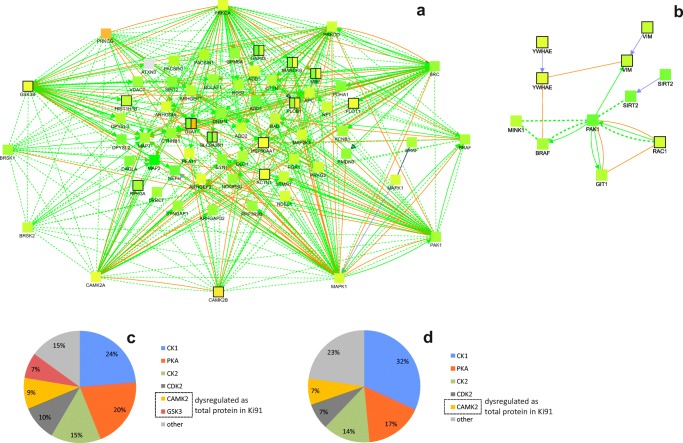
Dysregulation of protein phosphorylation in Ki91 SCA3/MJD mouse brains: kinases and their substrates. Network of dysregulated kinases and their substrates was generated with the CPDB tool: induced network modules using gene names coding for dysregulated proteins and phosphoproteins (high and medium confidence), number of dysregulated proteins and phosphoproteins in the cerebellum *n* = 175; in the cerebral cortex *n* = 486, four biological replicates (*Z*-score calculated with the binomial proportions test). The gradient of red color is for upregulated proteins, green color for downregulated proteins. Nonframed squares denote proteins with altered phosphorylation; one-color, framed squares denote total dysregulated proteins; and two-color, framed squares denote proteins, which are dysregulated both at phosphorylation and total levels (left part of the square—phosphorylation, right part of the square—total level). Orange arrows are for biochemical reactions and green arrows for physical interactions. Kinases with the highest number of dysregulated substrates are arranged in the outer space of the network. There are several such kinases identified in the cerebral cortex (**a**) and one kinase: Pak1 in the cerebellum (**b**). In the cerebral cortex, the substrate, which is modified by several different dysregulated kinases, is Mapt and Map2, both regulating microtubule functions. Further analysis of peptides with modified phosphosites using PHOSIDA revealed additional kinases (CKs, Pka, Cdks, Camk2a) common for the cerebellum and cerebral cortex, which phosphorylate the highest number of dysregulated phosphoproteins in the cerebral cortex, *n* = 335 (**c**), and cerebellum, *n* = 82 (**d**); total number of biological replicates: *n* = 8, *n* = 4 per genotype

We have constructed a network of dysregulated kinases with the highest number of substrates among dysregulated proteins (altered total levels or phosphorylation pattern) using the CPDB tool, protein–protein interaction networks (induced network) visualized in Fig. 7. The network of proteins of the cerebral cortex separates the most important kinases, which are arranged in the outer space, circularly around their substrates (Fig. 7a). This arrangement demonstrates that most of the identified dysregulated kinases are interactors (CPDB) and have an influence on common protein substrates which were identified by us as dysregulated in total proteome or phosphoproteome analysis. The smaller network organizing proteins of the cerebellum places the kinase Pak1 in the center of protein–protein interactions (Fig. 7b). Pak1 has a role in cellular pathways related mainly to cytoskeleton organization and transport, proliferation, and apoptosis [25]. In the cerebral cortex, Pak1 is also one of the most compelling kinases, together with Brsk1 and Brsk2 also playing role in axon guidance, Src, Braf, and multifunctional Mapk and Gsk3b (Fig. 7a). Other important kinases of the network are involved in trans-synaptic signaling: calcium/calmodulin-dependent kinases Camk2a and Camk2b and isoforms of protein kinase c: Prkca, Prkcd, and Prkcg (Fig. 7a). On the other hand, substrates which may be modified by the highest number of kinases in the network are MAPT and MAP2—both regulating functions of the microtubule of axons and dendrites.

Furthermore, we have performed another analysis with the use of PHOSIDA in order to find those kinases, which are not necessarily dysregulated, but which phosphorylate dysregulated proteins at identified residues within motifs. The analysis identified five kinases, namely Ck1 and 2, Pka, Cdk2, and Camk2 for both the cerebellum and cerebral cortex (Fig. 7c, d), which are mainly responsible for the altered level of phosphorylation on most residues and motifs (77% in the cerebellum, 78% in the cerebral cortex).

In addition, comparison of total proteome and phosphoproteome identified 7 proteins in the cerebellum which were altered commonly (Hist1h1d, Uba1, Nefh, Tmpo, Trim28, Dync1i1, Add3) and 19 such proteins in the cerebral cortex (Basp1, Slc9a3r1, Dync1li2, Gap43, Mbp, Slc6a17, Marcks, Stx1b, Ndrg2, Kif5c, Map1b, Uba1, Pcdh1, Plcb1, Tomm70a, Eef1d, Trim28, Sept4, Gja1) (Supplementary Table 6). However, in both the cerebral cortex and cerebellum, there were groups of biological processes containing dysregulated phosphoproteins, which correspond well with groups of processes containing dysregulated total proteins (Supplementary Tables 3–5).

### Western Blot Analysis of Dysregulated Proteins and Phosphoproteins in Presymptomatic Ki91 SCA3/MJD Mice

**Fig. 8:**
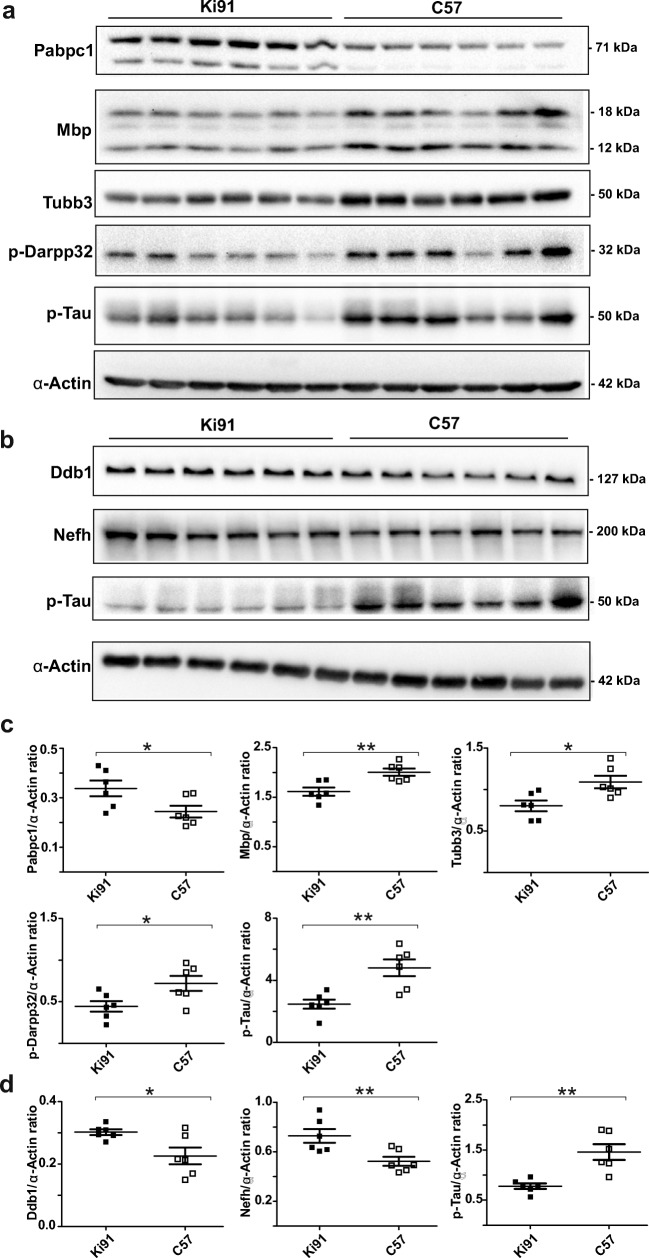
Western blotting analysis of proteins and phosphoproteins dysregulated in presymptomatic 2-month-old Ki91 mice. Western blot analysis confirmed increased levels of Pabpc1 (*p* = 0.013; two-sample *t* test) and decreased levels of Mbp (*p* = 0.0012; two-sample *t* test), Tubb3 (*p* = 0.011; two-sample *t* test), p-Darpp32 (*p* = 0.045; two-sample *t* test), and p-Tau (*p* = 0.0012; two-sample *t* test) in the cerebral cortex of 2-month-old Ki91 mice (**a**, **c**). In the cerebellum of Ki91 animals, increased levels of Ddb1 (*p* = 0.011; two-sample *t* test) and Nefh (*p* = 0.007; two-sample *t* test) and decreased levels of p-Tau (*p* = 0.0035; two-sample *t* test) were demonstrated (**b**, **d**). α-Actin was used as a loading control. *N* = 6 per genotype, error bars: SEM. All experiments were performed in three technical replicates

The selected proteins and phosphoproteins identified in label-free mass spectrometry analysis were subjected to western blot analysis using commercial antibodies. The criteria for validation of proteins were the fold change of dysregulation, the affected processes, and in the case of phosphoproteins, also the commercial availability of the phospho-specific antibodies. p-Darpp32, p-Tau, Pabpc1, Mbp, Tubb3, Ddb1, and Nefh were assayed versus α-actin as loading control (Fig. 8). We confirmed the downregulation of p-Darpp32 (phosphosite Ser97; fold change (FC) = 0.62; *p* = 0.045; two-sample *t* test) in the cerebral cortex and p-Tau in the cerebral cortex (FC = 0.5; *p* = 0.0012; two-sample *t* test) (Fig. 8a, c) and cerebellum (FC = 0.58; *p* = 0.0035; two-sample *t* test) (Fig. 8b, d). Furthermore, we selected several relevant proteins related to such biological processes as translation [26] (Pabpc1), DNA damage and repair [27] (Ddb1), myelin formation [28] (Mbp), and neuronal microtubule function [29, 30] (Tubb3, Nefh). We confirmed increased levels of Pabpc1 (FC = 1.5; *p* = 0.013; two-sample *t* test) and decreased levels of Mbp (FC = 0.78; *p* = 0.0012; two-sample *t* test) and Tubb3 (FC = 0.78; *p* = 0.011; two-sample *t* test) in the cerebral cortex (Fig. 8a, c) as well as increased levels of Ddb1 (FC = 1.34; *P* = 0.011; two-sample *t* test) and Nefh (FC = 1.7; *p* = 0.007; two-sample *t* test) in the cerebellum (Fig. 8b, d) (*p* < 0.05; two-sample *t* test).

### Dysregulations of Total Proteome and Phosphoproteome Reveal Three Major Arbitrary Groups of Disturbed Biological Processes in the Brain of Young KI91 SCA3/MJD Mouse

**Fig. 9:**
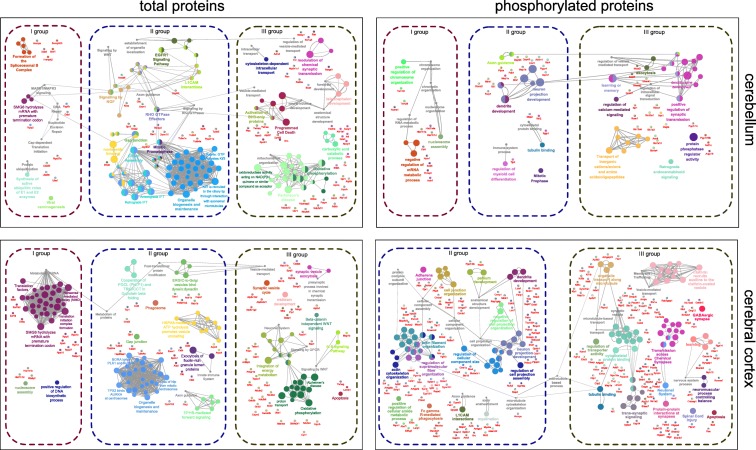
Major groups of affected biological processes based on the proteomic and phosphoproteomic analysis in Ki91 SCA3/MJD mouse brains. A network of GO terms and pathways (two-sided hypergeometric test with Bonferroni step-down correction, *p* value cutoff = 0.05, tree interval 3–5, kappa score = 0.5) was generated for dysregulated proteins and phosphoproteins in the cerebellum and cerebral cortex with ClueGO (Cytoscape) using organic layout. GO terms and pathways were arranged into three major groups described in chapters: “Disturbed mechanism of modulation of protein levels and DNA damage,” “Disturbed formation of neuronal cellular structures: organelles and macromolecules,” and “Neuronal cell functionality affected by processes following perturbed cytoskeletal (microtubule) complex formation.” Proteins were assigned to GO terms and pathways based on ClueGO analysis (genes with corresponding functions). The same analysis, however, with no arrangement is included as Supplementary Fig. 3 in order to enable free interpretation of data. The lists of GO terms and pathways with *p* values are included in Tables 7, 8, 9, 10, 11, and 12 and Supplementary Tables 3–5

Based on the CPDB and ClueGO analysis (GO term analysis (B, MF, level 5) and pathway enrichment), we have selected three groups of biological processes, which are enriched among dysregulated proteins in both the cerebral cortex and cerebellum. The complete lists of GO terms and pathways related to dysregulated proteins and phosphoproteins are included in Supplementary Tables 3–5. Of note, results obtained with both bioinformatic tools (CPDB and ClueGO) showed overlap in the majority of biological processes and pathways (see Supplementary Tables 3–5). The analysis is visualized in Fig. 9 where the processes were groped arbitrarily and in Supplementary Figs. 3 and 4 with no arbitrary grouping. Selection of the groups was performed according to mutual relations and similarity of biological function of pathways and GO terms. We termed the first group “Disturbed mechanisms of modulation of protein levels and DNA damage” associated with either protein ubiquitination, translation initiation, splicing, or chromatin organization (Tables 7 and 8; Fig. 9, group I). The second group consists of biological processes that likely result from the initial dysregulations in the first group. The second group was termed “Disturbed formation of neuronal cellular structures: organelles and macromolecules” and is related to aberrant protein folding and affected organelle biogenesis and maintenance, which includes microtubule and actin cytoskeleton organization, the formation of axons and dendrites, axon guidance, the formation of gap junctions, and cellular vesicles (Tables 9 and 10; Fig. 9, group II). The third group was termed “Neuronal cell functionality affected by processes following perturbed cytoskeletal complex formation and apoptosis” (Tables 11 and 12; Fig. 9, group III). The processes affected in this group consist of axonal transport along microtubules, including synaptic vesicles and mitochondria, and consequently, mitochondrial respiratory chain complex formation and integration of energy metabolism (related to trans-synaptic signaling). Of note, several proteins participating in the processes belonging to this group were previously associated with other neurodegenerative disorders like HD, PD, and AD (Supplementary Tables 3–4). In this group, we also included programmed cell death as the final effect of all affected processes.

**Table 7:** Summary of GO terms and pathways containing three top dysregulated proteins and phosphoproteins (italics) from the cerebellum from group 1 (fold changes, *p* values, and GO terms)

Category	GO term/pathway	Source	Pathway or term *p* value/*q* value * or Bonferroni *p* value#	3 top dysregulated proteins	Log2 FC *p* value
DNA damage	Recognition of DNA damage by PCNA-containing replication complex	Reactome R-MMU:110314	0/0.02#	(Protiens) Ddb1 Ppp5c Rps27a	1.7/0.0349 0.5/0.0202 − 0.4/0.031
	Recruitment and ATM-mediated phosphorylation of repair and signaling proteins at DNA double strand breaks	Reactome R-MMU:5693565	0.0024/0.0472#		
	Global genome nucleotide excision repair (GG-NER)	Reactome R-MMU:5696399	0.0046/0.0421#		
	Based on [31]			(Protiens) Nedd Plec	1.6/0.0144 0.3/0.0349
	DNA damage/telomere stress-induced senescence	Reactome R-HSA-2559586	0.0082/0.0433*	(Phosphoproteins) *Hist1h1a* *Hist1h1d*	*− 1.4*/*0.0241* *− 1.0*/*0.0382*
	Based on [31, 32]			*Sirt2* *Acin1* *Plec*	*− 2.3*/*0.0262* *− 2.0*/*0.018* *− 1.4*/*0.0343*
Regulation of chromatin remodeling and transcription	Euchromatin	GO:0000791	0.0072/0.0315*	(Protiens) Trim28 Hist1h1d	1.0/0.0027 − 1.0/0.0418
	RNA polymerase II transcription cofactor activity	GO:0001104	0.0076/0.0296*	(Phosphoproteins) *Sirt2* *Hist1h1d Npm1*	*− 2.3*/*0.0262* *− 1.4*/*0.0382* *− 1.3*/*0.0209*
	Euchromatin	GO:0000791	0.0002/0.0031*		
	Heterochromatin	GO:0000792	0.0027/0.0140*		
Ubiquitination	Protein ubiquitination	Reactome R-MMU:8852135	0.0001/0.0135	(Proteins) Ddb1 Nedd8 Kras	1.7/0.0349 1.6/0.0144 1.4/0.0007
	Synthesis of active ubiquitin: roles of E1 and E2 enzymes	Reactome R-MMU:8866652	1.92E-05/0.0022		
	Parkin–ubiquitin proteasomal system pathway	WikiPathways WP2359	0.0081/0.0264

**Table 8:** Summary of GO terms and pathways containing three top dysregulated proteins and phosphoproteins (italics) from cerebral cortex from group 1 (fold changes, *p* values, and GO terms)

Category	GO term/pathway	Source	Pathway or term *p* value/*q* value* or Bonferroni *p* value#	3 top dysregulated proteins	Log2 FC/*p* value
DNA damage	DNA damage response, signal transduction by p53 class mediator	GO:0030330	0.0014/0.0205#	(Proteins) Gja1 Ndrg1 Hist1h1d	2.3/0.0286 1.8/0.0167 − 1.0/0.0006
	DNA damage/telomere stress-induced senescence	Reactome R-HSA-2559586	0.0017/0.0120*		
	Activation of DNA fragmentation factor	Reactome R-HSA-211227	0.0001/0.0027*		
Based on [31, 32, 33]			–	(Phosphoproteins) *Sirt2* *Bclaf1* *Plec*	*− 3.9*/*0.0091* *− 3.2*/*0.0226* *− 2.5*/*0.0126*
Translation	Formation of translation initiation complexes containing mRNA that does not circularize	Reactome R-MMU:157849	0.0013/0.0222#	(Protiens) Pabpc1 Eif3f Eif5a	2.0/0.0274 1.2/0.0091 1.2/0.0488
	Ribosome assembly	GO:0042255	0.0085/0.0501*		
	Cap-dependent translation initiation	Reactome R-HSA-72737	1.82E-05/0.0009*		
Protein folding	Chaperonin-mediated protein folding	Reactome R-MMU:390466	5.5E-05/0.0039#	(Protiens) Tubb3 Tcp1 Gnai2	− 0.9/0.0057 − 0.5/0.0155 − 0.4/0.0411
	Protein folding	Reactome R-MMU:391251	5.5E-05/0.0039#		
	Cooperation of Prefoldin and TriC/CCT in actin and tubulin folding	Reactome R-HSA-389958	0.0001/0.0027*		
	Cooperation of PDCL (PhLP1) and TRiC/CCT in G protein beta folding	Reactome R-HSA-6814122	0.0078/0.0356*	(Phosphoproteins) *Arfgef2* *Gng3* *Rgs7*	*− 4.5*/*0.0004* *− 3.6*/*0.0162* *− 2.8*/*0.0093*
	Chaperonin-mediated protein folding	Reactome R-HSA-390466	0.0089/0.0386*

**Table 9:** The summary of GO terms and pathways containing 3 top dysregulated proteins and phosphoproteins (italics) from cerebellum from Group2 (fold changes, *p* values, and GO terms)

Category	GO term/pathway	Source	Pathway or term *p* value/*q* value* or Bonferroni *p* value#	3 top dysregulated proteins	Log2 FC/*p* value
Microtubule cytoskeleton	KIF7 is recruited to the ciliary tip through interaction with axonemal microtubules	Reactome R-MMU:5610733	6.54E-07/8.89E-05#	(Protiens) Tuba1a Baiap2 Dync1i1	3.5/0.0276 1.0/0.0126 1.0/0.038
	Microtubule	GO:0005874	5.16E-06/0.0001*		
	Association of NuMA with microtubules	Reactome R-MMU:380316	4.97E-05/0.0047#		
	Regulation of microtubule cytoskeleton organization	GO:0070507	4.73E-05/0.001*	(Phosphoproteins) *Pak1* *Sirt2* *Klc1*	*− 2.5*/*0.0273* *− 2.3*/*0.0262* *− 1.8*/*0.0398*
	Microtubule binding	GO:0008017	0.0036/0.0217*		
	Regulation of microtubule cytoskeleton	WikiPathways WP2038	0.0014/0.0214*		
Actin cytoskeleton	Regulation of actin dynamics for phagocytic cup formation	Reactome R-MMU:2029482	0.0027/0.0470#	(Proteins) Kras Actn4 Baiap2	1.4/0.0007 1.3/0.0332 1.0/0.0126
	SNX9 recruits components of the actin polymerizing machinery	Reactome R-MMU:8868230	0.0011/0.0438#		
	Regulation of actin cytoskeleton—*Homo sapiens* (human)	KEGG:04810	0.0064/0.0245*		
	Actin filament bundle organization	GO:0061572	0.0024/0.0144*	(Phosphoproteins) *Numa1* *Sept4* *Rims1*	*− 1.6*/*0.0052* *− 1.5*/*0.0071* *− 1*/*0.0269*
Axon	Axon guidance	KEGG:04360	3.52E-05/0.0037#	(Proteins) Tuba1a rab5a Vps35	3.5/0.0276 1.7/0.0149 1.4/0.0333
	Neuron projection organization	GO:0106027	0.0002/0.0039*		
	Neuron projection development	GO:0031175	0.0012/0.0112*		
	Neuron projection development	GO:0031175	1.30E-09/2.76E-07*	(Phosphoproteins) *Map2* *Pak1* *Gprin1*	*− 2.8*/*0.0134* *− 2.5*/*0.0273* *− 2.2*/*0.0029*
	Regulation of cell projection organization	GO:0031344	2.42E-05/0.0007*		
	Axon guidance	Reactome R-HSA-422475	0.0017/0.0214*		
Dendrites	Dendrite development	GO:0016358	9.36E-07/3.37E-05#	(Phosphoproteins) *Map2* *Pak1* *Rtn4*	*− 2.8*/*0.0134* *− 2.5*/*0.0273* *− 2.0*/*0.0003*
	Dendrite morphogenesis	GO:0048813	0.0004/0.0068#		
	Regulation of dendrite extension	GO:1903859	0.0031/0.0162*		
Gap junction	Gap junction	KEGG:04540	1.48E-08/2.24E-06#	(Proteins) Tuba1a Kras Itpr3	3.5/0.0276 1.4/0.0007 1.3/0.023
	Gap junction—*Homo sapiens* (human)	KEGG:04540	0.0001/0.0033*		
Apoptosis	Apoptosis	Reactome R-MMU:109581	5.74E-07/7.87E-05#	(Proteins) Fam162a Vps35 Hist1h1d	1.6/0.0268 1.4/0.0333 − 1.0/0.0418
	Cell death signaling via NRAGE, NRIF and NADE	Reactome R-MMU:204998	0.0003/0.0231#		
	Apoptotic execution phase	Reactome R-MMU:75153	0.0014/0.0445#		
	Apoptotic execution phase	Reactome R-HSA-75153	5.20E-06/0.0008*	(Phosphoproteins) *Acin1* *Tjp2* *Hist1h1a*	*− 2.0*/*0.018* *− 1.9*/*0.0001* *− 1.4*/*0.0241*
	Apoptosis-induced DNA fragmentation	Reactome R-HSA-140342	0.0017/0.0214*		
	Apoptosis	Reactome R-HSA-109581	0.0002/0.0086*

**Table 10:** Summary of GO terms and pathways containing three top dysregulated proteins and phosphoproteins (italics) from the cerebral cortex from group 2 (fold changes, *p* values, and GO terms)

Category	GO term/pathway	Source	Pathway or term *p* value/*q* value* or Bonferroni *p* value #	3 top dysregulated proteins	Log2 FC/*p* value
Microtubule cytoskeleton	Kinetochore capture of astral microtubules	Reactome R-MMU:5666169	0.0062/0.0187#	(Proteins) Sept4 Ndrg1 Dync1i1	2.1/0.0011 1.8/0.0167 − 1.0/0.0023
	Microtubule cytoskeleton	GO:0015630	2.46E-05/0.0002*		
	Association of NuMA with microtubules	Reactome R-MMU:380316	0.0002/0.0115#		
	Organelle transport along microtubule	GO:0072384	1.92E-08/2.66E-06#	(Phosphoproteins) *Map2* *Nefh* *Sgip1*	*− 7.0*/*4.22E-05* *− 5.1*/*0.0002* *− 4.9*/*0.0005*
	Microtubule binding	GO:0008017	1.16E-11/8.48E-10*		
	Regulation of microtubule cytoskeleton	WikiPathways WP2038	1.67E-06/8.08E-05*		
Actin cytoskeleton	Regulation of actin dynamics for phagocytic cup formation	Reactome R-MMU:2029482	0.0007/0.0168*	(Proteins) Capn2 Slc9a3r1 actn1	1.6/0.0028 − 1.0/3.67E-05 0.6/0.0124
	Actin cytoskeleton	GO:0015629	0.0016/0.0043*		
	Y branching of actin filaments	BioCarta actinYPathway	0.0003/0.0042*		
	Actin-based cell projection	GO:0098858	8.95E-09/1.34E-06#	(Phosphoproteins) *Map2* *Bsn* *Nefh*	*− 7.0*/*4.22E-05* *− 5.6*/*0.0006* *− 5.1*/*0.0002*
	Actin cytoskeleton	GO:0015629	1.70E-11/5.32E-10*		
	Regulation of actin cytoskeleton	WikiPathways WP51	0.0004/0.0056*		
Axon	Axon guidance	KEGG:04360	0.0003/0.0125#	(Proteins) Gja1 Sept4 Slc9a3r1	2.3/0.0286 − 2.1/0.0011 − 1.0/3.67E-05
	Neuron projection development	GO:0031175	0.00049/0.0078*		
	Sema3A PAK-dependent axon repulsion	Reactome R-HSA-399954	0.0083/0.0292*		
	Axon guidance	KEGG:04360	0.0014/0.0070#	(Phosphoproteins) *Map2* *Bsn* *Nefh*	*− 7.0*/*4.22E-05* *− 5.6*/*0.0006* *− 5.1*/*0.0002*
	Neuron projection development	GO:0031175	8.26E-34/4.81E-31*		
	Axonal growth inhibition (RHOA activation)	Reactome R-HSA-193634	0.0087/0.0381*		
Dendrites	Dendrite development	GO:0016358	6.66E-13/1.33E-10#	(Phosphoproteins) *Map2* *Bsn* *Atcay*	*− 7.0*/*4.22E-05* *− 5.6*/*0.0006* *− 4.7*/*0.0287*
	Dendrite morphogenesis	GO:0048813	5.07E-10/8.32E-08#		
	Regulation of dendrite development	GO:0050773	0.0001/0.0053#		
Gap junction	Gap junction	KEGG:04540	4.72E-05/0.0036#	(Proteins) Gja1 Tubb3 Plcb1	2.3/0.0286 − 0.9/0.0057 0.5/0.0383
	Gap junction—*Homo sapiens* (human)	KEGG:04540	0.001/0.0082*		
	Gap junction—*Homo sapiens* (human)	KEGG:04540	0.0002/0.0033*	(Phosphoproteins) *Src* *Itpr1* *Prkcg*	*− 3.4*/*0.0001* *− 2.8*/*0.0001* *2.5*/*0.0225*
Apoptosis	Apoptosis	Reactome R-MMU:109581	0.0055/0.0335#	(Proteins) Gja1 Hist1h1d Tubb3	2.3/0.0286 − 1.0/0.0006 − 0.9/0.0057
	Apoptosis induced DNA fragmentation	Reactome R-HSA-140342	0.0001/0.0027*		
	Programmed cell death	Reactome R-HSA-5357801	0.0006/0.0063*		
	Regulation of neuron apoptotic process	GO:0043523	0.0005/0.0016#	(Phosphoproteins) *Add1* *Kcnma1* *Sirt2*	*− 4.9*/*0.0159* *− 4.1*/*0.002* *− 3.9*/*0.0091*
	Programmed cell death	Reactome R-HSA-5357801	7.85E-05/0.0016*		
	Regulation of neuron death	GO:1901214	3.34E-05/0.0017*

**Table 11:** Summary of GO terms and pathways containing three top dysregulated proteins and phosphoproteins (italics) from the cerebellum from group 3 (fold changes, *p* values, and GO terms)

Category	GO term/pathway	Source	Pathway or term *p* value/*q* value* or Bonferroni *p* value#	3 top dysregulated proteins	Log2 FC/*p* value
Synaptic vesicle	Synaptic vesicle cycle	KEGG:04721	0.0003/0.0201#	(Proteins) Rab5a Syp Arpc3	1.7/0.0149 − 0.9/0.025 − 0.8/0.0435
	Regulation of synaptic vesicle cycle	GO:0098693	0.0006/0.0095*		
	Synaptic vesicle pathway	WikiPathways WP2267	9.79E-06/0.0003*		
	Regulation of synaptic vesicle exocytosis	GO:2000300	2.28E-06/0.0001*	(Phosphoproteins) *Syn1* *Slc32a1* *Braf*	*− 2.4*/*0.0245* *− 1.7*/*0.0031* *− 1.5*/*0.0294*
	Regulation of synaptic vesicle cycle	GO:0098693	2.12E-05/0.0007*		
	Synaptic vesicle pathway	WikiPathways WP2267	0.0001/0.0061*		
Trans-synaptic signaling and neurotransmitters	Anterograde trans-synaptic signaling	GO:0098916	7.04E-05/0.0025*	(Proteins) Rab5a Vps35 Kras	1.7/0.0149 1.4/0.0333 1.4/0.0007
	Dopamine receptor binding	GO:0050780	2.18E-05/0.0002*		
	Glutamate neurotransmitter release cycle	Reactome R-HSA-210500	0.008/0.0264*		
	Gamma-aminobutyric acid receptor life cycle pathway	BioCarta gabaPathway	0.0031/0.0141*		
	Anterograde trans-synaptic signaling	GO:0098916	4.25E-05/0.001*	(Phosphoproteins) *Map2* *Pak1* *Syn1*	*− 2.8*/*0.0134* *− 2.5*/*0.0273* *− 2.4*/*0.0245*
	Glutamatergic synapse	KEGG:04724	0.0018/0.0181#		
	GABA synthesis, release, reuptake, and degradation	Reactome R-HSA-888590	0.0038/0.028*		
	Serotonin neurotransmitter release cycle	Reactome R-HSA-181429	0.0034/0.028*		
Glycolysis	Glycolysis/gluconeogenesis	KEGG:00010	0.0004/0.0247#	(Proteins) Mpc1 Aldoa Akr1a1	1.4/0.0433 0.5/0.0064 0.5/0.0422
	Glycolysis	Reactome R-MMU:70171	0.0039/0.043#		
	Glycolysis and gluconeogenesis	WikiPathways WP:157	0.0023/0.047#		
TCA and oxidative phosphorylation	The citric acid (TCA) cycle and respiratory electron transport	Reactome R-MMU:1428517	2.33E-10/3.66E-08#	(Proteins) Uqcrh Mpc1 Etfdh	3.4/0.0134 1.4/0.0433 1.4/0.0144
	Oxidative phosphorylation	KEGG:00190	3.18E-08/4.74E-06#		
	Mitochondrial electron transport, NADH to ubiquinone	GO:0006120	0.0011/0.0112*		
Signaling pathways	EGF–EGFR signaling pathway	WikiPathways WP437	3.56E-05/0.0011*	(Proteins) Rab5a Nedd8 Kras	1.7/0.0149 1.6/0.0144 1.4/0.0007
	ErbB1 downstream signaling	PID	1.40E-07/5.61E-05*		
	PDGFR-beta signaling pathway	PID	5.32E-07/7.21E-05*		
	EGFR1	NetPath	0.0015/0.0214*	(Phosphoproteins) *Map2* *Pak1* *Tjp2*	*− 2.8*/*0.0134* *− 2.5*/*0.0273* *− 1.9*/*0.0001*
	Ephrin signaling	Reactome R-HSA-3928664	0.0038/0.028*		
	MAPK cascade	WikiPathways WP422	0.0087/0.0450*

**Table 12:** Summary of GO terms and pathways containing three top dysregulated proteins and phosphoproteins (italics) from the cerebral cortex from group3 (fold changes, *p* values, and GO terms)

Category	GO term/pathway	Source	Pathway or term *p* value/*q* value* or Bonferroni *p* value#	3 top dysregulated proteins	Log2 FC/*p* value	
Synaptic vesicle	Synaptic vesicle cycle	KEGG:04721	0.0001/0.0063#	(Proteins) Rab1b Rph3a Stx1b	− 1.5/0.0006 − 1.1/0.0004 − 0.5/0.0157	
	Establishment of synaptic vesicle localization	GO:0097480	0.0001/0.0032*			
	Synaptic vesicle exocytosis	GO:0016079	0.0002/0.0103#			
	Synaptic vesicle cycle	GO:0099504	5.25E-12/1.01E-09#	(Phosphoproteins) *Rims* *Pacsin1* *Sgip1*	*− 5.0*/*0.0003* *− 4.9*/*0.0027* *− 4.9*/*0.0349*	
	Synaptic vesicle pathway	WikiPathways WP2267	3.24E-07/2.48E-05*			
	Synaptic vesicle localization	GO:0097479	3.79E-13/7.65E-11#			
Trans-synaptic signaling and neurotransmitters	Anterograde trans-synaptic signaling	GO:0098916	9.92E-06/0.0003*	(Proteins) Gad1 Cspg5 Tspan7	2.1/0.015 − 1.6/0.0367 1.5/0.0044	
	Dopaminergic synapse	KEGG:04728	8.01E-06/0.0007#			
	Opioid signaling	Reactome R-MMU:111885	5.33E-05/0.0038#			
	Glutamate binding, activation of AMPA receptors, and synaptic plasticity	Reactome R-HSA-399721	0.0024/0.0139*			
	Anterograde trans-synaptic signaling	GO:0098916	6.42E-24/1.25E-21*	(Phosphoproteins) *Map2* *Bsn* *Rims*	*− 7.0*/*4.22E-05* *− 5.6*/*0.0006* *− 5.0*/*0.0003*	
	Glutamatergic synapse	KEGG:04724	1.56E-07/1.86E-05#			
	GABAergic synapse	KEGG:04727	4.44E-06/0.0003#			
	Dopaminergic synapse	KEGG:04728	3.42E-05/0.0017#			
Glycolysis	Glucose-6-phosphate dehydrogenase deficiency	SMPDB SMP00518	0.0063/0.024*	(Proteins) Pfkl Taldo1	1.0/0.033 − 0.4/0.009	
	Glycolysis	HumanCyc PWY66-400	0.0093/0.0398*	(Phosphoproteins) *Aldoc* *Eno1* *Pgk1*	*− 3.2*/*5.81E-05* *− 2.5*/*0.013* *− 1.9*/*0.0454*	
TCA and oxidative phosphorylation	The citric acid (TCA) cycle and respiratory electron transport	Reactome R-MMU:1428517	0.0008/0.0179#	(Proteins) Mt-Co3 Ndufc2 Chchd6	− 1.8/0.0494 1.6/0.0333 − 1.3/0.0258	
	Oxidative phosphorylation	KEGG:00190	7.08E-08/8.85E-06#			
	Mitochondrial respiratory chain	GO:0005746	1.58E-05/0.00139#			
Signaling pathways	EGFR1	NetPath	0.0019/0.0126*	(Proteins) Gja1 Cstb Capn2	2.3/0.0286 2.0/0.0471 1.6/0.0028	
	ErbB1 downstream signaling	PID	0.0003/0.0038*			
	PDGFR-beta signaling pathway	PID	0.0008/0.0072*			
	EGFR1	NetPath	3.28E-06/0.00012*	(Phosphoproteins) *Sptbn1* *Epn2* *Rras2*	*− 4.7*/*0.0159* *− 4.3*/*0.0032* *− 3.8*/*0.0019*	
	VEGF	INOH	1.88E-06/8.66E-05*			
	MAPK signaling pathway	KEGG:04010	0.0003/0.0074#			

### Disturbed Mechanism of Modulation of Protein Levels and DNA Damage (Group 1)

In the cerebellum (Table 7; Fig. 9, I; Supplementary Tables 3–5), several dysregulated proteins are involved in joining and releasing of ribosomal subunits and hydrolysis of mRNA with premature codon termination (Ppp2r1a (↑), Rplp2 (↑), Rps27a (↓); (↑) upregulated in Ki91 mouse; (↓) downregulated in Ki91 mouse; (p) phosphoproteins). Several proteins with altered levels of phosphorylation are related to such GO terms as nucleosome assembly (Hist1h1a (↓), HIst1h1d (t↓; p↓), Hist1h4a (↓)) or chromatin organization (Tom1l2 (↓), Trim28 (↓), Numa1 (↓)). Coherently, the total level of chromatin remodeling factor *Rbbp4* (↑) is markedly increased. Signs of dysregulated mRNA splicing are suggested by dysregulation of several proteins at the level of phosphoprotein isoforms (Acin1 (p↓), Npm1 (p↓), Srsf9 (p↓), and Thrap3 (p↓)) and dysregulated proteins (Hnrnpk (↑), Ddx5 (↑), Snrnp200 (↑)), highly upregulated Hnrnph2 (↑), and downregulated Srsf1 (↓)). Aberrations regarding mRNA processing may be linked to GO terms implicating DNA damage: DNA repair and global genome nucleotide excision repair (*GG-NER*), which involve Ppp5c (↑), and highly upregulated Ddb1 (↑). In turn, Uba1 (↑), Uba52 (↓), and Usp9x (↓) are taking part in ubiquitin activation, whereas highly elevated *Rab5a* (↑) and Vps35 (↑) have a role in the maturation of macroautophagosomes.

Compared to the cerebellum, in the cerebral cortex (Table 8; Fig. 9, I; Supplementary Tables 3–5), we have also identified similar processes involved in modulation of the protein level. These processes were predominantly represented by proteins related to translation initiation, including translational factors (Eef1b2, Eef1d, Eif3f, Eif5a, Pabpc1) belonging to the list of top dysregulated proteins (all (↑); log2 fold change ≤ − 1.0) and ribosomal proteins responsible for joining and releasing ribosomal subunits and premature termination of translation (Rpl13, Rplp0, Rps13, Rps27, Rps5; all (↑)), which interestingly also are all being upregulated. Similar to the cerebellum, there were several histone proteins with altered levels (Hist1h1b, Hist1h1d, H2af2 (all ↓)). Among proteins with altered level of phosphorylation were those which are implicated in forming nucleosomes or remodeling chromatin (Hdac2, Nap1l4, all (p↓)), transcription (Sltm, Trim28, all (p↓)) or translation (Eif4g3, Eif3c, all (p↓)), and ubiquitination (Uba1, Usp10, all p↓). Several proteins with more than one site of altered phosphorylation were related to splicing (Srsf9, Prpf4b, Zranb2, Rbm39, Srrm1, Srrm2, all (p↓)), and among the top dysregulated proteins, there was highly downregulated Sf1 protein (log2 FC = − 2.08).

### Disturbed Formation of Neuronal Cellular Structures: Organelles and Macromolecules (Group 2)

The second group of molecules with altered levels indicates aberrant turnover of cellular microtubule cytoskeleton which in turn may disturb various cell structures in neuronal cells. In the cerebellum (Table 9; Fig. 9, II; Supplementary Tables 3–5), proteins required for interphase microtubule organization from centrosome are upregulated (Ppp2r1a, Tuba1a, Tubb4a, Tubb5, Ywhae (all ↑)) together with several other microtubule proteins (DynII2, Dynlrb1, Tuba3a, Tubb3 (all ↑)). Remarkably, Tuba1/Tuba3a is a top upregulated protein in the cerebellum with log2 FC 3.51 (Supplementary Table 6). Along this line, neuron projection organization might be affected, which manifests through elevated levels of Cfl1, Dpysl2, Gnai2, and Kras (all ↑) and decreased levels of Rac1–3, Atp1a3, and Vps35 (all ↓), and Baiap2 (↑)) (Table 9; Fig. 9, II; Supplementary Tables 3–5). A similar subset of proteins (Gnai2, Itpr3, Kras, Tuba3a, Tubb3, Tubb4a, Tubb5 all (↑) is also responsible for the exchange of small molecules through gap junctions. Among dysregulated proteins are also Rho GTPase effectors, which are important modifiers of microtubule and actin dynamics (Baiap2, Rac1, Dync1i1 (all ↑)). Proteins of NGF and EGFR1 signaling pathways are mostly elevated. *NGF signaling* included Baiap2 (↑), Cltc (↑), Dnm1 (↑), Dpysl2 (↑), Kras (↑), Nedd8 (↑), Ppp2r1a (↑), Rab5a (↑), Rac1 (↑), Arpc3 (↓), Rps27a (↓), and Ywhae (↓), while *EGFR1 signaling* included Actn4 (↑), Atp6v1c1 (↑), Cltc (↑), Dnm1 (↑), Dpysl2 (↑), Dync1i1 (↑), Kras (↑), Ndufa13 (↑), Plec (↑), Rab5a (↑), Rac1 (↑), Stxbp1 (↑), and Syp (↓) (Table 9; Fig. 9, II; Supplementary Table 4). Likewise, proteins which have biological function in processes related to microtubule formation demonstrate decreased phosphorylated isoforms such as phosphoproteins involved in axonogenesis (Braf, Cttn, Map1b, Map2, Mapt, Pak1, Rtn4 (all p↓)) and dendrite development (Ctnnd2, Farp1, Git1, Map1b, Map2, Mapk8, Mink1, Pak1, Palm, Slc12a5 (all p↓)) (Table 9; Fig. 9, II; Supplementary Tables 3–5). Axon guidance is one of the two pathways containing the highest number of top decreased phosphoproteins (Ablim1, Dpysl4, Pak1, Braf, Ncam1, Dpysl3, Git1 (all ↓)) along with the EGFR pathway (Tjp2, Pak1, Braf, Plec, To1l2, Mink1, Git (all p↓)). Decreased levels of phosphorylation also encompass proteins associated with response to NGF (Braf, Ehd1, Kcnc1, Stmn2 (all p↓)).

In the cerebral cortex (Table 10; Fig. 9, II; Supplementary Tables 3–5), we also identified proteins comprising terms related to microtubule organization, which pertained to recruiting various proteins to the centrosome during its maturation (Actr1a (↓), Hsp90aa1 (↑), Tubb4a (↓), Tubb5 (↑), and Ywhag) and axon guidance (Camk2b (↑), Gnai2 (↑), Gsk3b (↓), Ppp3r1 (↑), Rac1–3 (↓), Pfn2 (↑), Arp3 (↓), Arpc4 (↑)). Furthermore, altered levels of proteins responsible for the binding vesicle to dynein and dynactin were detected (Uso1 (↑), Actr1a (↓), Copz1 (↑), Pafah1b2 (↑), Rab1b (↓)). There are much more prominent changes associated with microtubules in the phosphoproteome of the cortex. Those include tubulin binding (Brsk1, Kif3a, Kif5b, Lzts1, Map1a, Map1b, Map2, Map4, Map6d1, Mapt, Ndel1, Sgip1 (all p↓)), transport along microtubules (Kif3a, Kif5b, Klc1, Map1b, Mapt, Ndel1 (all p↓)), and axonogenesis (Bmpr2, Braf, Brsk1, Brks2, Cdh11, Crmp1, Dclk1, Gsk3b, Kif5b, Map1b, Map2, Mapt, Myh10, Ndel1, Pak1, Rufy3, Syngap1 (all p↓)) (Table 10; Fig. 9, II; Supplementary Tables 3–5). In total, 48 downregulated phosphoproteins play a role in neuron projection organization (among them, there are 22 proteins with log2 FC ≤ − 3.0), 20 in microtubule binding (9 proteins with log2 FC ≤ − 3.0) and 45 in the regulation of organelle organization (17 proteins with log2 FC ≤ − 3.0). Moreover, several phosphoproteins are related to negative regulation of protein complex assembly (Add1, Add2, Dmtn, Gsk3b, Sptbn1, Tfip11 (all p↓)).

### Neuronal Cell Functionality Affected by Processes Following Perturbed Cytoskeletal (Microtubule) Complex Formation (Group 3)

Transport along microtubules in the axon is essential for proper localization of organelle and particles in neurons. Since proteins of cytoskeleton-dependent transport are dysregulated in the cerebellum (Table 11; Fig. 9, III; Supplementary Tables 3–5), synaptic vesicle localization is likely to be affected. This is consistent with observed dysregulation of proteins involved in synaptic vesicle transport and transmission (Baiap2 (↑), Itpr3 (↑), Kras (↑), Rac1 (↑), Stxbp1 (↑), Tnr (↑), Syp (↓)) belonging to the list of top dysregulated proteins (Supplementary Table 6). Likewise, proteins with decreased levels of phosphorylation are implicated in neurotransmitter transport (Braf, Pak1, Pclo, Rims1, Slc32a1, Stx1b, Syn1, Syn2 (all p↓)). Notably, a group of proteins with highly dysregulated levels of phosphorylation has a main biological function related to trans-synaptic signaling (Braf, Ncam1, Slc3a1, Syn1, Bsn, Mink1, Slc32a1, Shank1, Shank2, Slc12a5 (all p↓)) (Table 11; Fig. 9, III; Supplementary Tables 3–6).

Disturbed transport along axons may also have an impact on the imbalance in the exchange between newly synthesized and used or damaged mitochondria in nerve cell ending. Several proteins with increased levels in the cerebellum (Table 11; Fig. 9, III; Supplementary Tables 3–5) are involved in glycolysis, gluconeogenesis, and citric acid cycle (Akr1a1, Aldoa, Gpi1, Cs, Cycs (all ↑)) as well as a large subset of proteins with higher fold increase involved in the respiratory electron transport chain (Atp5b, Atp5f1, Etfdh, Ndufa10, Ndufa13, Ndufs8, Sdhb, Uqcrh, Uqcrq (all ↑)). Interestingly, there is no GO term related to energy metabolism involving dysregulated phosphoproteins, neither for the cerebellum nor the cerebral cortex.

In the cerebral cortex (Table 12; Fig. 9, III; Supplementary Tables 3–5), probably also as a consequence of microtubule dysfunction, several biological processes may be perturbed such as synaptic vesicle cycle and exocytosis (Cadps (↓), Pfn2 (↑), Rph3a (↓), Stx1b (↓), Synj1 (↓), Atp6v1b2 (↑), Atp6v0d1 (↑), Atp6v1g2 (↑)). Moreover, proteins with decreased levels of phosphorylation are also involved in exocytosis of synaptic vesicles (Amph, Braf, Pclo, Rab3gap1, Rims1, Syn1 (all p↓)) and modulation of chemical synaptic transmission (Atp2b2, Braf, Ctnnd2, Kif5b, Lzts1, Pak1, Plcl1, Ppfia3, Rab3gap1, Rims1, Shank1, Shank2, Shisa7, Syn1, Syngap1, Synpo (all p↓)). In total, 55 phosphoproteins are involved in trans-synaptic signaling and 18 in synaptic vesicle localization. From these, 21 belong to the list of top dysregulated phosphoproteins with log2 FC < − 3.0 (Stx16, Src, Ank2, Tbc1d24, Gja1, Sgip1, Pacsin1, Klc1, Reps2, Sptbn1, Epn2, Eps15l1 (all p↓)) (Supplementary Table 6). Similar to the cerebellum, we observed dysregulation of many proteins involved in oxidative phosphorylation (Atp5d (↓), Cyc1 (↓), Ndufa4 (↑), Ndufb6 (↑), Ndufc2 (↑), Ndufs4 (↑), Uqcrb (↓)), and Cyb5a, Ndufb6, and Ndufc2 are highly upregulated, and at the top of it, Mtco1 has the highest level of log2 FC 2.95 in all dysregulated proteins in the cortex (Table 12; Fig. 9, III; Supplementary Tables 3–6).

Finally, altered levels of proteins in the cerebellum (Table 11, Fig. 9, III) suggest that pathway related to apoptosis is activated (Kpnb1 (↑), Plec (↑), Vim (↓)). Another GO term, activation of BH3-only proteins (DynII2 (↓), Ywhae (↓)), implies canonical mitochondrial apoptosis [34]. There are also cerebellar phosphoproteins related to apoptosis (Mapk10, Mapk8, Sept4, Tjp2, Hist1h1d1, Hist1h1a, Plec, Acin1 (all p↓)) and mitophagy (Atg4b, Cttn, Htt, Tom1l2, Vdac1 (all p↓)). In the cortex (Table 12; Fig. 9, III; Supplementary Tables 3–5), we found only a few proteins related to apoptosis (Kpnb1 (↑), Lmnb1 (↑), Ppp3r1 (↑), Ywhag (↑)). However, proteins from the list of top dysregulated proteins (Exoc1 (↑), Rab1b (↓), Vps26b (↑)) (Supplementary Table 6) are implicated in the regulation of macroautophagy.

### Subcellular Localization of Dysregulated Proteins

We performed analysis of “cellular component” GO terms (*p* value cutoff < 0.01) in CPDB to examine putative cellular localization of dysregulated proteins and phosphoproteins (Supplementary Fig. 4; Supplementary Table 7). The majority of the differentially expressed total proteins (Supplementary Fig. 4A, C) were assigned to localize in “extracellular exosome” (42% cerebellum and 46% cerebral cortex) or assigned as *cytoplasmic* (39% cerebellum and 44% cortex). A large number of dysregulated proteins were also identified as *mitochondrial* (28% cerebellum and 20% cortex) and *cytoskeletal* (26% cerebellum and 24% cortex). There were also proteins which were assigned to cellular structures which are typical for neurons, such as *dendrites* (14% cerebellum and 6% cortex) and *axons* (13% cerebellum and 8% cortex). In addition, in the cerebellar cortex, 9% of proteins are associated with *lytic vacuoles*.

Coherently with total proteome, proteins with altered level of phosphorylation (Supplementary Fig. 4B, D) were assigned as cytoplasmic (30% cerebellum and 35% cortex), cytoskeletal (27% cerebellum and 29% cortex), and secretory vesicles (7% cerebellum and 21% cortex). Respectively, 19% (cerebellum) and 17% (cortex) of proteins localized in dendrites and 9% (cerebellum) and 15% (cortex) were assigned for axons.

In apparent contrast to the total proteome, a prominent number of 26% of all phosphorylated proteins in the cerebellum (Supplementary Fig. 4B) were associated with the nucleus. In the cortex (Supplementary Fig. 4D), there were 22% of phosphoproteins localized in the nucleus and 4% associated with the cell membrane. Decreased phosphorylated proteins predicted to be localized in the nucleus included Sirt2, Srrm2, Thrap3, Acin1, Bclaf1, Ctr9, Rbm39, Numa1, Npm1, Trim28, Srsf9, Matr3, Sept4, Ndrg2, and Ppp6r3 (Supplementary Table 7). These nuclear proteins are implicated in cell cycle, DNA damage, and splicing; however, no GO terms containing dysregulated nuclear phosphoproteins were identified which relate to transcription factors or direct gene expression control. In coherence with the analysis of biological processes, we found no proteins with altered level of phosphorylation, which localized in the mitochondria, neither in the cerebellum nor in the cerebral cortex.

### Cellular Identity of Dysregulated Proteins

The analysis of cellular markers among dysregulated proteins and phosphoproteins was performed using the BrainMap tool of the *Allen Brain Atlas* for cerebral cortex (Supplementary Fig. 5A) and DropViz for cerebellum (Supplementary Fig. 5B). The analysis revealed a characteristic pattern of cellular markers of inhibitory neurons, which consisted of Gad1 (log2 FC change = 2.06, total), Pvalb (log2 FC = − 2.92, total), Aldoc (log2 FC = − 3.24, phospho), Chgb (log2 FC = − 4.2, phospho), and Fgf13 (log2 FC = − 0.86, phospho) in the cortex (Supplementary Fig. 5A). Another pair of markers, namely Pak1 (log2 FC = − 3.03, phospho) and Arhgap32 (log2 FC = − 3.48, phospho), was characteristic for excitatory neurons. The third group of markers consisted of dysregulated proteins which were characteristic for oligodendrocytes and their precursor cells. Phosphorylated Ermn (log2 FC = − 4.4, phospho) and Opalin (log2 FC = − 4.33, phospho) were highly expressed in mature oligodendrocytes, while Mbp (log2 FC = − 0.69, total; − 1.77, phospho), Bcas1 (log2 FC = − 3.68, phospho), and Plp1 (log2 FC = − 0.5, total) are markers of oligodendrocyte precursors and mature oligodendrocytes. Moreover, there was also one highly upregulated astrocytic protein—Gja1 (log2 FC = − 2.27, total; − 3.78, phospho) (Supplementary Table 6). In the cerebellum (Supplementary Fig. 5B), the analysis demonstrated several cellular markers with the ratio of relative expression between 3.14 and 63 (particular cell type vs other cell types). We found three proteins, which could be considered as markers of inhibitory neurons, namely Purkinje cells and pvalb-positive interneurons: Slc32a1 (log2 FC = − 1.66, phospho), Nefh (log2 FC = 0.4, total), and Baiap2 (log2 FC = 1.01, total). In addition, Itpr1 (log2 FC = − 1.21, phospho) is expressed strongly in Purkinje neurons, but not in other cell types. Furthermore, three proteins are markers for Bergmann glia and astrocytes: Slc1a3 (log2 FC = 0.35, total; − 1.36, phospho), Slc7a10 (log2 FC = − 1.15, phospho), and Dao (log2 FC = 0.74, total), and one protein specific for oligodendrocytes: Enpp6 (log2 FC = 0.65, total). Among proteins with altered level and assigned log fold change, we did not identify conclusive markers for microglia and endothelial cells. However, Hist1h1d (cerebellum log2 FC = − 0.96, total; 1.77, phospho; cortex = − 1.04, total), a microglia marker (with a low percentage of presence in microglia and no expression (0%) in any other cell types), is dysregulated in three analysis (total proteome and phosphoproteome of the cerebellum and total proteome of the cerebral cortex) (Supplementary Table 6). Altogether, proteomic changes occur mainly in neurons and oligodendrocytes in the cerebral cortex (Supplementary Fig. 5A), and inhibitory neurons, Bergmann glia, and astrocytes in the cerebellum (Supplementary Fig. 5B).

## Discussion

In the present work, we have defined the early molecular signs of SCA3/MJD polyQ neurodegenerative disease. We used a homozygous Ki91 mouse knock-in model, which mimics the genetics and pathological SCA3 situation in patients including presymptomatic phase and later disease onset [14]. Taking advantage of the model, we challenged the question of which molecular events take place before the disease outbreak in the brain by selecting a set of high-throughput methods to define the brain transcriptome first in 2-month-old and later at 10–14-month-old animals by qPCR. Subsequently, we identified the brain proteome and phosphoproteome in 2-month-old Ki91 animals. Our goal was to identify the molecular triggers of the disease which may be evident early in life and early in disease pathology and therefore are not “contaminated” by secondary molecular signs originating from neuronal dysfunction and death. In our work, we included the cerebellar cortex as an important source of early signs in SCA3 brain [35]. Recent findings also emphasize the role of communication between the cortex and cerebellum as one of the pathology hallmarks of SCA3 [36–39].

**Fig. 10:**
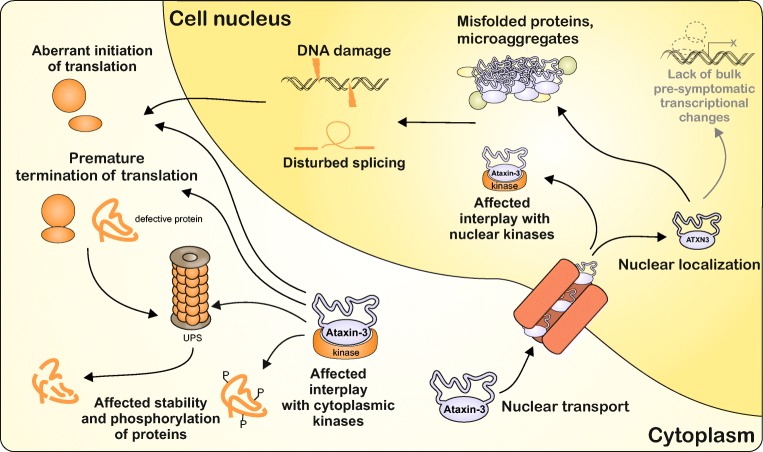
The diagram of processes affected by molecules identified in young Ki91 mouse brain during the early SCA3/MJD pathogenesis

The first essential finding is the sequence of general molecular events, which governs the disease onset process in SCA3/MJD. We found that Ki91 homozygous animals do not contain mutant ataxin-3-related transcriptional changes in cerebellum and cortex tissue at 2 months of age and do not show any behavioral changes at that stage; however, in the cerebellum and cortex, there are sparsely occurring cells with nuclear localization of mutant ataxin-3. At the same time, 2-month-old Ki91 animals already demonstrate prominent changes both at the level of total proteome and at the level of phosphoproteins. Importantly, the general level of phosphorylation of proteins is greatly decreased in our Ki91 SCA3/MJD mouse model. Of note, many of the changes exceed the decrease of log2 FC − 3 and lower. Furthermore, we demonstrate that later ages of 10–14-month-old Ki91 animals do contain transcriptional changes in both the cerebellum and cerebral cortex, and the mature postmitotic neural cultures from patient neurons from iPSC also contain some transcriptional changes identified in our 10–14-month-old Ki91 animals. The type of neural cultures that we used was reported to be positive for GABA and GAD67 and originate from cells that display differentiation profile toward hindbrain identity [24], whereas the mouse data are generated from the cortex and cerebellum. For example, Olig1 is elevated both in mouse tissue and MJD iPSC-derived neural cells, but there are a number of different cells (including different types of neurons) that evolve from OLIG-positive progenitors depending on the brain region. Thus, concordant expression changes might not necessarily argue for the same changes at the cellular level. Comparison of data from WT and Ki91 mouse hindbrain (pons, medulla) versus MJD iPSC-derived neural cells and isogenic controls requires further studies. Together, the findings demonstrate that the possible sequence of events that leads to full-featured brain disease is composed of the changes at the level of many important proteins and phosphoproteins in the initial absence of mRNA changes dependent on mutant ataxin-3 (depicted in Fig. 10). These early events may further lead to transcriptional changes at later ages. Therefore, transcriptional changes in SCA3 can be classified as secondary and more severe disease signs. Previous reports demonstrated that the SCA3 disease pathogenic process includes relatively direct transcriptomic changes by binding of mutant ataxin-3 to chromatin and transcription factors [40–42]. Considering widespread expression of ataxin-3 across cells and tissues of Ki91 mouse [14], such direct interaction could occur early and readily and evoke transcriptional changes. However, the scenario that one cannot exclude is the direct influence of ataxin-3 on transcriptional changes which may start only at certain cellular lineages in the brain and that the present tissue-based NGS resolution does not allow for identification of transcriptional changes restricted to the initially small number of cells. We performed the analysis of the dysregulated protein markers to identify types of cells relevant to pathogenesis in the brain. Based on the analysis, we conclude that one of the affected cell types is the parvalbumin-positive neurons. Such neurons are usually GABAergic interneurons in the cortex, Purkinje cells, and other GABAergic neurons of the cerebellum [43, 44]. Another prominently affected cell type was astrocytes in the cerebellum and, to a lesser extent, in the cerebral cortex. In addition, according to our analysis of cell types using BrainMap, oligodendrocytes and excitatory neurons are also affected in the cerebral cortex. Another important possibility for the lack of transcriptional changes at the presymptomatic stage is the direct or indirect ataxin-3 effect on transcriptional changes, which start during later adult life as a response to another factor such as aging, stress, DNA damage, or protein interactions. The transcriptomic changes in SCA3 were so far investigated in the symptomatic phase of the disease such as in patient blood or in older brain tissue collected from mouse models [45–48]. One of the interesting outcomes from our analysis of transcriptome in older Ki91 animals suggests that precursors of oligodendrocytes are upregulated. This may suggest that demyelination and myelin repair responses occur later in the disease course. Since neuronal activity promotes myelination [49], the start of demyelination in SCA3 may be the result of the collapse of neuronal and axonal function which occurs later in the disease course.

On the other hand, elevated Olig1 level suggests some developmental abnormalities in SCA3. One possibility could be an increase in the prolonged existence of the Olig1-positive progenitor pool, which could disrupt the lineage segregation process. Another part of the results which indicate a developmental component in SCA3 pathogenesis is a group of dysregulated proteins involved in such processes as axon guidance and dendrite development.

In view of the fact that we did not find transcriptional changes in the brain of young Ki91 SCA3/MJD mouse model, we reasoned that proteomic changes were the other type of general molecular events that may be disturbed during the disease onset and presymptomatic SCA3 phase. We found a number of proteins with an altered level indicating that the dysregulations in protein level occur earlier and with greater intensity as compared to transcriptional changes during the disease progression. The changes in protein level in the cell can be evoked by posttranslational modifications such as protein phosphorylation. We tested the global phosphorylation of proteins and we found numerous decreased phosphoproteins in the cerebellum and cortex of young Ki91 animals (most of them exceeded log FC − 2) even reaching − 7 in Ki91 animals versus WT animals in the cerebral cortex. In addition, several common proteins were identified for both total proteome and phosphoproteome.

Interestingly, there were only 1 upregulated versus 94 downregulated and 2 upregulated versus 479 downregulated among dysregulated phosphoproteins in the cerebellum and cortex, respectively. This suggests that a general downregulation process of phosphorylated proteins in the cortex and cerebellum may exist in SCA3/MJD at young presymptomatic stage. Of note, ataxin-3 was demonstrated to interact with several kinases such as casein kinase 2 beta (Ck2b), glycogen synthase kinase 3 beta (Gsk3b), checkpoint kinase 1 (Chk1), cyclin-dependent kinase 4 (Cdk4), ribosomal protein S6 kinase (RPS6KA1), MAP kinase interacting serine/threonine kinase 1 (MKNK1), and mitogen-activated protein kinase kinase kinase 1 (MAP3K1) [50–54]. Importantly, ataxin-3 is the interactor of the powerful inhibitor of kinases, cyclin-dependent kinase inhibitor 1A (p21) [54]. p21 is controlling a multitude of kinases including the Cdks, Camk2d, Cdc28, Ywhaq, Mapk8 (Jnk), Mapk14, Prkaca, Csnk2b, Map3k5, and another powerful inhibitor Cdkn1b (p27) (source: BIOGRID and references therein). In fact, after analyzing the phosphorylation motifs of downregulated phosphoproteins, we discovered that the majority of motifs are targets for Ck1 and 2, Pka, Cdks kinases, Camk2, Gsk3, and other kinases such as the genome stabilizer Chk1.

Commonly dysregulated kinase for both analyzed cerebral regions is p21-activated kinase 1 (Pak1) with highly downregulated levels of phosphorylation at Ser-223 (cerebellum) and Ser-174 (cortex). Phosphorylation of Ser-223 is required for Pak1 activation and is performed by casein kinase (Csnk2a1), a known interactor of ataxin-3 [52, 55]. In general, Pak1 is implicated in neurodegenerative disorders, such as Alzheimer disease (AD), Huntington disease (HD), and Parkinson disease [56, 57]. Previously, Pak1 was shown to promote polyQ aggregation by enhancing its toxicity like in cellular models of HD, and this process was rescued by Pak1 knockdown [58]. Conversely, cell death was observed when the activity of Pak1 was downregulated in the 6-OHDA rat model of Parkinson disease [56]. In addition, it was demonstrated that oxidative stress reduced the active, phosphorylated form of Pak1 in dopaminergic neurons, which directly led to a reduction of anti-apoptotic Bcl2 protein levels via the ubiquitin/proteasome pathway. Moreover, an upstream phosphatase of Pak1 modulating its activity is Pp2b-A (Ppp3ca), which is upregulated in the cortex of Ki91 mouse. In addition, a core regulator of phosphatases and also PP2A (Ppp2r1a) [59], phospho Arpp19/Ensa (S62/67) is downregulated in the cerebellum.

Further analysis of dysregulated substrates in the kinases network (Fig. 7) underlies MAPT and MAP2 interconnected with the highest number of kinases dysregulated as phosphoproteins or total proteins. Those proteins are important for proper formation and stability of microtubule complexes [94]. Interestingly, only normal but not mutant ataxin-3 was shown to interact with microtubules [60, 61]. Coherently, we identified a number of other dysregulated proteins and phosphoproteins localized to cytoskeleton or dysregulaed kinases with functions related to cytoskeleton such as Pak1, Mapk, Brsk1, and Brsk2 (axon guidance and neurite outgrowth) [62–65]; Src (cytoskeleton remodeling) [66]; and Gsk3b (tau phosphorylation, ataxin-3 is a substrate) [50, 67].

We have clustered the dysregulated proteins and phosphoproteins into three groups reflecting the cellular processes and mechanisms in which the proteins are involved. Group 1 comprises basic cellular processes such as translation and its possible premature termination, disturbed assembly of the ribosome, ubiquitination but also dysregulation of proteins that are characteristic for nucleus and take part in nuclear regulation such as nucleosomes forming, chromatin remodeling, splicing, DNA damage, and repair. Most likely, many of those initial processes result from cellular stress related to the excessive abundance of mutant ataxin-3 in the nucleus, which we demonstrate in young Ki91 animals. The transfer of mutated ataxin-3 into the nucleus could also be related to altered levels of Trim28 (log2 FC 0.94 in the cerebellum, log2 FC 1.59 in the cerebral cortex), which was shown to drive the nuclear accumulation of two distinct proteins: α-Syn and Tau implicated in Parkinson disease and Alzheimer disease, respectively [68]. Notably, 26% of dysregulated phosphoproteins in the cerebellum and 22% in the cerebral cortex were predicted to localize in the nucleus, having a role in cell cycle, DNA damage, and splicing.

DNA damage was already demonstrated in SCA3, which was linked to inactivation of polynucleotide kinase 3′-phosphatase (Pnkp) by ataxin-3, an important enzyme for repair of DNA damage [69, 70]. In our experiments, we detected upregulated damage-specific DNA binding protein 1 (Ddb1) in the cerebellar tissue of Ki91 mouse model (log2 FC 1.65). The Ddb1 together with Chk1 is an ataxin-3 interactor; however, mutant ataxin-3 does not impair the interaction with Chk1 [53].

A molecule which may also be involved in the regulation of the level of other proteins is Pabpc1 which is highly upregulated in the cerebral cortex (log2 FC 2.02). Its major function is its role in the regulation of translation initiation and mRNA stability and is part of “stress granules” [71, 72].

The second group of dysregulated proteins is related to altered turnover of cytoskeleton which influences neuronal cellular structures and organelles. It was shown that expression of expanded polyQ proteins (ataxin-1, ataxin-3, and huntingtin) substantially affects the dynamics of microtubule cytoskeleton, by nucleation of new microtubules and rebuilding microtubule rods in neurons of a Drosophila model [73]. The cytoskeleton dysregulation potentially disturbs cell division, regulation of growth, structure, and guidance of neuronal projections and gap junctional communication. However, a particularly important resulting process detected among the dysregulated proteins seems to be the transport along microtubules and axons. Moreover, it was demonstrated that aggregates of the mutated form of ataxin-3 form inclusions inside the axon, which might perturb axonal transport [7]. The aberrant transport process may lead to disturbed synaptic transmission and energy metabolism and ultimately cause cell dysfunction and cellular death, and proteins involved in those processes were clustered in group 3.

An important part of SCA3 pathogenesis comprises impairments of the mitochondria, which include altered localization of ataxin-3 inside the mitochondria, enhanced interaction of ataxin-3 82Q with mitochondrial proteins (Sdhb and Ndufa4 which are also dysregulated in our analysis), and mitochondrial DNA damage documented in SCA3 models [51, 61, 74–76]. In line with those aberrations, in young Ki91 animals, we observed a number of dysregulated mitochondrial proteins belonging to group III such as Mt-co1 and mt-Co3 (cortex) and Uqcrh (cerebellum). Strikingly, in the cerebellum, proteins related to the mitochondria are all upregulated, whereas in the cerebral cortex, GO terms and pathways related only to upregulated proteins are mainly associated with mitochondrial electron transport chain and metabolism. Interestingly, it was shown that mitochondrial precursor accumulation may cause cellular stress influencing translation machinery [77]. Remarkably, there are no GO terms or pathways for dysregulated phosphoproteins referring to the mitochondria, which suggest that phosphorylation is not the mechanism responsible for altered levels of mitochondrial proteins.

In summary, we propose that the key proteins responsible for early pathogenic changes are various kinases interacting with mutant ataxin-3 and proteins dysregulated at the level of total or phosphorylation levels also demonstrating high log2 FC in Ki91 mouse which are Pak1, Arpp19/Ensa, Darpp-32, Sirt2, Acin1, Ddb1, Ppp5c, Hdac2, Thrap3, Trim28, Hnrnph2, Rbbp4, Bclaf1, Nedd8, Pabpc1, and several ribosomal proteins and elongation and splicing factors.

## Conclusions

In the current study, we demonstrate that early transcriptional changes influenced by mutant ataxin-3 do not occur in presymptomatic Ki91 SCA3/MJD mouse model; however, we identify prominent dysregulation of protein levels and phosphorylation. Based on the identified proteins and phosphoproteins, we dissected a set of most early events including impaired phosphorylation of proteins indicating the influence of mutant ataxin-3 on many kinases, proteins involved in DNA damage, and mechanisms playing a role in translation initiation. Moreover, GO term and pathway analysis indicates that dysregulated proteins may further impair cellular pathways and processes such as autophagy, energy metabolism, and transport of molecules and vesicles along axons. Subsequently, this may lead to severe effects such as defective projections and synaptic transmission eventually leading to neuronal dysfunction, subsequent demyelination, and neurodegeneration. For each of the identified pathways, we propose key molecules which are affected by the change of levels in the cerebellum and cortex. Altogether, we conclude that early triggers of the disease act on the level of pathways and processes engaging proteins rather than the transcription stage, whereas late transcriptomic changes most likely result from degeneration of particular populations of cells in the brain such as GABAergic neurons and oligodendrocyte precursors. The relatively high number of dysregulated proteins and phosphoproteins in Ki91 SCA3/MJD mice indicates that there are important modifiers of disease and biomarkers or even target molecules for therapies among those proteins. The most prominent new candidate molecules are kinases, such as Pak1, which have a vast influence on distinct cellular processes. Therefore, a particularly promising area for further studies on disease mechanism and potential therapies in SCA3 is the proteome and phosphoproteome.

## Methods

### Animals

Maintaining and breeding were performed at standard conditions with an 18-/6-h light/dark cycle and water and food provided ad libitum. The animals were marked using numerical ear tags (National Band & Tag Company, Newport, USA). The animals were sacrificed according to AVMA Guidelines for the Euthanasia of Animals by placing them in the programmable CO_2_ chamber (Rothacher Medical, Heitenried, Switzerland). The stress level of the animals was minimized throughout all the procedures and animal handling. The animal experimentation and handling were approved and monitored by the Local Ethical Commission for Animal Experiments in Poznan. The Ki91 SCA3/MJD mouse model was bred on C57BL/6 mouse genetic background for 10 generations and the animals were further maintained on the C57BL/6 genetic background. The homozygous (mut/mut) transgenic animals both from Ki91 knock-in mouse were generated by breeding heterozygous (mut/wt) animals. Homozygous Ki91 animals contained between 98 and 132 CAG repeats on a single mutant ataxin-3 allele. For RNAseq, Ki91 animals contained 100–110 CAG repeats. For total proteomics, Ki91 mice contained 98–106 CAG and Ki91 mice for phosphoproteomics contained 98–123 CAGs in mutant Atxn3 gene. For behavioral studies, Ki91 mice contained 103–132 CAGs. The total number of 80 animals of various ages was used for collecting brain tissues and 36 animals at the age of 2 months were used for the behavioral experiments. The cortex and the cerebellum for proteomic and transcriptomic analysis were always collected from brain tissue of a typical experimental group consisting of four mutant mice versus four nontransgenic or C57BL/6 mice. For qPCR validation of RNAseq analysis, 2- and 4-month-old samples from the FVB mouse strain have been used as a second control since the FVB strain demonstrates the same SNP profile (MGI in chromosome 12 )(e.g., Atxn3 and Serpina3n locus) as the 129sv genetic background originating from stem cells to which the Ki91 transgene was introduced. Supplementary Table 1 summarizes the number of animals by strain, genotype, age, experiment type, and tissue.

### Behavioral Studies

Assessment of motor function was performed as previously described [14]. Tests included accelerating rotarod (4–40 rpm in 9.5 min), elevated beam walk (diameter of rods—35, 28, 21, 17, and 9 mm), and parallel rod floor test in which the number of foot slips and locomotor activity in the experimental cage were analyzed and measured during 10 min. Each test consisted of one training day (T) and three consecutive days of measurement. In addition, we also performed scoring tests designed for evaluation of ataxia phenotype in mouse models [78]. All mice were weighed during each testing session. Graphing and statistics were performed on PrismVR software (San Diego, CA, USA), using ANOVA with Bonferroni post hoc test.

### RNAseq Analysis

NGS QC Toolkit (v 2.2.3) [79] was used to generate quality metrics for assessment of fastq input files. We used the statistics generated by the NGS QC Toolkit and prepared charts (Supplementary Fig. 6) which show the average quality score per base positions for each library. Fastq files were then aligned to the GRCm38.91 reference genome using STAR software (v 2.5.3a) [80] with parameters suggested by the QoRTs software (v 1.3.0) [81]. The parameters are used by default for estimation of genes and exon hit counts. The alignment statistics are demonstrated on charts in Supplementary Fig. 6A where the percentage of unique and multiply aligned reads are shown. The data were analyzed using three different experimental approaches which included the alignment/quantification of transcripts by Star/Deseq and HISAT/StringTie/Ballgown [82, 83]. In brief, the differential gene expression was calculated using DESeq2 software (v 1.14.1) [84]. To countercheck the results, the second software pipeline, namely HISAT2 (v 2.0.5) → StringTie (v 1.3.1c) → Ballgown (v 2.6.0) [82, 83, 85] was used. The results from the HISAT2 pipeline were consistent with the primary analysis and are not shown in the results section. Additionally, JunctionSeq analysis has been applied in the identification of altered splicing variants [86].

### Quantitative Real-Time PCR

Reverse transcription was performed with 500 ng of RNA using Maxima H Minus Reverse Transcriptase according to the manufacturer’s protocol using random hexamers. qPCR was performed according to MIQE Guidelines where relative gene expression and splicing events were estimated using the ΔΔCt method with ActinB, Pgk1, and Tfrc as control [87]. qPCR reaction was carried out on the BioRad CFX96 thermocycler using 5x HOT FIREPol EvaGreen qPCR Mix Plus (Solis Biodyne, Tartu, Estonia) with the following parameters: 95 at 15 min and 45 cycles of (95 °C 15 min, 60 °C 1 min) using primers synthesized at IBB, PAS (oligo.pl). Statistical analysis between two groups of samples was performed using two-sample *t* test with *p* < 0.05 considered as significant.

### Western Blot Analysis

Cerebellum and cortex samples were harvested from six young 2-month-old homozygous Ki91 and six age-matched C57BL mice. Tissues were homogenized in a buffer containing 60 mM TRIS-base, 2% SDS, 10% sucrose, and 2 mM PMSF using a Bioprep-6 homogenizer (Allsheng, China), followed by bath sonication for 3 min repeated three times while cooling the tube on ice in between the sonication. The protein concentration was estimated using a Pierce BCA protein assay kit (Thermo Scientific, Rockford, IL, USA) according to the manufacturer’s instructions. For each analysis, 25 μg of total protein or 45 μg of total protein for analysis of phosphorylated proteins was diluted in a loading buffer containing 2-mercaptoethanol and boiled for 5 min. The proteins were separated by SDS–polyacrylamide gel electrophoresis (5% stacking/12 or 10% resolving gel), transferred to nitrocellulose, and stained with Ponceau S solution. The blots were blocked with 5% nonfat milk in PBS/0.05% Tween 20 (analysis of total proteins) or 5% BSA (GE Healthcare, South Logan, UT, USA) in PBS/0.05% Tween 20 (analysis of total phosphoproteins) for 1 h at RT and, subsequently, incubated for 24 h at 4 °C with the following primary antibodies: mouse anti-PABP1 (1:1000; Cell Signaling, Danvers, MA, USA), rabbit anti-MBP (1:1000; Cell Signaling, Danvers, MA, USA), rabbit anti-DDB-1 (1:1000; Cell Signaling, Danvers, MA, USA), rabbit anti-Phospho-DARPP32 (Ser97) (1:1000; Cell Signaling, Danvers, MA, USA), rabbit anti-Phospho-Tau (Ser416) (1:1000; Cell Signaling, Danvers, MA, USA), mouse anti-Beta III Tubulin (1:1000; Darmstadt, Germany, Merck), mouse anti-NEFH (1:2000; DSHB Hybridoma Product RT97; deposited to the DSHB by Wood, J. [88]), and mouse anti-α-ACTIN (1:1000; DSHB Hybridoma Product JLA20; deposited to the DSHB by Lin, J.J.-C. [89]). The blots were probed with the respective HRP-conjugated secondary antibody (anti-rabbit or anti-mouse, 1:2000; Jackson ImmunoResearch, Suffolk, UK). The immunoreaction was detected using the ECL substrate (ThermoFisher Scientific, Waltham, MA, USA).

### Protein Extraction Digestion and Enrichment for Proteomics

Mouse brain tissues were lysed in buffer containing 1 M triethylammonium bicarbonate (TEAB), 0.1% SDS, and 1 mM sodium orthovanadate (NaVO_3_) in 2-ml tubes with stainless steel beads (Retch, Germany) followed by automatic homogenization using a Mixer Mill MM400 (Retch, Germany). Subsequently, the material was subjected to a threefold cycle of freezing and thawing followed by bath sonication for 3-min repeated three times while cooling the tube on ice in between the sonication. Protein concentration in the clear lysate was estimated using Pierce BCA protein assay kit (Thermo Scientific, Rockford, lL, USA) according to the manufacturer’s instructions. Ten-microgram aliquots of proteins were diluted with 15 μl of 50 mM NH_4_HCO_3_ and reduced with 5.6 mM DTT for 5 min at 95 °C. Samples were then alkylated with 5 mM iodoacetamide for 20 min in the dark at RT. Subsequently, the proteins were digested with 0.2 μg of sequencing-grade trypsin (Promega) overnight at 37 °C. For labeled free quantitative proteomics, 10 μg of digested protein per sample was used for analyses on LC/MS. A similarly prepared set of samples was used for phosphoproteomics. For each sample, 300 μg of digested protein was used for phosphopeptide enrichment. Phosphorylated peptides were enriched using two different kits, one exploiting titanium dioxide (TiO_2_) spin tips and the other high-capacity Fe-NTA spin column (Thermo Scientific, Rockford, lL, USA). Elution fractions containing phosphopeptides were further desalted on a C18 column (J.T. Baker, Center Valley, PA) prior to mass spectrometry quantitative measurements. To perform a global analysis of phosphoproteomic changes, we have collected another set of cerebellum and cortex samples. The final analysis was performed on eight cerebella of four young 2-month-old homozygous Ki91 and four age-matched C57BL mice and six cerebral cortices of three young 2-month-old homozygous Ki91 and three age-matched C57BL mice. Collected tissues were further subjected to protein isolation, trypsin fragmentation, and phospho-enrichment procedure (see “Methods” section).

### Mass Spectrometry Analysis of the Proteome

The analysis was performed with the use of Dionex UltiMate 3000 RSLC nanoLC system connected to Q Exactive Orbitrap mass spectrometer (Thermo Fisher Scientific). Peptides derived from in-solution digestion were separated on a reverse phase Acclaim PepMap RSLC nanoViper C18 column (75 μm × 25 cm, 2 μm granulation) using acetonitrile gradient (from 4 to 60%, in 0.1% formic acid) at 30 °C and a flow rate of 300 nl/min (for 230 min). The spectrometer was operated in data-dependent MS/MS mode (tandem mass spectrometry) with survey scans acquired at a resolution of 70,000 at *m*/*z* 200 in MS mode and 17,500 at *m*/*z* 200 in MS2 mode. Spectra were recorded in the scanning range of 300–2000 *m*/*z* in the positive ion mode. Higher energy collisional dissociation (HCD) ion fragmentation was performed with normalized collision energies set to 25. Protein identification was performed using the Swiss-Prot mouse database with a precision tolerance 10 ppm for peptide masses and 0.08 Da for fragment ion masses. All raw data obtained for each dataset were imported into MaxQuant 1.5.3.30 version for protein identification and quantification. Protein was considered as positively identified if at least two peptides per protein were found by Andromeda search engine, and a peptide score reached the significance threshold FDR = 0.01.

Obtained data were exported to Perseus software ver. 1.5.3.2 (part of MaxQuant package). Numeric data were transformed to a logarithmic scale, and each sample was annotated with its group affiliation. Proteins only identified by site, reverse database hits, and contaminants were removed from the results. Next, data were filtered based on valid values for proteins. Proteins which contained valid values in 75% of samples in at least one group (the inclusion threshold: 3 or 4 values for control and 3 or 4 values for Ki91 mice) were included as valid hits. In addition, for supplementary analysis, we have selected proteins which produced valid values only in control or only in Ki91 SCA3/MJD mouse group (4 values inclusion). Prior to statistical analysis, normalization of data was performed by subtracting median from each value in a row. A two-sample *t* test was performed on analyzed sample data with *p* value < 0.05 being considered significant, and differentiating proteins were normalized using the *Z*-score algorithm for hierarchical clustering of data.

### Bioinformatic Analysis of Proteomic Data

The power of the analyses was increased by using two separate set of tools, namely Consensus Path Database (version 32) (CPDB) [90] and Cytoscape version (version 3.6.0) containing the ClueGO plugin (academic version 2.3.5) [91, 92]. CPDB consolidates information from a considerably higher number of databases compared to ClueGO; however, the advantage of ClueGO is its ability to merge the same or similar information about pathways or GO terms which forms general categories. The analysis paradigm included the discovery of affected pathways, molecular function of dysregulated proteins, biological processes affected by the dysregulated proteins, and prediction of subcellular localization of dysregulated proteins. For each analysis, names of genes corresponding to the names of dysregulated proteins or phosphoproteins were used.

Proteins were grouped using the Consensus Path Database according to the pathways (pathway enrichment *p* value cutoff < 0.01, minimum overlap with input list = 10% of total number of dysregulated proteins), GO term analyses by molecular function, biological process, and cellular component (GO term B, MF level 5, CC levels 4 and 5, *p* value cutoff < 0.0001).

The second analysis in CPDB was performed with lists of top dysregulated proteins (log2 FC ≤ − 1.0 for downregulated protein levels or log2 FC ≥ 1.0 for upregulated protein levels) for pathway enrichment and GO terms with the same *p* value and minimum overlap with input list restrictions.

Subsequently, using ClueGO and Cytoscape, we have performed GO term annotation analysis and pathway enrichment (*p* value cutoff < 0.05, GO tree interval = 3–5, kappa score = 0.5). In ClueGO, the number of GO terms was restricted by the number of dysregulated proteins per GO term in proportional relation to the total number of dysregulated proteins in a dataset (51–100 dysregulated proteins—inclusion of GO terms containing a minimum of 3 proteins from the input list, 101–150—minimum of 4 proteins, 301–350—minimum of 8 proteins). The analysis in ClueGO was based on “biological process,” “molecular function,” and KEGG, Reactome, and WikiPathways [93–94]. The compatibility of results obtained with the Consensus Path Database and ClueGO was confirmed by identification of common GO terms with similar *p* values using both tools.

In a separate analysis, we performed identification of cell types in the brain which are affected by pathogenesis using the BrainMap tool of *Allen Brain Atlas* [22] for the cerebral cortex and the DropViz tool [21] for the cerebellum. This was accomplished by grouping of dysregulated proteins according to cell type in the brain in which they are expressed. Significantly dysregulated (*p* < 0.05) proteins identified from label-free proteomic and phosphoproteomic LCMS experiments were included together into two tissue groups, based on origin from the cerebellum and cortex. Relative expression is presented in BrainMap as counts per million in log10 scale and in DropViz as the amount of transcripts per 100,000 in the cluster. Moreover, for the phosphoproteomic data, we have predicted kinases which phosphorylate dysregulated phosphoproteins using CPDB and PHOSIDA (using known motifs) [95]. For the analysis with PHOSIDA sequences of peptides with altered phosphorylation level, the FASTA format was used.

### Neural Cultures from SCA3 Patients iPSC

The culture and differentiation of iPSC from SCA3 patients were performed as previously described [24]. Picked neural rosettes were cultured in suspension for 2 days in DMEM/F12, 2 mM l-glutamine, 1.6 g l^−1^ glucose, 0.1 mg ml^−1^ penicillin/streptomycin, and N2 supplement (1:100; Invitrogen), then dissociated with trypsin, plated onto poly-l-ornithine/laminin-coated plates, and propagated in N2 medium supplemented with B27 (1 μl ml^−1^, Invitrogen), 10 ng ml^−1^ FGF2, and 10 ng ml^−1^ EGF (both from R&D Systems). Neuronal differentiation was induced by removing the growth factors FGF2 and EGF from the media and culturing the cells in Neurobasal medium supplemented with B27 (1:50, Invitrogen) and DMEM/F12 supplemented with N2 (1:100) mixed at a 1:1 ratio; 300 ng ml^−1^ cAMP was added to the differentiation media. After 6 weeks, differentiation cells were harvested in TRIzol.

### Immunohistochemistry

The animals were deeply anesthetized and transcardially perfused using saline followed by 4% PFA. The brains were removed, postfixed in 4% PFA for 48 h, and cryopreserved with graded sucrose (10–20–30%) over 72 h. The 20-μm parasagittal mouse brain sections were cut using a cryostat at − 20 °C and collected on SuperFrost Plus slides (Thermo Scientific). The sections were processed immediately. The HIER procedure was applied by incubation of the sections in citrate buffer (pH 9.0) for 30 min at 60 °C. The sections were blocked via incubation in 4% normal goat serum in TBS for 1 h. For immunofluorescence staining, the sections were incubated overnight at 4 °C with the primary mouse anti-ataxin-3 antibody 1H9 (1:200) [] and, subsequently, with the anti-mouse antibody labeled by AlexaFluor488 (1:400; Jackson ImmunoResearch, Suffolk, UK). The sections were end-stained with Hoechst 33342 (Sigma) nuclear stain at 1:1000 and embedded in Fluoroshield (Sigma) mounting medium. Fluorescent confocal images were acquired using fixed excitation and detection parameters using the TCS SP5 II (Leica Microsystems, Poland).

### Statistics

The data regarding behavioral experiments performed within 4 days (rotarod, elevated beam walk, parallel rod floor test) were subjected to a two-way ANOVA, followed by Bonferroni posttests. Scoring test and body weight were evaluated with unpaired Student’s *t* test. The two-group comparisons of the gene expression data by qPCR were conducted using the unpaired Student’s *t* test. *p* values less than 0.05 were considered significant with the exception of qPCR using C57BL/6 and FVB controls, where *p* values less than 0.01 were considered statistically significant. Identification of proteins on raw proteomic data was performed by Andromeda search engine in Mascot using the following inclusion criteria: 1. At least two different peptides per protein were identified per sample, and a total peptide score reached the significance threshold FDR = 0.01. Identified proteins matching the inclusion criteria were subjected to further statistical analysis with two-sample *t* test, and dysregulation of protein level reaching *p* value < 0.05 was considered as significant. One asterisk indicates *p* value ≤ 0.05, two asterisks indicate *p* value ≤ 0.01, and three asterisks indicate *p* value ≤ 0.001.

## Electronic Supplementary Material

ESM 1 (PDF 3747 kb)

ESM 2 (DOCX 36 kb)

Supplementary Table 1 List of the animals sacrificed for experiments (DOCX 22 kb)

Supplementary Table 2 Primers used for qPCR validation of RNA sequencing result and designed based on proteomic analysis data (DOCX 25 kb)

Supplementary Table 3 The summary of analysis of GO terms and pathways containing all dysregulated proteins and phosphoproteins from cerebellum and cerebral cortex performed in ClueGO (Cytoscape). (XLSX 86 kb)

Supplementary Table 4 The summary of analysis of pathways containing all and top dysregulated proteins and phosphoproteins from cerebellum and cerebral cortex performed in Consensus Path Database. (XLSX 129 kb)

Supplementary Table 5 The summary of analysis of GO terms containing all and top dysregulated proteins and phosphoproteins from cerebellum and cerebral cortex performed in Consensus Path Database. (XLSX 136 kb)

Supplementary Table 6 Proteomic and phosphoproteomic data analyzed with MaxQuant and Perseus software. (XLSX 225 kb)

Supplementary Table 7 Subcellular localization of dysregulated proteins and phosphoproteins performed in Consensus Path Database (GO terms CC). (XLSX 38 kb)

## Abbreviations

*SCA3*: spinocerebellar ataxia type 3
*MJD*: Machado–Joseph disease
*polyQ*: polyglutamine
*iPSC*: induced pluripotent stem cell
*MS*: mass spectrometry
*FC*: fold change

## References

[1] Kawaguchi Y, Okamoto T, Taniwaki M, Aizawa M, Inoue M, Katayama S, Kawakami H, Nakamura S, Nishimura M, Akiguchi I, Kimura J, Narumiya S, Kakizuka A (1994). CAG expansions in a novel gene for Machado-Joseph disease at chromosome 14q32.1. Nat Genet.

[2] Riess O, Rüb U, Pastore A, Bauer P, Schöls L (2008). SCA3: neurological features, pathogenesis and animal models. Cerebellum.

[3] Winborn BJ, Travis SM, Todi SV, Scaglione KM, Xu P, Williams AJ, Cohen RE, Peng J, Paulson HL (2008). The deubiquitinating enzyme ataxin-3, a polyglutamine disease protein, edits lys63 linkages in mixed linkage ubiquitin chains. J Biol Chem.

[4] Todi SV, Scaglione KM, Blount JR, Basrur V, Conlon KP, Pastore A, Elenitoba-Johnson K, Paulson HL (2010). Activity and cellular functions of the deubiquitinating enzyme and polyglutamine disease protein ataxin-3 are regulated by ubiquitination at lysine 117. J Biol Chem.

[5] Weishäupl D, Schneider J, Peixoto Pinheiro B, Ruess C, Dold SM, von Zweydorf F, Gloeckner CJ, Schmidt J, Riess O, Schmidt T (2019). Physiological and pathophysiological characteristics of ataxin-3 isoforms. J Biol Chem.

[6] Matos CA, de Almeida LP, Nóbrega C (2019). Machado-Joseph disease/spinocerebellar ataxia type 3: lessons from disease pathogenesis and clues into therapy. J Neurochem.

[7] Seidel K, den Dunnen WFA, Schultz C, Paulson H, Frank S, de Vos RA, Brunt ER, Deller T, Kampinga HH, Rüb U (2010). Axonal inclusions in spinocerebellar ataxia type 3. Acta Neuropathol.

[8] Sowa AS, Martin E, Martins IM, Schmidt J, Depping R, Weber JJ, Rother F, Hartmann E, Bader M, Riess O, Tricoire H, Schmidt T (2018). Karyopherin α-3 is a key protein in the pathogenesis of spinocerebellar ataxia type 3 controlling the nuclear localization of ataxin-3. Proc Natl Acad Sci U S A.

[9] Araujo J, Breuer P, Dieringer S, Krauss S, Dorn S, Zimmermann K, Pfeifer A, Klockgether T, Wuellner U, Evert BO (2011). FOXO4-dependent upregulation of superoxide dismutase-2 in response to oxidative stress is impaired in spinocerebellar ataxia type 3. Hum Mol Genet.

[10] Hübener J, Weber JJ, Richter C (2013). Calpain-mediated ataxin-3 cleavage in the molecular pathogenesis of spinocerebellar ataxia type 3 (SCA3). Hum Mol Genet.

[11] Nascimento-Ferreira I, Santos-Ferreira T, Sousa-Ferreira L, Auregan G, Onofre I, Alves S, Dufour N, Colomer Gould VF, Koeppen A, Déglon N, Pereira de Almeida L (2011). Overexpression of the autophagic beclin-1 protein clears mutant ataxin-3 and alleviates Machado-Joseph disease. Brain J Neurol.

[12] Nóbrega C, Simões AT, Duarte-Neves J (2018). Molecular mechanisms and cellular pathways implicated in Machado-Joseph disease pathogenesis. Adv Exp Med Biol.

[13] Switonski P, Szlachcic W, Gabka A (2012). Mouse models of polyglutamine diseases in therapeutic approaches: review and data table. Part II. Mol Neurobiol.

[14] Switonski PM, Szlachcic WJ, Krzyzosiak WJ, Figiel M (2015). A new humanized ataxin-3 knock-in mouse model combines the genetic features, pathogenesis of neurons and glia and late disease onset of SCA3/MJD. Neurobiol Dis.

[15] Figiel M, Szlachcic W, Switonski P (2012). Mouse models of polyglutamine diseases: review and data table. Part I. Mol Neurobiol.

[16] Watkins-Chow DE, Pavan WJ (2008). Genomic copy number and expression variation within the C57BL/6J inbred mouse strain. Genome Res.

[17] Leite C de MBA, Schieferdecker MEM, Frehner C (2018). Body composition in spinocerebellar ataxia type 3 and 10 patients: comparative study with control group. Nutr Neurosci.

[18] Saute JAM, da Silva ACF, Souza GN, Russo AD, Donis KC, Vedolin L, Saraiva-Pereira ML, Portela LVC, Jardim LB (2012). Body mass index is inversely correlated with the expanded CAG repeat length in SCA3/MJD patients. Cerebellum.

[19] Yang J-S, Chen P-P, Lin M-T, Qian MZ, Lin HX, Chen XP, Shang XJ, Wang DN, Chen YC, Jiang B, Chen YJ, Wang N, Chen WJ, Gan SR (2018). Association between body mass index and disease severity in Chinese spinocerebellar ataxia type 3 patients. Cerebellum.

[20] Diallo A, Jacobi H, Schmitz-Hübsch T, Cook A, Labrum R, Durr A, Brice A, Charles P, Marelli C, Mariotti C, Nanetti L, Panzeri M, Rakowicz M, Sobanska A, Sulek A, Schöls L, Hengel H, Melegh B, Filla A, Antenora A, Infante J, Berciano J, van de Warrenburg BP, Timmann D, Boesch S, Pandolfo M, Schulz JB, Bauer P, Giunti P, Baliko L, Parkinson MH, Kang JS, Klockgether T, Tezenas du Montcel S (2017). Body mass index decline is related to spinocerebellar ataxia disease progression. Mov Disord Clin Pract.

[21] Saunders A, Macosko EZ, Wysoker A, Goldman M, Krienen FM, de Rivera H, Bien E, Baum M, Bortolin L, Wang S, Goeva A, Nemesh J, Kamitaki N, Brumbaugh S, Kulp D, McCarroll SA (2018). Molecular diversity and specializations among the cells of the adult mouse brain. Cell.

[22] Tasic B, Yao Z, Graybuck LT, Smith KA, Nguyen TN, Bertagnolli D, Goldy J, Garren E, Economo MN, Viswanathan S, Penn O, Bakken T, Menon V, Miller J, Fong O, Hirokawa KE, Lathia K, Rimorin C, Tieu M, Larsen R, Casper T, Barkan E, Kroll M, Parry S, Shapovalova NV, Hirschstein D, Pendergraft J, Sullivan HA, Kim TK, Szafer A, Dee N, Groblewski P, Wickersham I, Cetin A, Harris JA, Levi BP, Sunkin SM, Madisen L, Daigle TL, Looger L, Bernard A, Phillips J, Lein E, Hawrylycz M, Svoboda K, Jones AR, Koch C, Zeng H (2018). Shared and distinct transcriptomic cell types across neocortical areas. Nature.

[23] Koch P, Breuer P, Peitz M, Jungverdorben J, Kesavan J, Poppe D, Doerr J, Ladewig J, Mertens J, Tüting T, Hoffmann P, Klockgether T, Evert BO, Wüllner U, Brüstle O (2011). Excitation-induced ataxin-3 aggregation in neurons from patients with Machado-Joseph disease. Nature.

[24] Koch P, Opitz T, Steinbeck JA, Ladewig J, Brustle O (2009). A rosette-type, self-renewing human ES cell-derived neural stem cell with potential for in vitro instruction and synaptic integration. Proc Natl Acad Sci U S A.

[25] Bateman A, Martin MJ, O’Donovan C (2017). UniProt: the universal protein knowledgebase. Nucleic Acids Res.

[26] Kini HK, Silverman IM, Ji X, Gregory BD, Liebhaber SA (2016). Cytoplasmic poly(A) binding protein-1 binds to genomically encoded sequences within mammalian mRNAs. RNA.

[27] Hu Z, Holzschuh J, Driever W (2015). Loss of DDB1 leads to transcriptional p53 pathway activation in proliferating cells, cell cycle deregulation, and apoptosis in zebrafish embryos. PLoS One.

[28] Boggs JM (2006). Myelin basic protein: a multifunctional protein. Cell Mol Life Sci.

[29] Kapitein LC, Hoogenraad CC (2015). Building the neuronal microtubule cytoskeleton. Neuron.

[30] Yuan A, Rao MV, Veeranna null, Nixon RA (2017) Neurofilaments and neurofilament proteins in health and disease. Cold Spring Harb Perspect Biol 9:. 10.1101/cshperspect.a018309 10.1101/cshperspect.a018309 PMC5378049 28373358

[31] Boeing S, Williamson L, Encheva V, Gori I, Saunders RE, Instrell R, Aygün O, Rodriguez-Martinez M, Weems JC, Kelly GP, Conaway JW, Conaway RC, Stewart A, Howell M, Snijders AP, Svejstrup JQ (2016) Multiomic Analysis of the UV-Induced DNA Damage Response. Cell Reports 15 (7):1597-1610 10.1016/j.celrep.2016.04.047 PMC4893159 27184836

[32] Zhang H, Head PE, Yu DS (2016) SIRT2 orchestrates the DNA damage response. Cell Cycle 15 (16):2089-2090 10.1080/15384101.2016.1184517 PMC4993535 27153288

[33] Vohhodina J, Barros EM, AL S, Liberante FG, Manti L, Bankhead P, Cosgrove N, Madden AF, Harkin PD, Savage KI (2017) The RNA processing factors THRAP3 and BCLAF1 promote the DNA damage response through selective mRNA splicing and nuclear export. Nucleic Acids Research 45 (22):12816-12833 10.1093/nar/gkx1046 PMC5728405 29112714

[34] Lomonosova E, Chinnadurai G (2008) BH3-only proteins in apoptosis and beyond: an overview. Oncogene 27 Suppl 1:S2–19. 10.1038/onc.2009.39 10.1038/onc.2009.39 PMC2928556 19641503

[35] Wang T-Y, Jao C-W, Soong B-W, Wu HM, Shyu KK, Wang PS, Wu YT (2015). Change in the cortical complexity of spinocerebellar ataxia type 3 appears earlier than clinical symptoms. PLoS One.

[36] Lu M-K, Chen J-C, Chen C-M, Duann JR, Ziemann U, Tsai CH (2017). Impaired cerebellum to primary motor cortex associative plasticity in Parkinson’s disease and spinocerebellar ataxia type 3. Front Neurol.

[37] Farrar MA, Vucic S, Nicholson G, Kiernan MC (2016) Motor cortical dysfunction develops in spinocerebellar ataxia type 3. Clin Neurophysiol 127:3418–3424. 10.1016/j.clinph.2016.09.005 10.1016/j.clinph.2016.09.005 27689815

[38] de Rezende TJR, D’Abreu A, Guimarães RP (2015). Cerebral cortex involvement in Machado-Joseph disease. Eur J Neurol.

[39] Schmidt J, Mayer AK, Bakula D, Freude J, Weber JJ, Weiss A, Riess O, Schmidt T (2018). Vulnerability of frontal brain neurons for the toxicity of expanded ataxin-3. Hum Mol Genet.

[40] Chou A-H, Chen Y-L, Hu S-H, Chang YM, Wang HL (2014). Polyglutamine-expanded ataxin-3 impairs long-term depression in Purkinje neurons of SCA3 transgenic mouse by inhibiting HAT and impairing histone acetylation. Brain Res.

[41] Evert BO, Araujo J, Vieira-Saecker AM, de Vos RAI, Harendza S, Klockgether T, Wullner U (2006). Ataxin-3 represses transcription via chromatin binding, interaction with histone deacetylase 3, and histone deacetylation. J Neurosci.

[42] Evert BO, Vogt IR, Vieira-Saecker AM, Ozimek L, de Vos RAI, Brunt ERP, Klockgether T, Wüllner U (2003). Gene expression profiling in ataxin-3 expressing cell lines reveals distinct effects of normal and mutant ataxin-3. J Neuropathol Exp Neurol.

[43] Estebanez L, Hoffmann D, Voigt BC, Poulet JFA (2017). Parvalbumin-expressing GABAergic neurons in primary motor cortex signal reaching. Cell Rep.

[44] Yu MC, Cho E, Luo CB, Li WWY, Shen WZ, Yew DT (1996). Immunohistochemical studies of GABA and parvalbumin in the developing human cerebellum. Neuroscience.

[45] Kazachkova N, Raposo M, Ramos A, Montiel R, Lima M (2017) Promoter variant alters expression of the autophagic BECN1 gene: implications for clinical manifestations of Machado-Joseph disease. Cerebellum 16:957–963. 10.1007/s12311-017-0875-4 10.1007/s12311-017-0875-4 28699106

[46] Ramani B, Panwar B, Moore LR, Wang B, Huang R, Guan Y, Paulson HL (2017). Comparison of spinocerebellar ataxia type 3 mouse models identifies early gain-of-function, cell-autonomous transcriptional changes in oligodendrocytes. Hum Mol Genet.

[47] Raposo M, Bettencourt C, Ramos A, Kazachkova N, Vasconcelos J, Kay T, Bruges-Armas J, Lima M (2017). Promoter variation and expression levels of inflammatory genes IL1A, IL1B, IL6 and TNF in blood of spinocerebellar ataxia type 3 (SCA3) patients. NeuroMolecular Med.

[48] Toonen LJA, Overzier M, Evers MM, Leon LG, van der Zeeuw SAJ, Mei H, Kielbasa SM, Goeman JJ, Hettne KM, Magnusson OT, Poirel M, Seyer A, ‘t Hoen PAC, van Roon-Mom WMC (2018). Transcriptional profiling and biomarker identification reveal tissue specific effects of expanded ataxin-3 in a spinocerebellar ataxia type 3 mouse model. Mol Neurodegener.

[49] Gibson EM, Purger D, Mount CW, Goldstein AK, Lin GL, Wood LS, Inema I, Miller SE, Bieri G, Zuchero JB, Barres BA, Woo PJ, Vogel H, Monje M (2014). Neuronal activity promotes oligodendrogenesis and adaptive myelination in the mammalian brain. Science.

[50] Fei E, Jia N, Zhang T, Ma X, Wang H, Liu C, Zhang W, Ding L, Nukina N, Wang G (2007). Phosphorylation of ataxin-3 by glycogen synthase kinase 3beta at serine 256 regulates the aggregation of ataxin-3. Biochem Biophys Res Commun.

[51] Kristensen LV, Oppermann FS, Rauen MJ, Fog K, Schmidt T, Schmidt J, Harmuth T, Hartmann-Petersen R, Thirstrup K (2018). Mass spectrometry analyses of normal and polyglutamine expanded ataxin-3 reveal novel interaction partners involved in mitochondrial function. Neurochem Int.

[52] Tao R-S, Fei E-K, Ying Z, Wang HF, Wang GH (2008). Casein kinase 2 interacts with and phosphorylates ataxin-3. Neurosci Bull.

[53] Tu Y, Liu H, Zhu X, Shen H, Ma X, Wang F, Huang M, Gong J, Li X, Wang Y, Guo C, Tang TS (2017). Ataxin-3 promotes genome integrity by stabilizing Chk1. Nucleic Acids Res.

[54] Vinayagam A, Stelzl U, Foulle R, Plassmann S, Zenkner M, Timm J, Assmus HE, Andrade-Navarro MA, Wanker EE (2011). A directed protein interaction network for investigating intracellular signal transduction. Sci Signal.

[55] Kim Y-B, Shin YJ, Roy A, Kim J-H (2015). The role of the Pleckstrin homology domain-containing protein CKIP-1 in activation of p21-activated kinase 1 (PAK1). J Biol Chem.

[56] Kim H, Oh J-Y, Choi S-L, Nam YJ, Jo A, Kwon A, Shin EY, Kim EG, Kim HK (2016). Down-regulation of p21-activated serine/threonine kinase 1 is involved in loss of mesencephalic dopamine neurons. Mol Brain.

[57] Ma Q-L, Yang F, Frautschy SA, Cole GM (2012). PAK in Alzheimer disease, Huntington disease and X-linked mental retardation. Cell Logist.

[58] Luo S, Mizuta H, Rubinsztein DC (2008). p21-activated kinase 1 promotes soluble mutant huntingtin self-interaction and enhances toxicity. Hum Mol Genet.

[59] Wlodarchak N, Xing Y (2016). PP2A as a master regulator of the cell cycle. Crit Rev Biochem Mol Biol.

[60] Mazzucchelli S, De Palma A, Riva M (2009). Proteomic and biochemical analyses unveil tight interaction of ataxin-3 with tubulin. Int J Biochem Cell Biol.

[61] Pozzi C, Valtorta M, Tedeschi G, Galbusera E, Pastori V, Bigi A, Nonnis S, Grassi E, Fusi P (2008). Study of subcellular localization and proteolysis of ataxin-3. Neurobiol Dis.

[62] Ding Y, Li Y, Lu L, Zhang R, Zeng L, Wang L, Zhang X (2015). Inhibition of Nischarin expression promotes neurite outgrowth through regulation of PAK activity. PLoS One.

[63] Poplawski GHD, Tranziska A-K, Leshchyns’ka I (2012). L1CAM increases MAP2 expression via the MAPK pathway to promote neurite outgrowth. Mol Cell Neurosci.

[64] Sample V, Ramamurthy S, Gorshkov K, Ronnett GV, Zhang J (2015). Polarized activities of AMPK and BRSK in primary hippocampal neurons. Mol Biol Cell.

[65] Toriyama M, Kozawa S, Sakumura Y, Inagaki N (2013). Conversion of a signal into forces for axon outgrowth through Pak1-mediated shootin1 phosphorylation. Curr Biol.

[66] Winograd-Katz SE, Brunner MC, Mirlas N, Geiger B (2011). Analysis of the signaling pathways regulating Src-dependent remodeling of the actin cytoskeleton. Eur J Cell Biol.

[67] Rankin CA, Sun Q, Gamblin TC (2008). Pre-assembled tau filaments phosphorylated by GSK-3b form large tangle-like structures. Neurobiol Dis.

[68] Rousseaux MW, de Haro M, Lasagna-Reeves CA, et al (2016) TRIM28 regulates the nuclear accumulation and toxicity of both alpha-synuclein and tau. eLife 5:. 10.7554/eLife.19809 10.7554/eLife.19809 PMC5104516 27779468

[69] Chatterjee A, Saha S, Chakraborty A, Silva-Fernandes A, Mandal SM, Neves-Carvalho A, Liu Y, Pandita RK, Hegde ML, Hegde PM, Boldogh I, Ashizawa T, Koeppen AH, Pandita TK, Maciel P, Sarkar PS, Hazra TK (2015). The role of the mammalian DNA end-processing enzyme polynucleotide kinase 3’-phosphatase in spinocerebellar ataxia type 3 pathogenesis. PLoS Genet.

[70] Gao R, Liu Y, Silva-Fernandes A, Fang X, Paulucci-Holthauzen A, Chatterjee A, Zhang HL, Matsuura T, Choudhary S, Ashizawa T, Koeppen AH, Maciel P, Hazra TK, Sarkar PS (2015). Inactivation of PNKP by mutant ATXN3 triggers apoptosis by activating the DNA damage-response pathway in SCA3. PLoS Genet.

[71] Peixeiro I, Inácio Â, Barbosa C (2012). Interaction of PABPC1 with the translation initiation complex is critical to the NMD resistance of AUG-proximal nonsense mutations. Nucleic Acids Res.

[72] Nawaz MS, Vik ES, Berges N, Fladeby C, Bjørås M, Dalhus B, Alseth I (2016). Regulation of human endonuclease V activity and relocalization to cytoplasmic stress granules. J Biol Chem.

[73] Chen L, Stone MC, Tao J, Rolls MM (2012). Axon injury and stress trigger a microtubule-based neuroprotective pathway. Proc Natl Acad Sci U S A.

[74] Chou A-H, Yeh T-H, Kuo Y-L, Kao YC, Jou MJ, Hsu CY, Tsai SR, Kakizuka A, Wang HL (2006). Polyglutamine-expanded ataxin-3 activates mitochondrial apoptotic pathway by upregulating Bax and downregulating Bcl-xL. Neurobiol Dis.

[75] Hsu J-Y, Jhang Y-L, Cheng P-H, Chang YF, Mao SH, Yang HI, Lin CW, Chen CM, Yang SH (2017). The truncated C-terminal fragment of mutant ATXN3 disrupts mitochondria dynamics in spinocerebellar ataxia type 3 models. Front Mol Neurosci.

[76] Yu Y-C, Kuo C-L, Cheng W-L, Liu CS, Hsieh M (2009). Decreased antioxidant enzyme activity and increased mitochondrial DNA damage in cellular models of Machado-Joseph disease. J Neurosci Res.

[77] Wang X, Chen XJ (2015). A cytosolic network suppressing mitochondria-mediated proteostatic stress and cell death. Nature.

[78] Guyenet SJ, Furrer SA, Damian VM, Baughan TD, la Spada AR, Garden GA (2010) A simple composite phenotype scoring system for evaluating mouse models of cerebellar ataxia. J Vis Exp:e1787–e1787. 10.3791/1787 10.3791/1787 PMC3121238 20495529

[79] Patel RK, Jain M (2012). NGS QC toolkit: a toolkit for quality control of next generation sequencing data. PLoS One.

[80] Dobin A, Davis CA, Schlesinger F, Drenkow J, Zaleski C, Jha S, Batut P, Chaisson M, Gingeras TR (2013). STAR: ultrafast universal RNA-seq aligner. Bioinforma.

[81] Hartley SW, Mullikin JC (2015). QoRTs: a comprehensive toolset for quality control and data processing of RNA-Seq experiments. BMC Bioinformatics.

[82] Kim D, Langmead B, Salzberg SL (2015). HISAT: a fast spliced aligner with low memory requirements. Nat Methods.

[83] Pertea M, Kim D, Pertea GM, Leek JT, Salzberg SL (2016). Transcript-level expression analysis of RNA-seq experiments with HISAT, StringTie and Ballgown. Nat Protoc.

[84] Love MI, Huber W, Anders S (2014). Moderated estimation of fold change and dispersion for RNA-seq data with DESeq2. Genome Biol.

[85] Pertea M, Pertea GM, Antonescu CM, Chang TC, Mendell JT, Salzberg SL (2015). StringTie enables improved reconstruction of a transcriptome from RNA-seq reads. Nat Biotechnol.

[86] Hartley SW, Mullikin JC (2016). Detection and visualization of differential splicing in RNA-Seq data with JunctionSeq. Nucleic Acids Res.

[87] Bustin SA, Benes V, Garson JA, Hellemans J, Huggett J, Kubista M, Mueller R, Nolan T, Pfaffl MW, Shipley GL, Vandesompele J, Wittwer CT (2009). The MIQE guidelines: minimum information for publication of quantitative real-time PCR experiments. Clin Chem.

[88] Trottier Y, Cancel G, An-Gourfinkel I, Lutz Y, Weber C, Brice A, Hirsch E, Mandel JL (1998). Heterogeneous intracellular localization and expression of ataxin-3. Neurobiol Dis.

[89] Kanehisa M, Furumichi M, Tanabe M, Sato Y, Morishima K (2017). KEGG: new perspectives on genomes, pathways, diseases and drugs. Nucleic Acids Res.

[90] Herwig R, Hardt C, Lienhard M, Kamburov A (2016). Analyzing and interpreting genome data at the network level with ConsensusPathDB. Nat Protoc.

[91] Bindea G, Galon J, Mlecnik B (2013). CluePedia Cytoscape plugin: pathway insights using integrated experimental and in silico data. Bioinforma.

[92] Bindea G, Mlecnik B, Hackl H, Charoentong P, Tosolini M, Kirilovsky A, Fridman WH, Pagès F, Trajanoski Z, Galon J (2009). ClueGO: a Cytoscape plug-in to decipher functionally grouped gene ontology and pathway annotation networks. Bioinforma.

[93] Fabregat A, Jupe S, Matthews L, Sidiropoulos K, Gillespie M, Garapati P, Haw R, Jassal B, Korninger F, May B, Milacic M, Roca CD, Rothfels K, Sevilla C, Shamovsky V, Shorser S, Varusai T, Viteri G, Weiser J, Wu G, Stein L, Hermjakob H, D’Eustachio P (2018). The Reactome pathway knowledgebase. Nucleic Acids Res.

[94] Slenter DN, Kutmon M, Hanspers K, Riutta A, Windsor J, Nunes N, Mélius J, Cirillo E, Coort SL, Digles D, Ehrhart F, Giesbertz P, Kalafati M, Martens M, Miller R, Nishida K, Rieswijk L, Waagmeester A, Eijssen LMT, Evelo CT, Pico AR, Willighagen EL (2018). WikiPathways: a multifaceted pathway database bridging metabolomics to other omics research. Nucleic Acids Res.

[95] Gnad F, Ren S, Cox J, Olsen JV, Macek B, Oroshi M, Mann M (2007). PHOSIDA (phosphorylation site database): management, structural and evolutionary investigation, and prediction of phosphosites. Genome Biol.

[96] J. J. Lin, (1981) Monoclonal antibodies against myofibrillar components of rat skeletal muscle decorate the intermediate filaments of cultured cells. Proceedings of the National Academy of Sciences 78 (4):2335-2339 10.1073/pnas.78.4.2335 PMC319340 7017730

[97] Tapia-Rojas C, Cabezas-Opazo F, Deaton CA, Vergara EH, Johnson GVW, Quintanilla RA (2019) It’s all about tau. Progress in Neurobiology 175:54-76 10.1016/j.pneurobio.2018.12.005 PMC6397676 30605723

[98] Wood JN, Anderton BH (1981) Monoclonal antibodies to mammalian neurofilaments. Bioscience Reports 1 (3):263-268 10.1007/BF01114913 7028152

