# Supplementary material for: Altered Levels of Proteins and Phosphoproteins, in the Absence of Early Causative Transcriptional Changes, Shape the Molecular Pathogenesis in the Brain of Young Presymptomatic Ki91 SCA3/MJD Mouse
Source: Mol Neurobiol. 2019 Jun 14;56(12):8168–202. doi: 10.1007/s12035-019-01643-4 (PMC6834541; doi:10.1007/s12035-019-01643-4)

Supplementary Figure 1

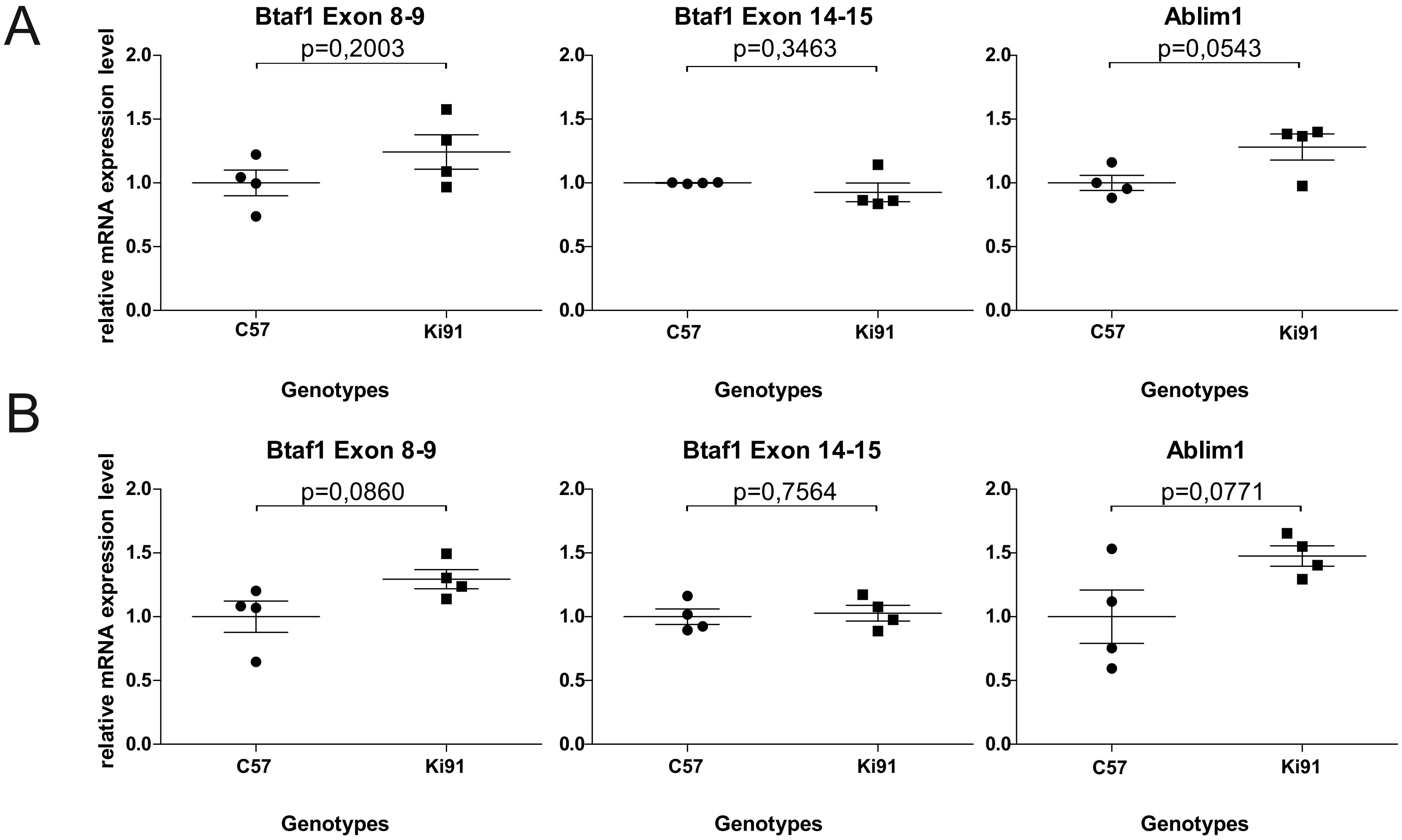

Supplementary figure 2A

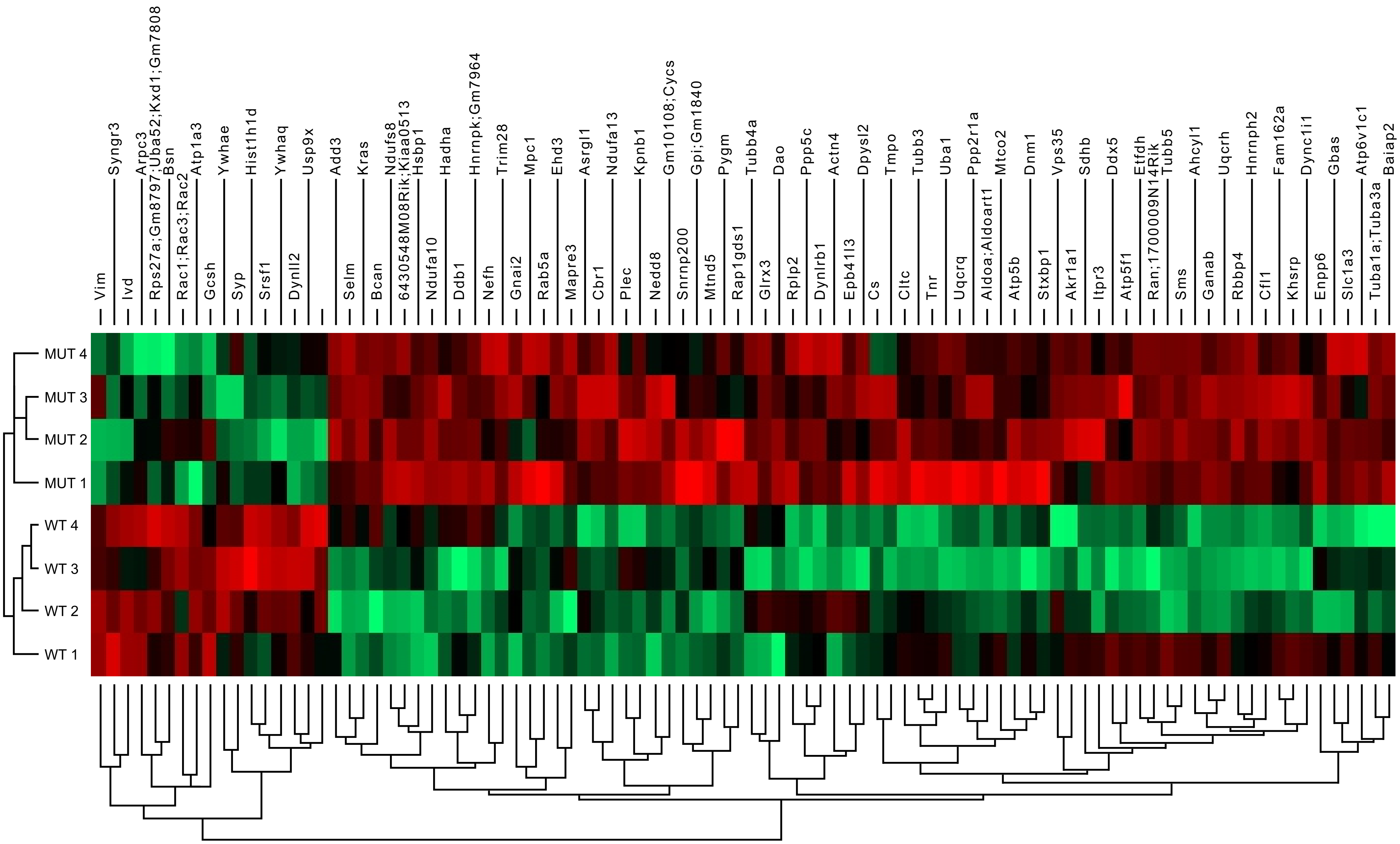

cerebellum - total proteins

Supplementary figure 2B

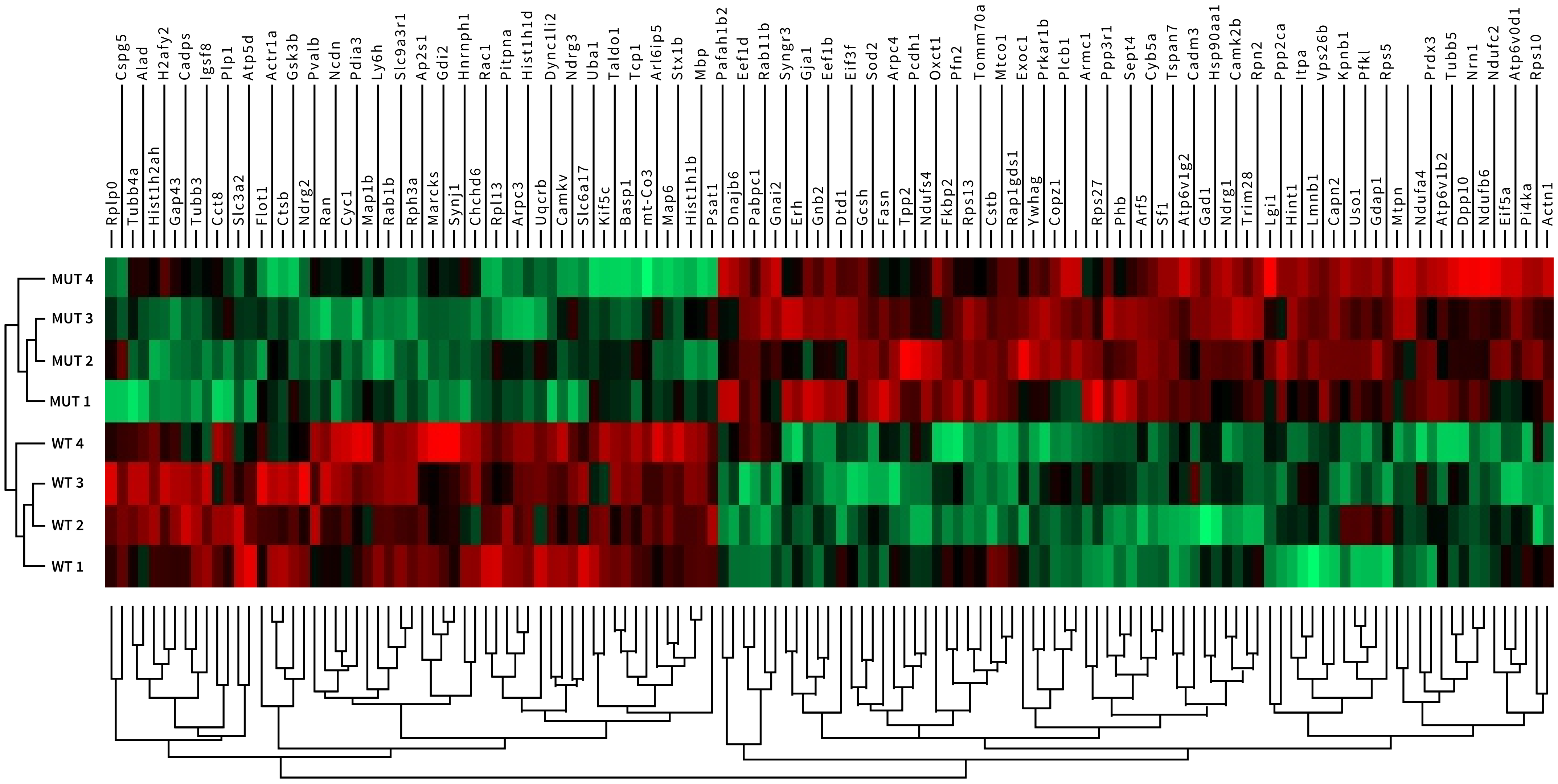

cerebral cortex - total proteins

Supplementary figure 2C

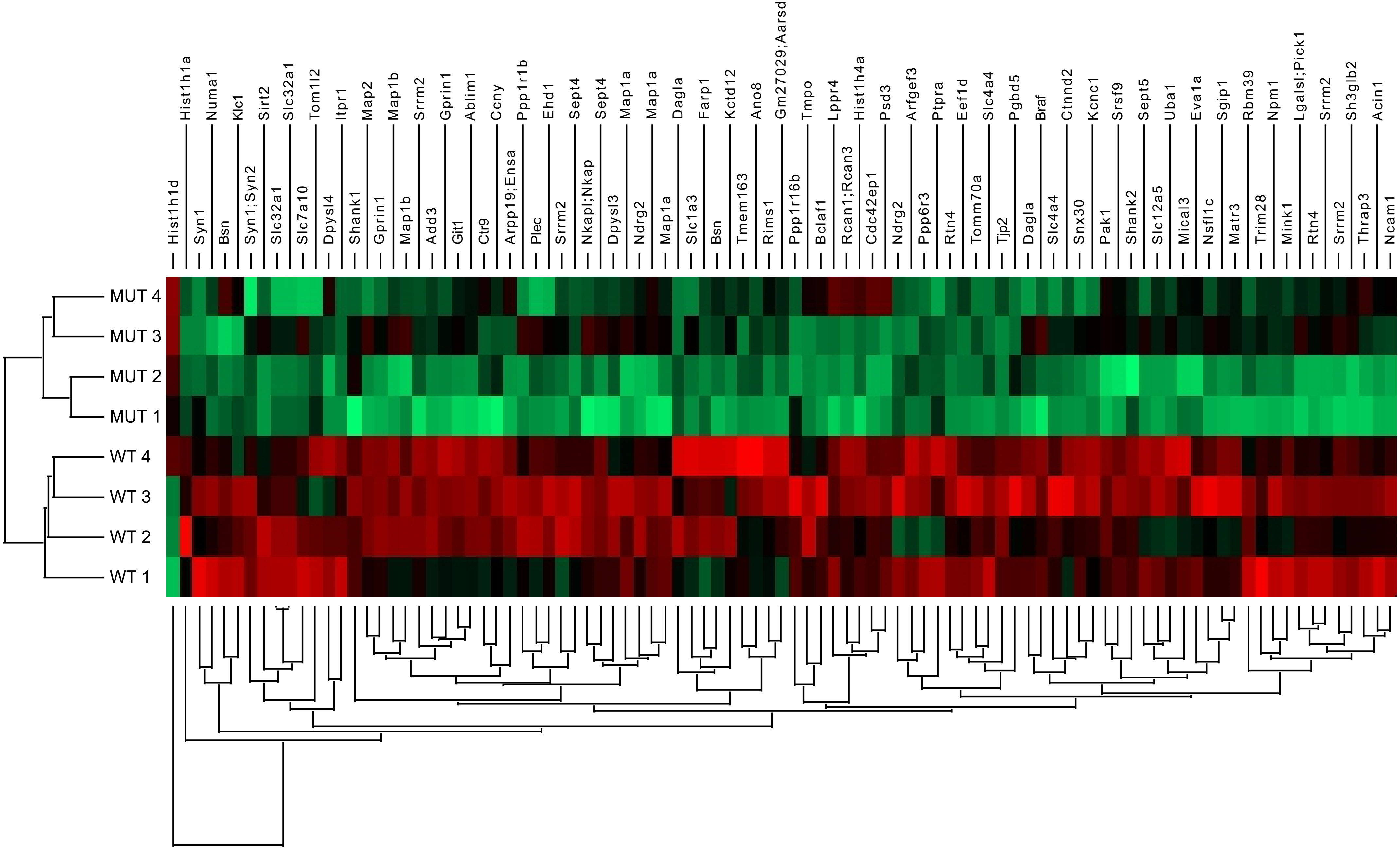

cerebellum - phosphoproteins

Supplementary figure 2D

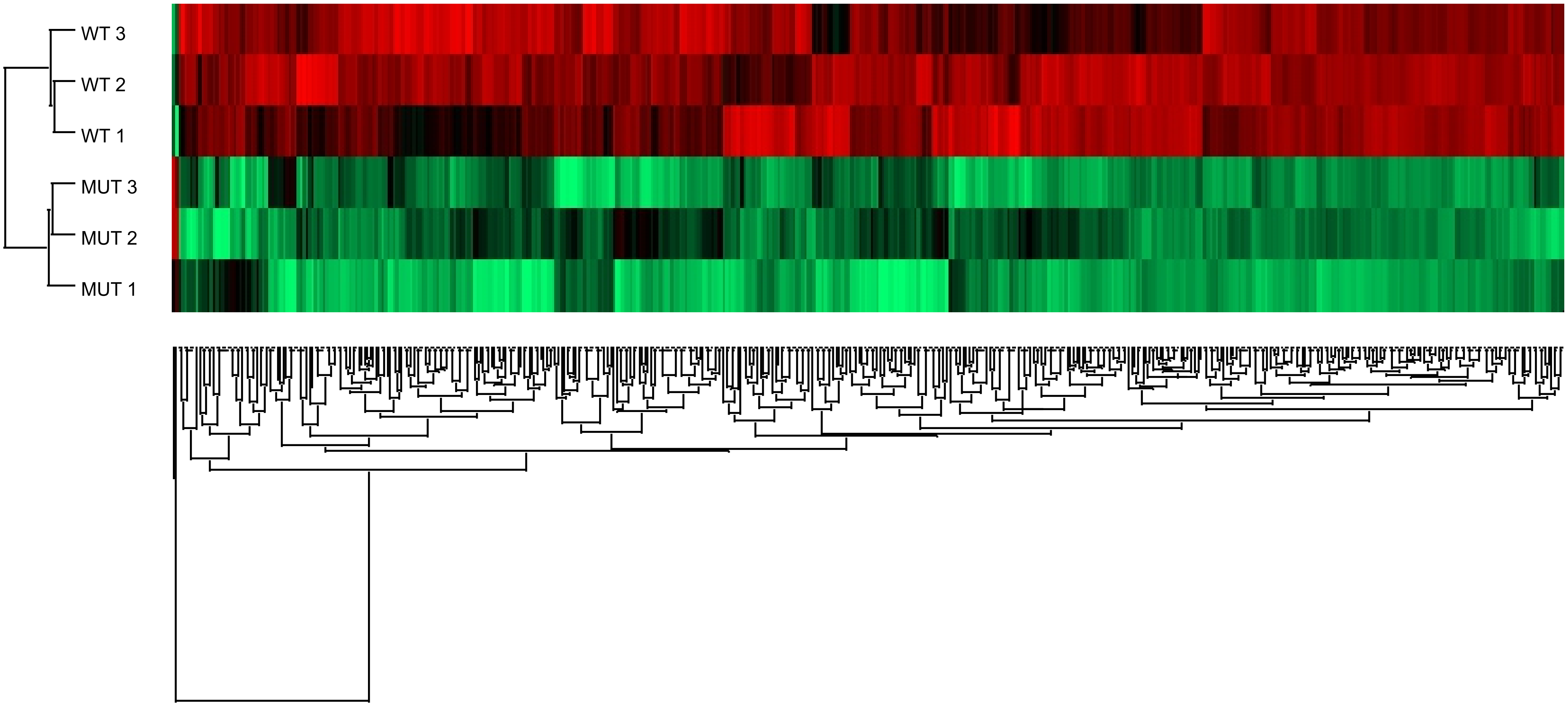

cerebral cortex - phosphoproteins

Supplementary figure 3A

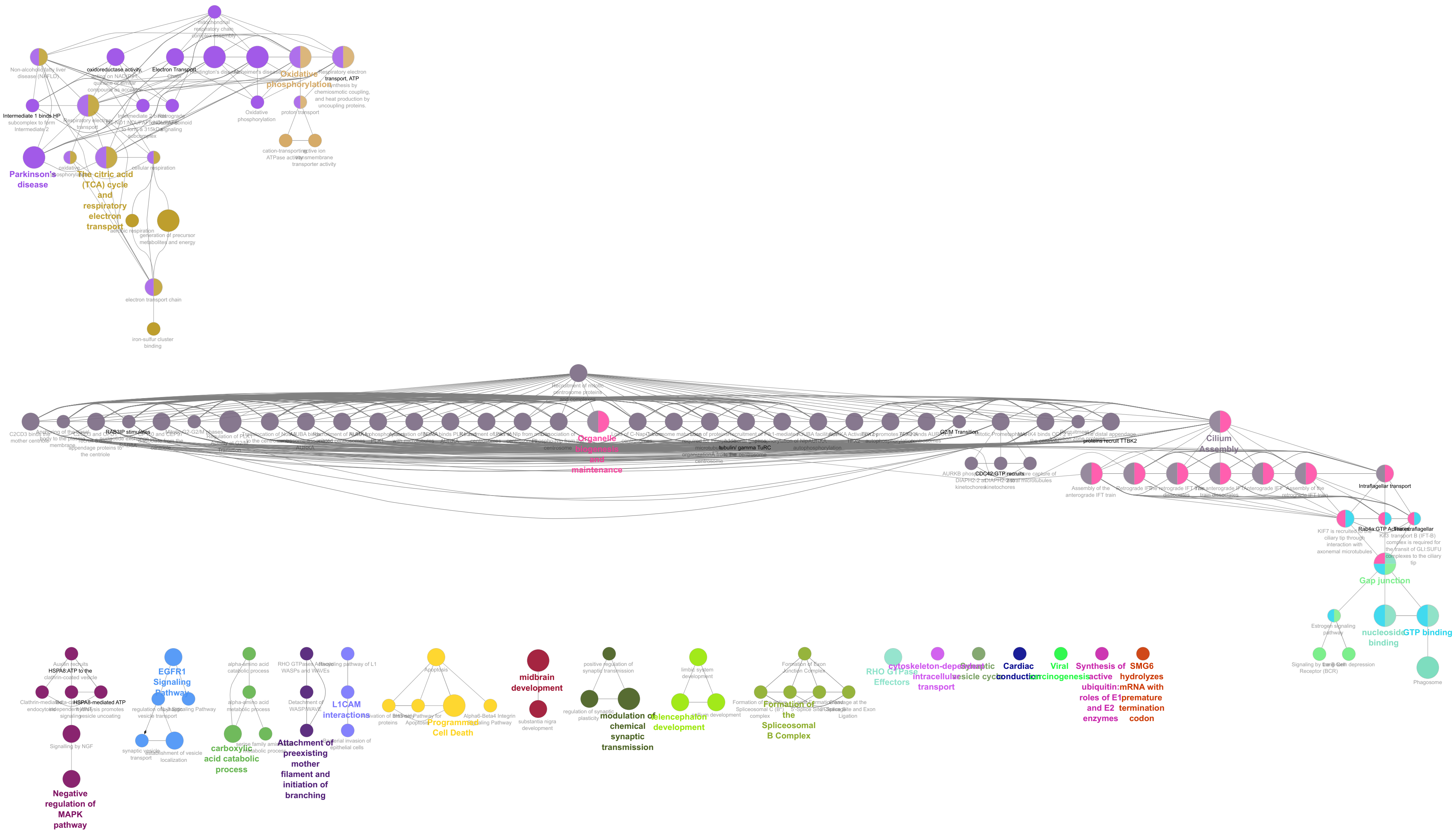

cerebellum
total proteins

Supplementary figure 3B

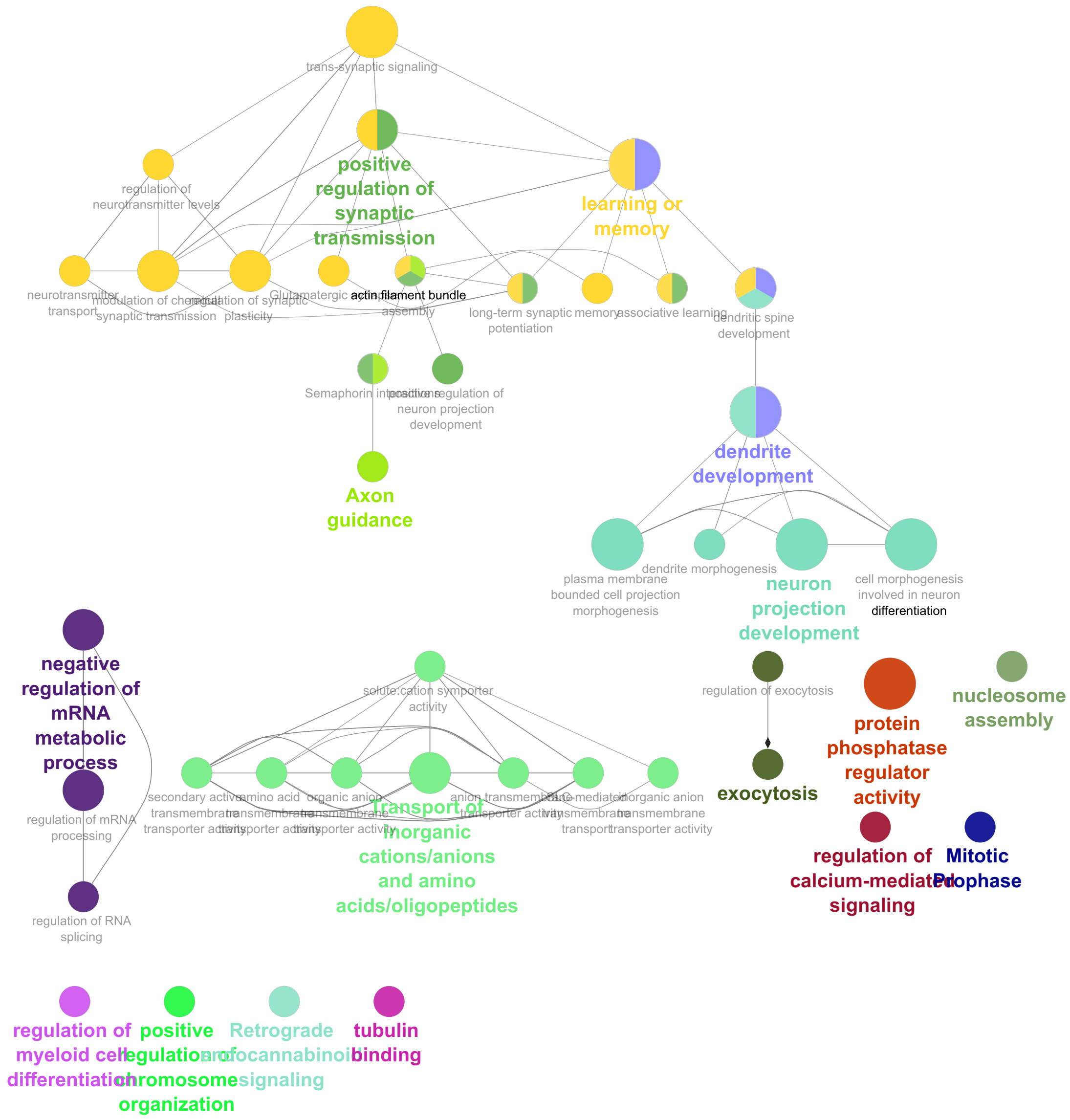

cerebellum
phosphorylated proteins

Supplementary figure 3C

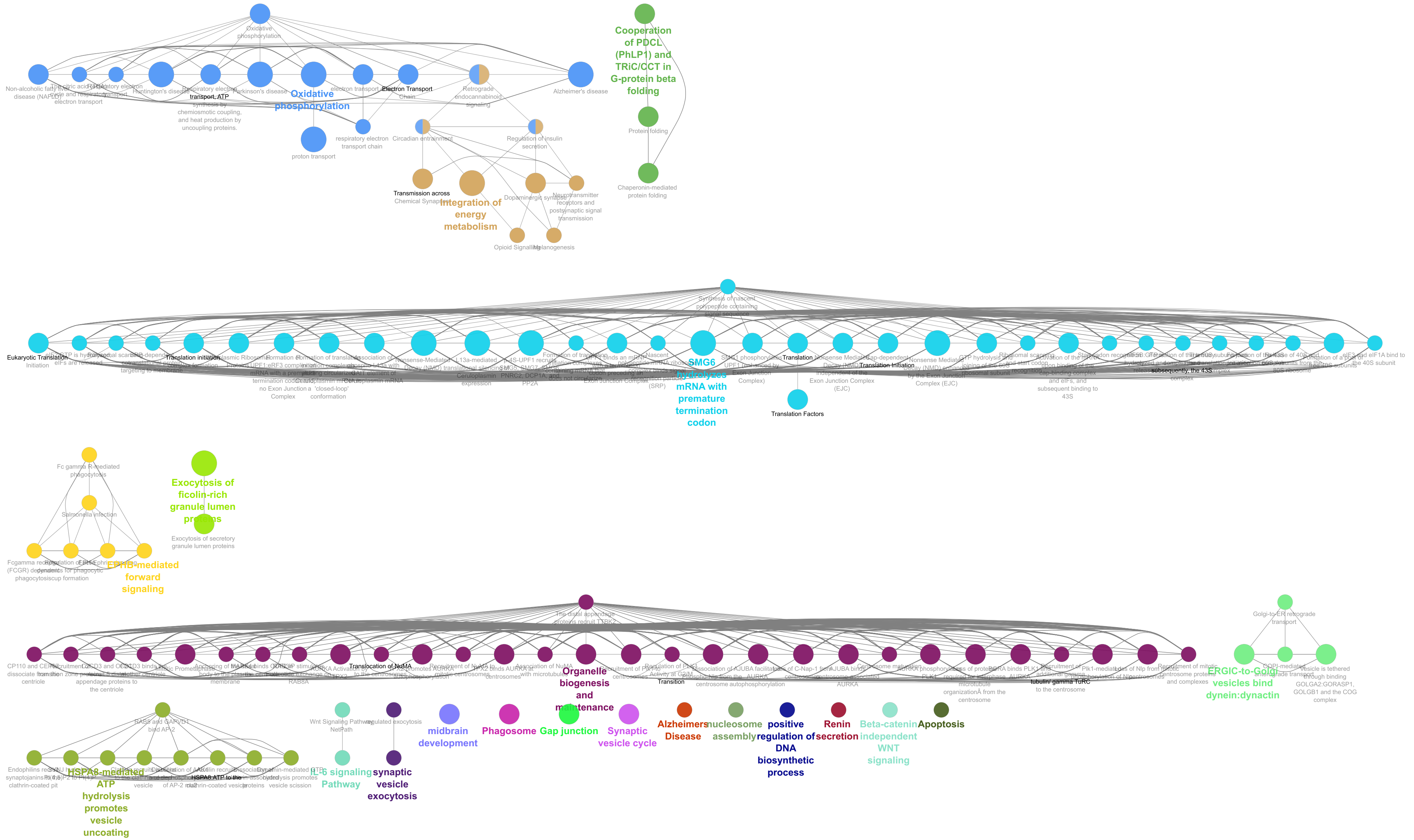

cerebral cortex

total proteins

Supplementary figure 3D

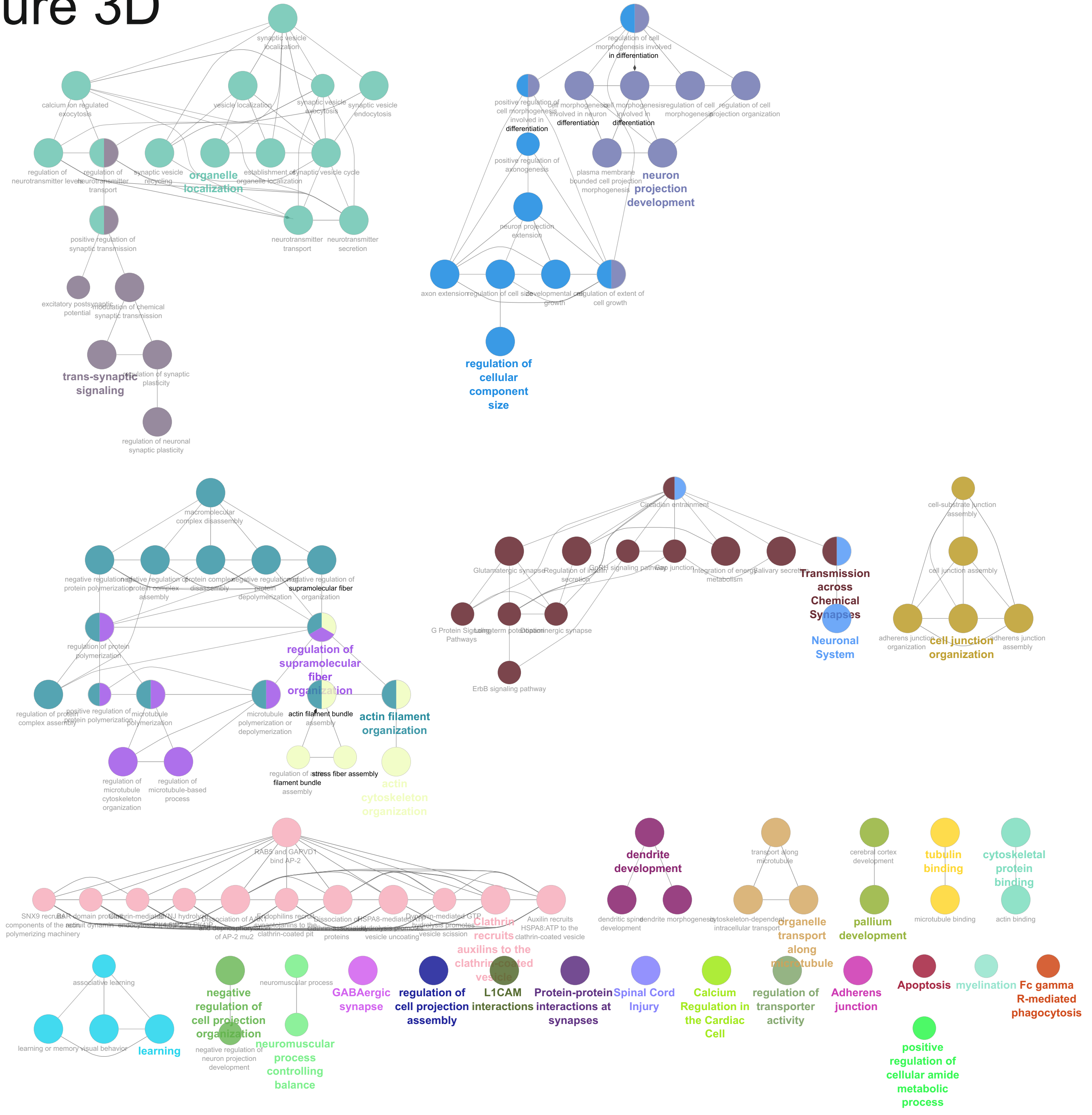

cerebral cortex
phosphorylated proteins

Supplementary figure 4

A

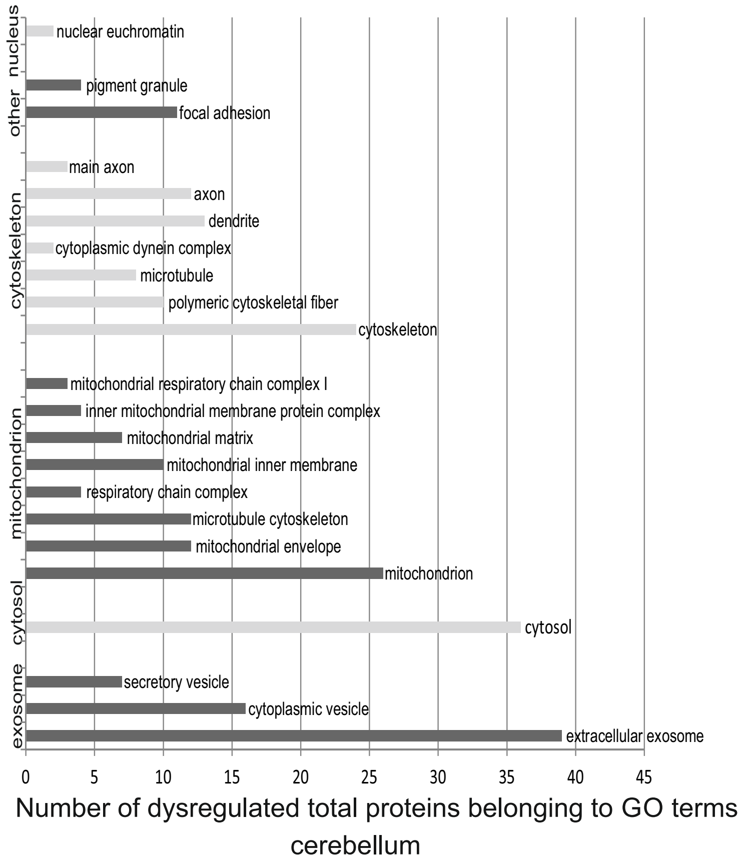

B

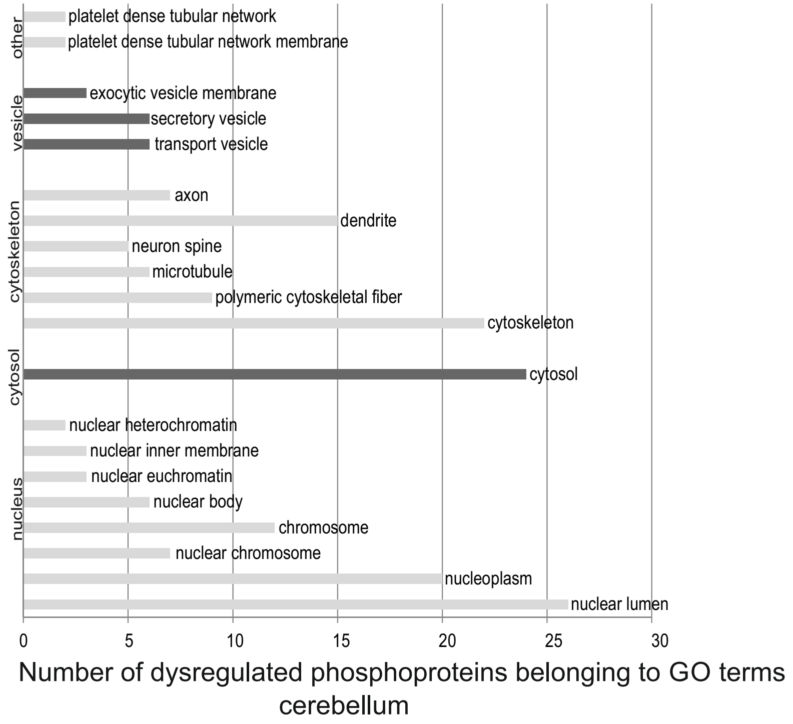

Supplementary figure 4

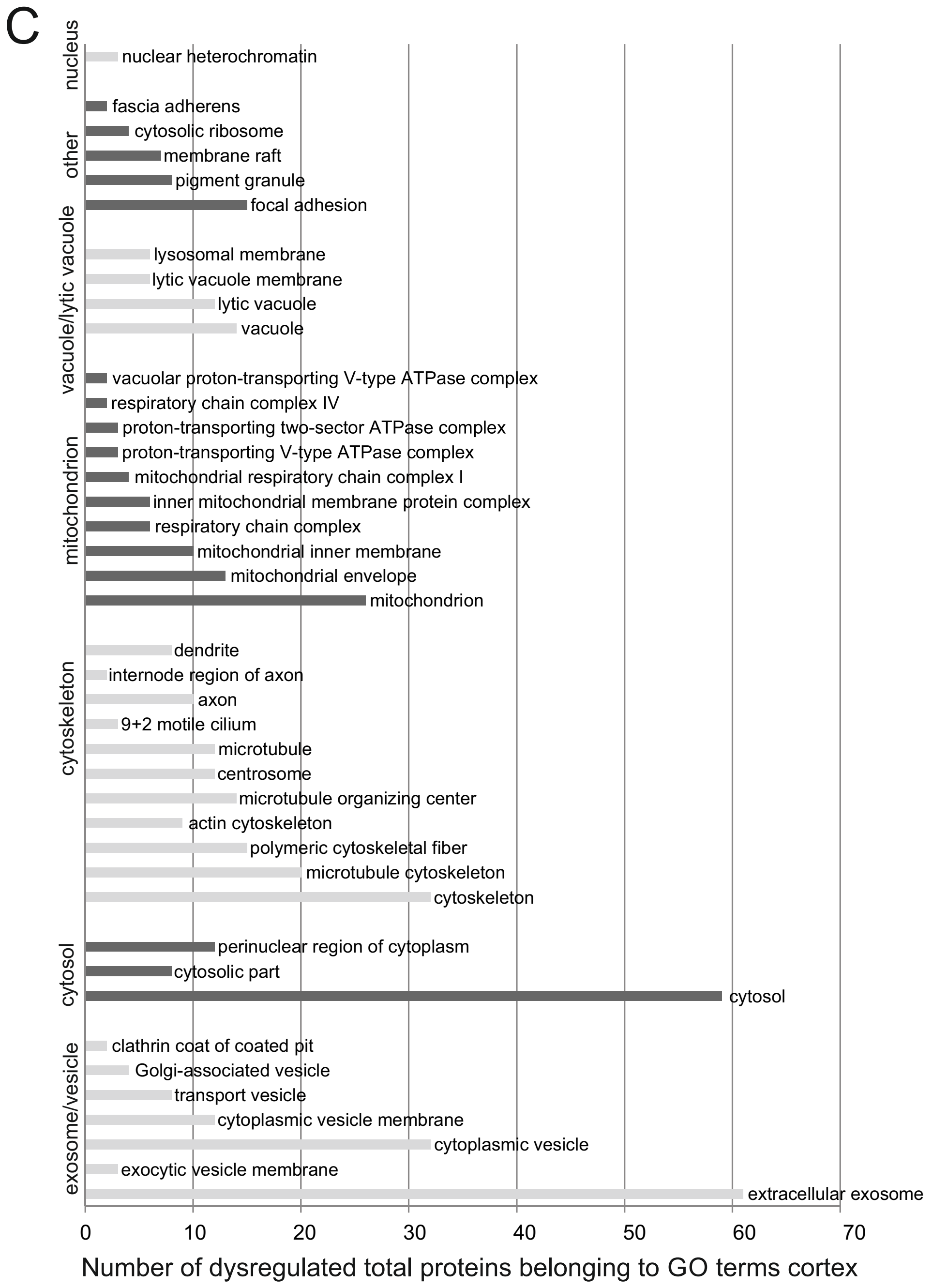

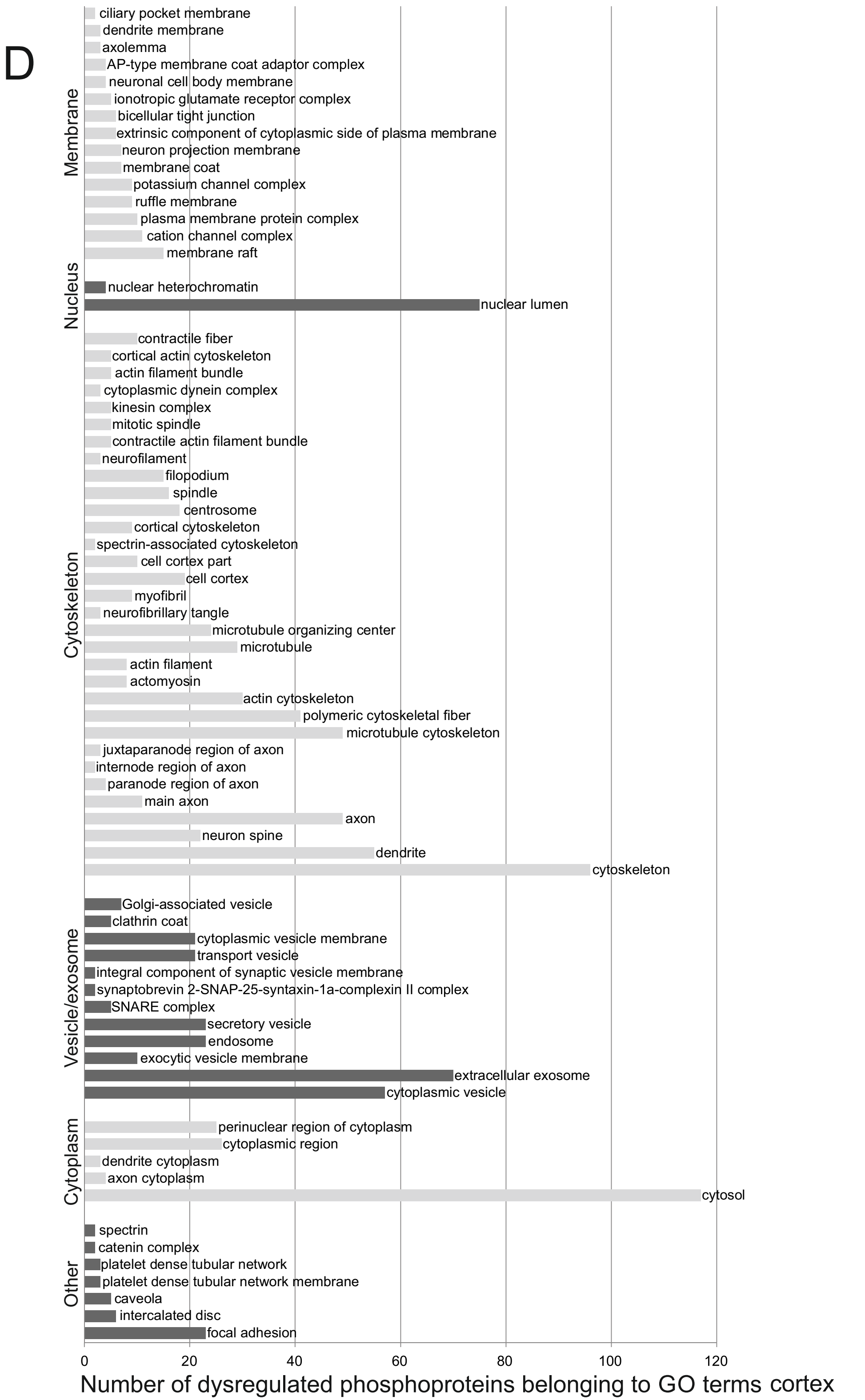

Supplementary Figure 5

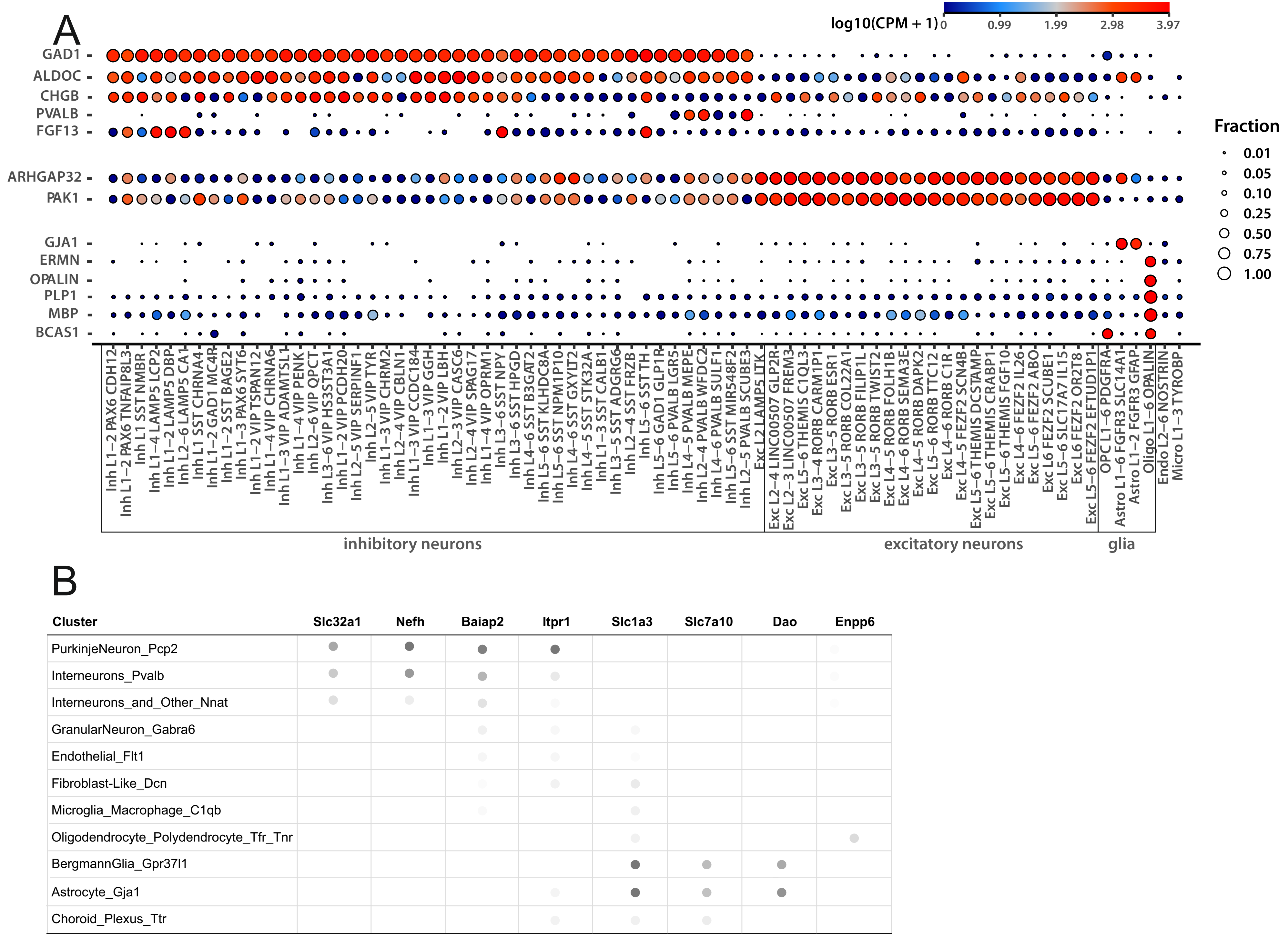

Supplementary figure 6

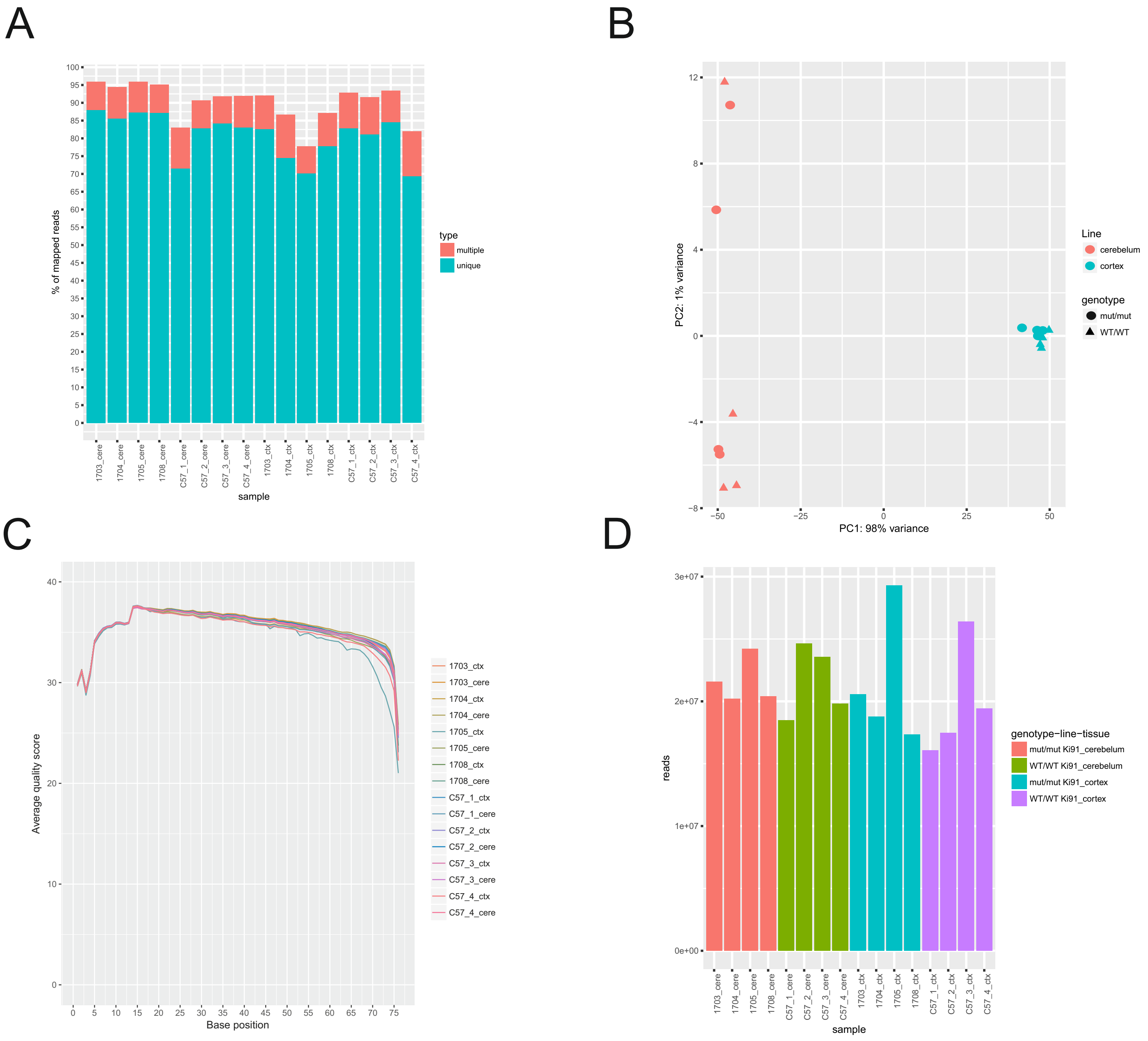

Supplement: Supplementary file 1 — (PDF 3747 kb) [file 12035_2019_1643_MOESM1_ESM.pdf]
