# Supplementary material for: Altered Levels of Proteins and Phosphoproteins, in the Absence of Early Causative Transcriptional Changes, Shape the Molecular Pathogenesis in the Brain of Young Presymptomatic Ki91 SCA3/MJD Mouse
Source: Mol Neurobiol. 2019 Jun 14;56(12):8168–202. doi: 10.1007/s12035-019-01643-4 (PMC6834541; doi:10.1007/s12035-019-01643-4)
Supplement: Supplementary file 2 — (DOCX 36 kb) [file 12035_2019_1643_MOESM2_ESM.docx]

# Supplementary Methods:

## Genotyping

The genotype and the number of CAG repeats were determined by PCR using DNA from a tail biopsy prepared using EZ-10 Spin Column Genomic DNA Minipreps kit (Bio Basics, Markham, Canada). Genotyping was performed by PCR using GoTaq Flexi DNA Polymerase (Promega, Madison, WI, USA). All primers were summarized in Supplementary Table 2. The PCR was done with the following conditions: 3 min 94°C, 12 cycles of (35 s at 94°C, [45 s at 64°C-0.5°C/cycle], 45 s at 72°C), 25 cycles of (35 s at 94°C, 39 s at 58°C, 45 s at 72°C) and 2 min at 72°C. The reaction products were resolved on 1.3% agarose gel in standard TBE buffer and stained with ethidium bromide. The number of CAG repeats was assayed by performing PCR using the 6-FAM-labeled forward primer at conditions of 5 min at 94°C, 36 cycles of (20 s at 94°C, 30 s at 60°C, 30 s at 72°C) and 7 min at 72°C. Subsequently, the labeled PCR fragments were sized using the ABI 3130 xl capillary analyzer and Peak Scanner 1.0 software (Live Technologies, Carlsbad, USA). The PCR product standards with CAG repeats of 21, 69 and 91 repeats were used to perform standard curve.

## Library synthesis and next generation seq.

Cortex and cerebellum dissected from the mouse brain were quickly placed in 500 ul of TRI reagent (MRC, Cincinnati, OH, USA) and immediately snap frozen on dry ice and stored in -80°C. Tissue was homogenized with use of hand homogenizer for 20 s. RNA extraction was performed according to TRI regent manufacturer protocol with additional precipitation using sodium citrate before ethanol wash. Initial quantity and quality of isolated RNA were assessed by micro volume spectrophotometry. RNA was further analyzed with Bioanalyzer 2100 and RNA 6000 Nano kit (Agilent Technologies, Santa Clara, CA, USA).

The transcriptome analyses using next generation sequencing (RNAseq) were performed for Ki91 and control tissues for both cortex and cerebellum and libraries were prepared from 5 μg of total RNA with RIN ≥ 8, using KAPA Stranded mRNA-Seq Kit (Kapa Biosystems, Wilmington, MA, USA) and NEBNext Multiplex Oligos for Illumina (New England Biolabs, Ipswich, MA, USA). Ten pM-indexed libraries were sequenced with the use of Illumina HiSeq2000, rapid run with single 75-nt-long reads. On average, 20 mln single-end reads were collected per library.
