# Supplementary material for: Altered Levels of Proteins and Phosphoproteins, in the Absence of Early Causative Transcriptional Changes, Shape the Molecular Pathogenesis in the Brain of Young Presymptomatic Ki91 SCA3/MJD Mouse
Source: Mol Neurobiol. 2019 Jun 14;56(12):8168–202. doi: 10.1007/s12035-019-01643-4 (PMC6834541; doi:10.1007/s12035-019-01643-4)
Supplement: Supplementary file 3 — List of the animals sacrificed for experiments (DOCX 22 kb) [file 12035_2019_1643_MOESM3_ESM.docx]

Supplementary Table 1. List of the animals sacrificed for experiments

| Number of Animals | Genotype | Age | Tissue | Experiment type |
| --- | --- | --- | --- | --- |
| 8 | 4 x control, 4 x Ki91 SCA3/MJD | 2 months | cortex, cerebellum | RNAseq |
| 8 | 4x control, 4xKi91 SCA3/MJD | 2 months | cortex, cerebellum | proteomics |
| 8 | 4x control, 4x Ki91 SCA3/MJD | 2 months | cortex cerebellum | phosphoproteomic |
| 24 | 4 x C57BL, 4 x FVB, 4 x Ki91 SCA3/MJD | 2 and 4  months | cortex cerebellum | validation of RNAseq (qPCR) |
| 16 | 4 x C57BL, 4 x Ki91 SA3/MJD | 10 and 14 months | cortex cerebellum | Detection of late mRNA deregulation (qPCR) |
| 8 | 4 x control, 4 x Ki91 SCA3/MJD | 2 months | whole brains | immunohistochemistry |
| 36 | 18 x control, 18xKi91 SCA3/MJD | 2 months | - | behavior |
