# Supplementary material for: Altered Levels of Proteins and Phosphoproteins, in the Absence of Early Causative Transcriptional Changes, Shape the Molecular Pathogenesis in the Brain of Young Presymptomatic Ki91 SCA3/MJD Mouse
Source: Mol Neurobiol. 2019 Jun 14;56(12):8168–202. doi: 10.1007/s12035-019-01643-4 (PMC6834541; doi:10.1007/s12035-019-01643-4)
Supplement: Supplementary file 4 — Primers used for qPCR validation of RNA sequencing result and designed based on proteomic analysis data (DOCX 25 kb) [file 12035_2019_1643_MOESM4_ESM.docx]

Supplementary Table 2. Primers used for qPCR validation of RNA sequencing result and designed based on proteomic analysis data

| Gene | Forward primer (5’-3’) | Reverse primer (5’-3’) |
| --- | --- | --- |
| Mouse | | |
| Slc38a6 | GAAGATCTGGGACTCTTCGCTT | TTTCCCCGTGACGTCAACTC |
| Ttc8 | ATGCCACCTTCCACTTGACTG | TTCCTCACACCTTTGACAGCC |
| Prdx3 | GTGGTTTGGGCCACATGAAC | GGGACTCTGGTGTCCAGTTG |
| Ccdc88c | GCCCAGGGGATCTAAAACCA | TCCGAGGATGGCTCACAGA |
| Ide | AAAGACACTTACCAAGGACGAT | TCCCTGGCAAGAACATGGAC |
| Btaf1 | TTCGACATGGGGCAGGTACT | GCTGTCTCCCATTTTACCACC |
| Btaf1 | TTTAGTCCCACGTGTCTGGC | TCCAGAATTTCCTGGCTGCTT |
| Ablim1 | CGCTTCCCAGGGTGAAAGAT | CCCTCTACCTCCCCCATCAA |
| Serpina3n | GGGATGATCAAGGAACTGGTC | CGTGTCAAGAGGGTCAAAGG |
| Idh1 | AGGAGGTTCTGTGGTGGAGA | ACCTGGTCATTGGTGGCATC |
| Atp2b1 | TAGAGCAATGGCTGTGGTCG | TGGGTTCCATGACCAGCTTC |
| Akr1b1 | AGAGCATGGTGAAAGGAGCC | GGTATCACGTTCCCTGAGGC |
| Ca2 | TAAAGCTGCGTCCAAGAGCA | CCATCAGATGAGCCCCAGTG |
| Ppp2r1a | GGCCTGAGTATGTGCACTGT | GCCTTGTCTCGCACTACTGT |
| Plp1 | GGCTAGGACATCCCGACAAG | TACATTCTGGCATCAGCGCA |
| Cox7a2 | GTGGGTAACAACCGAGCCAA | TCGTGAAGTGGTGCTGATGG |
| Ndufa9 | CTCGAGCAATAGCTCAGGCA | AGACGGCCGTATGATGATGG |
| Nefh | CGCCCTCAAGTGCGACG | CGCCGGTACTCAGTTATCTCC |
| Omg | CCAGAAACCCTTCCGACTCC | GCATAAGATGCCAGGCGTGA |
| Srsf2 | TCTGCCCGAAGATCCAAGTC | GCTTGCCGATTCATCATTTTCT |
| Psmd4 | AGGAGGCAAGATGGTGTTGG | GTGTGATCAGGCCCACGTTA |
| Qdpr | ACCCTGGATACCCCGATGAA | GAGTTTGGCCGTTTGTTCCC |
| Reln | CGTCCTAGTAAGCACTCGCA | TATCGCCTAAGCGACCTTCG |
| Sst | GACCCCAGACTCCGTCAGTT | GGTTCGAGTTGGCAGACCTC |
| Npy | GACCCTCGCTCTATCTCTGC | GGGCTGGATCTCTTGCCATA |
| Cd68 | GGGGCTCTTGGGAACTACAC | ATGCCCCAAGCCTTTCTTCC |
| Cldn11 | ACCTGCCGAAAAATGGACGA | ACGTAGCCTGGAAGGATGAGG |
| Mag | TCGCCTTTGCCATCCTGATT | GTTGTCTCCCCCTGAGAAGC |
| Plekhb1 | AAATTCCACCCCGGTACGC | AAGGTGGCCCATAGTAGCTG |
| Syp | TTTGCCATCTTCGCCTTTGC | TAGTGCCCCCTTTAACGCAG |
| Pdgfra | GCACCAAGTCAGGTCCCATT | TGTCCAGGTCTTTCTTCGGC |
| Olig1 | CCGCCCCAGATGTACTATGC | AACCCACCAGCTCATACAGC |
| Olig2 | GTACCTGGGGGCTTGACAAA | AACAAAGAGCTTCGCATCGC |
| Mash1 | CTCCTGGGAATGGACTTTGGA | GTGAAGGTGCCCCTGTAGGT |
| Pea15a | CTCAAGTCAGCCTGCAAGGA | CAGCACACGGGTTCTGTAGT |
| Pgk1 | ATGTCGCTTTCCAACAAGCTG | GCTCCATTGTCCAAGCAGAAT |
| Tfrc | GCCCCAGAAGATATGTCGGAA | TCATGAGGGAAATCAATGATCGTA |
| Actin | TTCTTTGCAGCTCCTTCGTT | ATGGAGGGGAATACAGCCC |
| Human | | |
| Olig1 | GTCGCAGAGAGTTTTCGCTC | ACTGTGAGACGCCTGGTTAC |
| Olig2 | TCCTCCCTGTCTCTCGTTGA | AGATGAGTCGGTGGGGTAGT |
| Qdpr | ACAGACTCGTTCACTGAGCA | GATGGCTGGAGATGGTCGAT |
| Omg | AGCTCACAGCAACACAATGC | ATCAAAGCCGTGGTGTCGTC |
| Mag | CAACCCTCCCGTCCTGTTC | GGTAGAAACCTCTTTGGACTCGT |
| Cd68 | CAGGGAATGACTGTCCTCACAA | AGTGCTCTCTGTAACCGTGGG |
| Cldn11 | CTTGGCACTGTAGCATGTGGA | GCACAAAGCAGGCAGTCAAC |
| Gad | ATTGCACCAGTGTTTGTCCTC | CTTGTAGCGAGCAGCCATGA |
| Ca2 | GCCAAGTATGACCCTTCCCT | TGTAAGTGCCATCCAGGGGT |
| Srsf2 | GGAATCCAAATCCAGGTCGC | CCGAGCAGCACTCCTAATGAT |
| Actin | CCAACCGCGAGAAGATGA | CCAGAGGCGTACAGGGATAG |
